# Interplay between host genetics and gut microbiome composition in the Japanese population

**DOI:** 10.3389/frmbi.2025.1635907

**Published:** 2025-10-14

**Authors:** David Ortega-Reyes, Tadashi Takeuchi, Yusuke Ogata, Takuro Iwami, Wataru Suda, Tetsuya Kubota, Naoto Kubota, Takashi Kadowaki, Kohei Tomizuka, Hiroshi Ohno, Momoko Horikoshi, Chikashi Terao

**Affiliations:** ^1^ Laboratory for Statistical and Translational Genetics, RIKEN Center for Integrative Medical Sciences, Yokohama, Japan; ^2^ Laboratory for Genomics of Diabetes and Metabolism, RIKEN Center for Integrative Medical Sciences, Yokohama, Japan; ^3^ Laboratory for DNA Data Analysis, National Institute of Genetics, Shizuoka, Japan; ^4^ Department of Genetics, School of Life Science, The Graduate University for Advanced Studies, SOKENDAI, Kanagawa, Japan; ^5^ Laboratory for Intestinal Ecosystem, RIKEN Center for Integrative Medical Sciences, Yokohama, Japan; ^6^ Laboratory for Microbiome Sciences, RIKEN Center for Integrative Medical Sciences, Yokohama, Japan; ^7^ Department of Orthopedic Surgery, Keio University School of Medicine, Tokyo, Japan; ^8^ Laboratory for Symbiotic Microbiome Sciences, RIKEN Center for Integrative Medical Sciences, Yokohama, Japan; ^9^ Department of Diabetes and Metabolic Diseases, Graduate School of Medicine, The University of Tokyo, Tokyo, Japan; ^10^ Division of Diabetes and Metabolism, The Institute for Medical Science Asahi Life Foundation, Tokyo, Japan; ^11^ Department of Clinical Nutrition, National Institutes of Biomedical Innovation, Health and Nutrition (NIBIOHN), Tokyo, Japan; ^12^ Department of Metabolic Medicine, Faculty of Life Sciences, Kumamoto University, Kumamoto, Japan; ^13^ Toranomon Hospital, Tokyo, Japan; ^14^ Laboratory for Immune Regulation, Graduate School of Medical and Pharmaceutical Sciences, Chiba University, Chiba, Japan; ^15^ Graduate School of Medical Life Science, Yokohama City University, Yokohama, Japan; ^16^ Clinical Research Center, Shizuoka General Hospital, Shizuoka, Japan; ^17^ Department of Applied Genetics, School of Pharmaceutical Sciences, University of Shizuoka, Shizuoka, Japan

**Keywords:** host genetic variation, gut microbiome, whole-genome sequencing (WGS), 16S rRNA sequencing, shotgun metagenomic sequencing, genome-wide association studies (GWAS), phenome-wide association studies (PheWAS)

## Abstract

**Background:**

Host genetics significantly influence the composition of the gut microbiota, but this relationship remains poorly understood, especially in non-European populations. This study aims to investigate the associations between host genetic variation and gut microbiome composition in the Japanese population and to assess methodological factors affecting reproducibility in microbiome research.

**Methods:**

We performed whole-genome sequencing on 306 Japanese individuals and obtained their gut microbiome profiles using shotgun metagenomic sequencing. Genome-wide association studies (GWAS) were conducted to identify associations between host genetic variants and the relative abundance of microbial taxa and bacterial pathways. Phenome-wide association studies (PheWAS) were performed on predicted high-impact variants. Additionally, we compared methodological approaches to assess their impact on microbiome composition and reproducibility.

**Results:**

We identified significant associations between host genetic variants and the relative abundance of one bacterial family, one genus, one species and eight bacterial pathways (*p* ≤ 5×10^−8^). However, none of these associations surpassed the stringent significance threshold of *p* ≤ 2.75×10^−11^. Notably, we were unable to replicate associations reported in prior studies, including those conducted in Japanese populations, even regarding the direction of effects. Our PheWAS analysis uncovered a frameshift variant in the *OR6C1* gene (rs5798345-CA) that was significantly associated with an increased abundance of *Bacteroides uniformis*. Furthermore, comparative analyses highlighted that methodological differences, particularly in sample processing and DNA extraction protocols, substantially influence the observed gut microbiome composition. This variability may be a key factor contributing to the lack of reproducibility across studies.

**Conclusion:**

Our findings enhance the understanding of how host genetics shape the gut microbiota in the Japanese population and underscore the importance of methodological standardization in microbiome research. The identified associations between host genetic variants and specific microbial taxa provide insights into the complex interplay between genetics and the gut microbiome. Addressing methodological discrepancies is crucial for improving reproducibility and advancing knowledge of host–microbiome interactions.

## 1 Introduction

The gut microbiota is a complex ecosystem consisting of bacteria, archaea, fungi, protozoa, and viruses that plays a crucial role in human health and disease (Thursby and Juge, 2017; Kho and Lal, 2018; Wu et al., 2021). Research has demonstrated that host genetics can significantly influence the composition of the gut microbiota (Cahana and Iraqi, 2020; Qin et al., 2022). For instance, monozygotic twins have been found to exhibit similarities in microbial communities, suggesting a genetic component (Goodrich et al., 2014; Goodrich et al., 2016; Vilchez-Vargas et al., 2022). Moreover, specific heritable bacterial taxa have been identified, further supporting the influence of host genetics on the gut microbiota (Goodrich et al., 2014; Ishida et al., 2020). However, most genome-wide association studies (GWAS) have primarily focused on European populations (Davenport et al., 2015; Scepanovic et al., 2019; Rühlemann et al., 2021; Lopera-Maya et al., 2022; Qin et al., 2022), leaving the genomic basis of the microbiota in other populations largely unknown. Gut microbial composition is diverse among different ethnicities and geographies (Nishijima et al., 2016), emphasizing the importance of host genetics factors in shaping gut microbiota. An important case in point is the Japanese population, where only a single study has examined the association between host genetics and the composition of core genus relative abundance in the gut (Ishida et al., 2020).

While dietary factors, medication, physical activity, and health status have been recognized as influencing the gut microbiota (Leeming et al., 2019; Cella et al., 2021), the impact of host genetics on its composition remains relatively unexplored. The gut microbiota has been implicated in a wide range of diseases, including obesity, celiac disease, Crohn’s disease, ulcerative colitis, gastroenteritis, asthma, and inflammatory bowel disease (Gagliardi et al., 2018; Hills et al., 2019; Gill et al., 2022). Understanding the factors that shape the gut microbiome is critical for developing therapeutic interventions to improve human health. Although environmental factors have been shown to influence gut microbiota composition, the role of host genetics in modulating the gut microbiota remains understudied.

GWAS have successfully identified genetic loci associated with various human traits and diseases (Abdellaoui et al., 2023). Similarly, microbiome GWAS (mGWAS) aims to identify host genetic polymorphisms that interact with the composition and abundance of the gut microbiota. mGWAS have identified significant loci and their connection to bacterial taxa, highlighting the influence of host genetics on microbiome composition in health and disease (Awany et al., 2018).

Despite the progress made in understanding the interplay between host genetics and the gut microbiota, several challenges still need to be addressed. Reproducibility across studies is limited, and associations often lose significance after correction for multiple testing (Awany et al., 2018; Rothschild et al., 2018; Weissbrod et al., 2018). Furthermore, environmental factors such as diet and medication usage appear to have a greater influence on the gut microbiome than identifiable host genetic factors (Qin et al., 2022). To address these challenges, it is crucial to incorporate population-specific cohorts from non-European populations and utilize shotgun metagenomic sequencing to establish strong associations between host genetics and gut microbiota composition at the species level, which still proves to be challenging when relying solely on 16S rRNA-based approaches (Lopera-Maya et al., 2022).

Moreover, building upon the observation that host genetics can shape microbial composition, we turn our attention to the SNP rs671, which is prevalent in East Asian populations and has been associated with both altered alcohol metabolism and susceptibility to metabolic disorders, including type 2 diabetes in men (Moller, 2001; Després and Lemieux, 2006; Spracklen et al., 2020). Some studies have indicated that fecal carbohydrates, particularly host-accessible monosaccharides, are closely linked to insulin resistance (IR) through alterations in gut microbiota while inflammatory cytokines may act as mediators in this relationship (Moller, 2001; Spracklen et al., 2020; Takeuchi et al., 2023). Thus, considering the potential role of rs671 in modulating responses to intestinal substrates and cytokine-mediated inflammation, we also aim to explore how this genetic variant may influence the interplay between fecal carbohydrates, host cytokines, and insulin resistance status in a Japanese population.

This study seeks to address these gaps by investigating the role of host genetics in shaping the gut microbiota composition in a Japanese population. By utilizing a genome-wide approach and considering shotgun metagenome sequencing, this study aims to overcome the limitations of previous research by identifying host genetic associations with the gut microbiota at all taxonomic levels, and its related bacterial pathways, as illustrated in **Supplementary Figure 1**. The findings from this study will contribute to our understanding of the complex interplay between host genetics and the gut microbiota.

## 2 Materials and methods

### 2.1 Study participants and data collection

Participants in this study (Takeuchi et al., 2023) were recruited from 2014 to 2016 during their annual health check-ups at Tokyo University Hospital. The participants were Japanese individuals aged between 20 and 75 years, including both males and females. Exclusion criteria was applied, such as a prior diagnosis of diabetes, routine use of diabetes or intestinal medications, recent antibiotic use, and significant weight loss in the three months prior to sample collection. To ensure comparable clinical characteristics, the study enrolled 101 individuals with normal health, 100 individuals classified as obese (based on the Japanese definition of Body Mass Index [BMI] ≥ 25), and 112 individuals classified as prediabetic (based on FBG ≥ 110 mg/dL and/or HbA1c ≥ 6.0%) using their clinical data. Participants were instructed to fast overnight before their hospital visit, during which clinical information and blood samples were taken in the morning. Blood samples were immediately processed and stored at −80°C. Fecal samples were also collected in the morning, transported to the hospital within 24 hours, and stored at −80°C. Out of the total, 256 participants provided fecal samples on the day of their hospital visit, while the rest collected their samples between 2 days before and 7 days after the visit. A small number of participants either collected their samples too long after the visit, collected the evening before the visit, or did not provide fecal samples. Two individuals withdrew from the study after enrollment. Therefore, a total of 306 individuals underwent physical examination, laboratory tests, and fecal sampling. Fecal metagenomic data were available for 290 individuals due to limited samples.

### 2.2 Host whole-genome sequencing data generation

Genomic DNA was extracted from peripheral blood samples of 306 individuals using standard laboratory procedures. Two different sequencing technologies were employed: Illumina HiSeqX Five/Ten was used to sequence 155 samples (conducted by Macrogen Japan Corporation [https://macrogen-japan.co.jp] in 2017 and 2018), and Illumina NovaSeq was used to sequence 156 individuals (by RIKEN sequencing platform in 2020). Both Illumina HiSeqX Five/Ten and NovaSeq platforms generated 150 bp paired-end reads and a mean depth of 18.4x. Out of the 311 samples sequenced, five were duplicates, sequenced with both platforms. Variant calling was performed using Dragen Bio IT-platform v3.5.7 (Illumina) with the GRCh38 human reference genome. Joint calling was performed with Dragen Bio IT-platform v3.6.3 (Illumina) and excluded low quality variants (QUAL<10.41 for SNPs and QUAL<7.83 for InDels) following Dragen default parameters. The joint called VCF files were then “lifted down” from the GRCh38 to the GRCh37 reference genome for downstream analyses.

### 2.3 Whole-genome sequencing quality control

#### 2.3.1 Sample quality control

Per-sample QC of joint-called VCF files was performed on 306 individuals using Plinkv1.9 (Purcell et al., 2007). We first removed five duplicate individuals that were sequenced twice with both Illumina HiSeqX and NovaSeq. Then, conducted a gender check analysis by setting homozygosity thresholds of >0.8 for men and <0.2 for women. All individuals passed this threshold. Moreover, we identified samples of poor quality based on their call rate (>90%) and heterozygosity (<4 standard deviations from the mean). We removed five individuals that were over 4 standard deviations from the mean in terms of heterozygosity. We also evaluated population structure by combining the genotypes of our study with a reference dataset (1000 genomes phase 3) consisting of individuals with known ethnicities (Auton et al., 2015). We applied principal component analysis (PCA) to identify individuals with divergent ancestry. One sample was excluded based on the results of PCA. Lastly, we assessed genetic relatedness by calculating the relatedness between each pair of samples. We removed the samples with the lowest call rate that had a degree of relationship over 25% with their pair. After performing these per-sample QC analyses, we ended up with 296 QCed individuals.

#### 2.3.2 Per-marker quality control

Per-marker QC was performed on approximately 15.8 million variants from the 296 QCed samples using Plinkv1.9 (Purcell et al., 2007). We split and kept autosomal information and excluded variants with a missing genotype rate greater than 0.05 (i.e., call rate less than 0.95) and variants that deviated from Hardy–Weinberg Equilibrium (HWE *p* ≤ 1×10^−6^). This resulted in approximately 12.98 million QCed variants.

### 2.4 Compositional data generation from shotgun metagenome sequencing

#### 2.4.1 Preparation of fecal samples and DNA extraction from fecal samples

Preparation and DNA extraction from fecal samples was performed by a previous study (Takeuchi et al., 2021; Takeuchi et al., 2023).

#### 2.4.2 Shotgun metagenomic sequencing

Metagenome shotgun libraries were prepared and sequenced by a previous study (Takeuchi et al., 2023). For which they filtered out reads mapped to human and bacteriophage genomes, the remaining reads were assembled, and protein-coding genes were predicted. A total of 6,458,217 non-redundant genes were identified across the samples. Functional assignment of these genes was performed using DIAMOND against the KEGG database, resulting in the identification of KEGG orthologues. Eukaryotic genes were excluded from further analysis.

#### 2.4.3 Quantification of annotated genes in human gut microbiomes

Taxonomic assignment of metagenomic reads was performed by a previous study (Takeuchi et al., 2023). From this, metagenomic operational taxonomic units (mOTUs) analysis was performed on one million filter-passed reads to determine the relative abundance of species (Edgar, 2018). The predicted genes were functionally annotated by mapping one million filter-passed metagenomic reads to a combined reference gene set. Multi-mapped reads were normalized based on the proportion of uniquely mapped reads to these genes. The proportion of KEGG orthologues (KOs) was calculated from the mapped reads. Enrichment analysis of KEGG pathways was conducted by assigning positive and negative scores to associated KOs and summarizing the points as a ratio to the total number of KOs in the pathway.

### 2.5 Microbiome taxonomic relative abundance filtering and transformation

#### 2.5.1 Filtering of taxa and bacterial pathways

We selected relative abundance taxonomic data at the phylum, class, order, family, genus, species and bacterial pathway levels from shotgun metagenome sequencing analysis. Raw relative abundance data from each taxonomic level were filtered based on the prevalence of each taxon in the whole sample and the abundance ratio of each taxon in each sample. We retained taxa that had a prevalence of over 25%, indicating that the taxa were present in more than 25% of the individuals, and the core relative abundance of each taxonomic level that explained over 90% of the total relative abundance in our cohort. Furthermore, we excluded pathways that were not associated with bacteria, as they may have originated from Eukaryotic pathways during the annotation process.

#### 2.5.2 Transformation and normalization of raw filtered relative abundance data

Transformation and normalization were carried out using two approaches: centered-log ratio transformation (CLR) and direct rank-based inverse normal transformation (INT). For CLR, we directly applied it to the raw relative abundance data using the R compositions package. For INT, we applied INT using R, adding a pseudo-count of 0.1 to handle zero values. We visualized the output distributions from both transformations by plotting them into histograms. We selected the transformed and normalized data that were closer to a normal distribution for downstream analysis. In this case, the relative abundance INTed data was selected.

#### 2.5.3 Identification of binary taxa

To determine the appropriate statistical models for GWAS, we assessed the distribution of each taxon. Histograms were created for all taxa and bacterial pathways post-filtering to visualize their distributions after INT. To further identify non-normally distributed taxa, we employed the Shapiro–Wilk test (Shapiro and Wilk, 1965) using the shapiro.test function from the R stats package. The Shapiro–Wilk test evaluates how closely a dataset follows a normal distribution by calculating a W-statistic, which measures the correlation between the observed data and the expected values under a normal distribution. The W-statistic is computed using the formula:


$$W=\;\frac{({\sum_{i=1}^{n}a_{i}x_{\left(i\right)})}^{2}}{{\sum_{i=1}^{n}\left(x_{i}-\bar{x}\right)}^{2}}$$

Where:

*x*_(_ *_i_*_)​_ are the ordered sample values (smallest to largest),

*a_i_* are constants derived from the expected values of a normal distribution,

*x_i_* are the original sample values,

*x*ˉ is the sample mean.

A W-statistic of 1 indicates perfect normality, while lower values suggest deviations from normality. For our analysis, we defined a threshold of W ≥ 0.95 to indicate sufficient normality. This threshold balances the risk of false positives (treating non-normal data as normal) and false negatives (rejecting data that is sufficiently normal for analysis). Taxa with W-statistics below this threshold were considered non-normally distributed and were more appropriately analyzed as binary traits. This approach ensures that linear regression is applied only to taxa with distributions that are reasonably normal, improving the robustness of the statistical models.

### 2.6 Genome-wide association between host genetics and microbiome data

#### 2.6.1 Genome-wide association

GWAS was performed between host genetics (QCed genotype) and INTed transformed relative abundance of each taxonomic level and bacterial pathway from shotgun metagenome sequencing. Following the normality assessment using the Shapiro–Wilk test, for the taxa that met the normality threshold (W ≥ 0.95), we conducted quantitative GWAS using linear regression with Plink v2.0’s (Chang et al., 2015) generalized linear model command. While for taxa that did not meet the Shapiro–Wilk normality threshold, indicating non-normal distributions, we conducted GWAS for these as binary taxa using logistic regression models appropriate for binary traits implemented in PLINK version 2.0. Each taxon was selected as a dependent variable (phenotype), and each genetic variant from the host was considered as an independent variable (genotype). Covariates such as age, sex, sequencing batch, clinical group (normal, obese, or prediabetic), and the first 10 principal components were included. Variants that passed the significance threshold (*p* < 5×10^−8^) were considered to be significantly associated with the tested taxon. To account for multiple testing, a correction was performed by dividing the nominal significance threshold of 0.05 by the number of tests (12,985,047 variants × number of taxa and pathways), resulting in a corrected p-value of 2.75×10^–11^ for the quantitative GWAS and a p-value of 9.32×10^–11^ for the binary GWAS. Manhattan and QQ plots were generated using the R qqman package to visualize the significant associations. Additionally, lambda was calculated in R to adjust for genomic control.

#### 2.6.2 Tree-based visualization of significant associations

Genera were classified based on their raw relative abundance data using the R package Metacoder (Foster et al., 2017). This allowed us to create a heat-tree visualization of the taxonomic diversity in our sample. By using this heat-tree, we were able to locate the taxa with significant associations and qualitatively assess the abundance of each and the proximity between them.

### 2.7 Comparative analysis of methodologies for generating relative abundance data between studies

In an attempt to understand the lack of correlation at the genus level between Ishida et al. (2020) study and ours, we utilized two methods to process fecal samples from five healthy subjects, in duplicate, for microbial analysis using 16S rRNA sequencing.

The first method, replicated from Ishida et al., involved preserving the fresh fecal sample in GuSCN solution, vortexing with glass beads, and treating with buffer-saturated phenol. After centrifugation, the supernatant was further extracted with phenol-chloroform and precipitated with isopropanol. The DNA, extracted by this bead-beating method, was then subjected to 16S rRNA sequencing. The resulting sequences were classified into operational taxonomic units (OTUs) using QIIME v2 (Bolyen et al., 2019) for analysis and Greengenes (DeSantis et al., 2006) for classification, with a 97% identity threshold.

The second method, used in our study, involved treating the fecal samples with lysozyme, achromopeptidase, and proteinase K for lysis, followed by phenol-chloroform separation and ethanol precipitation. The DNA, extracted by this enzymatic lysis method, was preserved at −80°C prior to 16S rRNA sequencing. The resulting sequences were grouped into OTUs using UCLUST, also with a 97% identity threshold.

### 2.8 Post-GWAS analyses

#### 2.8.1 Comparison with previous host–microbiome association studies

We conducted a literature search on PubMed to identify relevant and recent host–microbiome association studies across multiple populations. We found four studies from 2020 to date that focused on Japanese, European, and multi-ancestry populations (Ishida et al., 2020; Kurilshikov et al., 2021; Lopera-Maya et al., 2022; Qin et al., 2022). From these studies, we obtained their summary statistics and extracted the significant associations at the phylum, genus, and species levels to compare with our results. Additionally, we directly compared the core genus relative abundance from Ishida et al. (2020) with the genus relative abundance in our sample and calculated the correlation coefficient (R) between the two groups.

### 2.9 Association analyses between high-impact annotated variants and microbiome data

#### 2.9.1 Variant annotation of host genotype whole-genome sequencing data

For variant annotation, we utilized the QCed Plink bfiles, which were converted to VCF format files using Plink v1.9 (Purcell et al., 2007). Once we obtained the VCF files, we applied filters to exclude indel variants larger than 10 base pairs, indels with a quality score below 20, and single nucleotide variants with a quality score below 30. Next, we annotated the variants using SNPeff software (Cingolani et al., 2012) and extracted the high-impact variants (10,502) by applying a SnpSift filter. Finally, the selected high-impact variants were converted back to Plink bfile format and extracted the variants with a minor allele frequency (MAF) of at least 0.05 for further downstream analyses.

#### 2.9.2 Phenome-wide association analysis

From the high-impact-annotated variants, we conducted PheWAS analysis on all phyla, class, order, family, genera, species, and bacterial pathways. This analysis was performed using the R PheWAS package (Carroll et al., 2014), with the phenotypes (all 227 taxonomic levels and bacterial pathways) as dependent variables and the variants (1,412 high-impact variants with MAF≥0.05) as independent variables. We also considered age, sex, sequencing batch (W36 or W37), and clinical group (normal, obese, or prediabetic) as covariates. Associations were considered significant if they passed the *p*-value threshold after multiple testing correction. The *p*-value threshold was calculated by dividing the nominal *p*-value of 0.05 by the number of tests performed (*p* < 1.56×10^−7^).

#### 2.9.3 Gene-based analysis of high-impact variants

To further analyze the high-impact variants, we mapped them to their respective genes using the gene locations from the MSigDB database (Subramanian et al., 2005). This database contains the chromosomal location of each gene. We annotated the variants into genes using the MAGMA software (de Leeuw et al., 2015) annotate function. Next, we used the variants bfile along with the annotated gene file to find associations with all taxa and bacterial pathways. This analysis was performed using MAGMA, with the phenotypes (227 taxa and bacterial pathways) as dependent variables and the genes (96 genes) as independent variables. We also considered age, sex, sequencing batch (W36 or W37), and clinical group (normal, obese, or prediabetic) as covariates. Associations were considered significant if they passed the *p*-value threshold after multiple testing correction. The *p*-value threshold was calculated by dividing the nominal *p*-value of 0.05 by the number of tests performed (*p* < 2.29×10^−6^).

### 2.10 rs671 stratified causal mediation analysis of fecal carbohydrates effects on insulin resistance

We extracted rs671 variant (MAF 27%) from 275 individuals in our dataset. These individuals were categorized into two groups based on rs671 genotypes: major allele homozygous group (GG); and minor allele homozygous and heterozygous group (AG + AA). Additionally, we randomly selected 999 SNPs with allele frequencies ranging from 25% to 30% from our dataset and categorized them into two groups: major group (major allele homozygous group); and minor group (minor allele homozygous & heterozygous group), following the same approach. The fecal metabolite data was obtained from a previous study (Takeuchi et al., 2021; Takeuchi et al., 2023), and normalized using Blom normalization method, whereas cytokine and clinical data such as Homeostatic Model Assessment of Insulin Resistance (HOMA-IR) and BMI were normalized with inverse rank-based method.

Given the evidence that proinflammatory cytokines play a key role in modulating insulin signaling, our previous research identified 29 triangular relationships in which certain cytokines significantly affected IR markers, including HOMA-IR (Takeuchi et al., 2023). To investigate whether rs671 further shapes these cytokine–IR associations, we conducted causal mediation analyses for each of the 29 relationships using 1,000 SNPs (including rs671) employing R mediation package. For each SNP, we performed 2,000 mediation analyses (1,000 SNPs × 2 groups) per relationship (2,000 × 29 = 58,000 in total) across major and minor genotype groups, then calculated Z-scores based on the p-values for the Average Causal Mediation Effect (ACME), Average Direct Effect (ADE), and Total Effect (TE). With ACME measuring the portion of the total effect of a SNP on insulin resistance, that is mediated through cytokine levels, thus quantifying the indirect effect. ADE representing the portion of the total effect that is not mediated, reflecting the direct effect on insulin resistance, and TE the sum of ACME and ADE, representing the overall effect, as schematically represented in **Supplementary Figure 2**. Because of occasional zero *p*-values, we added 0.001 before Z-transformation. We assessed the impact of rs671 by subtracting the Z-score of the minor group from that of the major group and evaluating significance.

Lastly, the rs671 association results were extracted from our GWAS analysis. Keeping only nominal associations between rs671 and microbial features, with beta values indicating the direction and magnitude of the associations.

## 3 Results

### 3.1 Selection of quantitative and binary phenotypes through normality assessment of taxa distributions

To investigate the relationship between the host and gut microbiome in the Japanese population, we enrolled 306 individuals. Whole-genome sequencing (WGS) and fecal shotgun metagenome sequencing analyses were performed on these individuals (Materials and methods). After conducting per-sample and per-marker quality control for WGS, we obtained 296 samples and approximately 13 million variants (**Supplementary Figure 3**). Additionally, we applied filtering based on prevalence and abundance, as well as transformation of the taxonomic compositional data (Materials and methods). This resulted in the identification of three phyla, six classes, seven orders, 10 families, 17 genera, 86 species and 98 bacterial pathways from the shotgun metagenome sequencing data (**Supplementary Table 1**).

Despite the filtering and inverse normal transformation (Materials and methods), several taxa, particularly at the species level, did not exhibit a normal distribution (**Supplementary File 1**). Instead, these taxa displayed a binary-like distribution, likely due to their low abundance in only a subset of individuals, which reflects the compositional nature of the microbiome at different taxonomic levels. Thus, by applying the Shapiro–Wilk test to the 227 taxa and bacterial pathways, we revealed that 140 taxa had W-statistics ≥ 0.95, indicating that their distributions approximated normality (**Supplementary Table 2**). These taxa were thus suitable for analysis as quantitative phenotypes using linear regression models in GWAS. Conversely, 87 taxa had W-statistics below 0.95, failing to meet the normality criterion. These taxa displayed distribution patterns consistent with binary traits, warranting additional analysis through logistic regression models. The histograms of taxa distributions corroborated the Shapiro–Wilk test results, as taxa failing the normality threshold often exhibited skewed or bimodal distributions. This distinction ensured that each taxon was analyzed using the most appropriate statistical model, enhancing the reliability of the GWAS findings.

### 3.2 Finding host variants associated with gut microbiome relative abundance

**Figure 1:**
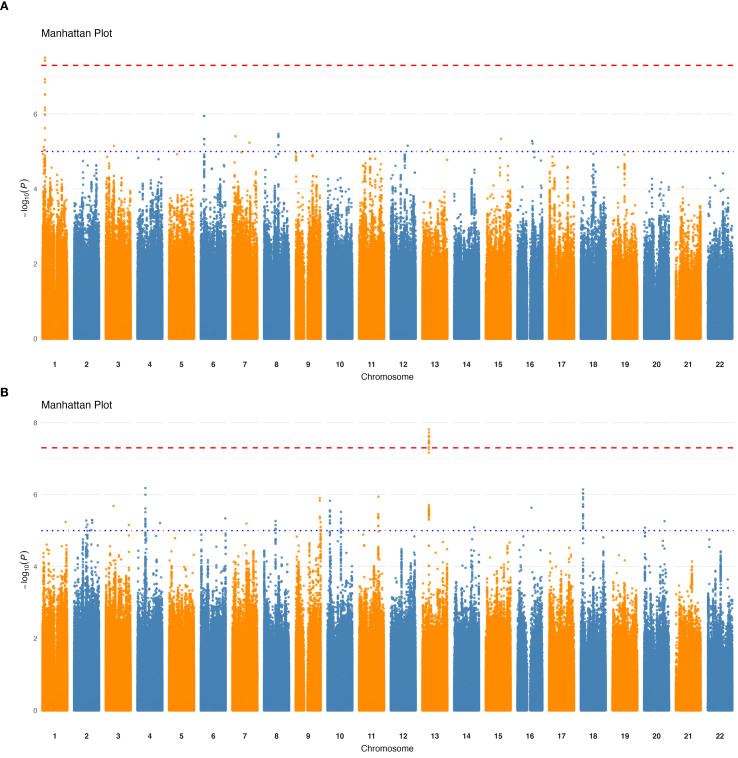
Genome-wide association results for taxa relative abundance. Manhattan plots showing significant loci (*p* < 5×10⁻^8^) associated with variations in taxa relative abundance. **(A)** Significant locus associated with the *Lachnospiraceae* family. **(B)** Significant locus associated with the genus *Dorea*. The x-axis represents genomic positions across chromosomes, and the y-axis shows the -log10 (*p*-value) of the associations. The red horizontal line indicates the genome-wide significance threshold.

Next, we conducted GWAS between the QCed host genotypes and each filtered and transformed taxa and bacterial pathway (Materials and methods). GWAS results (**Table 1**) revealed no significant associations at the phylum, class, order and species levels. However, we identified one significant (*p* < 5×10^−8^, genome-wide significance threshold) loci at the family level (**Figure 1A**), one at the genus level (**Figure 1B**), and eight significant loci at the bacterial pathway level (**Supplementary Figure 4**). Nonetheless, when applying a more stringent threshold based on the number of tested phenotypes (*p* < 2.75×10^-11^), all loci lost its significance.

**Table 1:** Summary of genome-wide association results.

Taxonomic level	Taxa	Chromosome	Position	SNP	Closest gene	REF	ALT	ALT Frq	BETA	SE	P
Family	*Lachnospiraceae*	1	24665774	rs35373536	*GRHL3*	T	G	0.482	0.484	0.085	3.12E-08
Genus	*Dorea*	13	43746561	rs9315997	*ENOX1*	G	A	0.168	-0.611	0.105	1.53E-08
Species (Binary)	*unknown_Clostridiales_[meta_mOTU_v2_5805]* (Presence)	9	36868771	rs4880022	*PAX5*	T	C	0.192	4.905 (OR)	0.288	3.25E-08
Pathway	Glycolysis / Gluconeogenesis [PATH:ko00010]	11	71053420	rs76426076	*DHCR7*	A	C	0.121	-0.687	0.117	1.42E-08
Pathway	Cytoskeleton proteins [BR:ko04812]	8	3337154	rs35061608	*CSMD1*	C	G	0.323	-0.533	0.091	1.53E-08
Pathway	Translation factors [BR:ko03012]	8	62967803	rs141994018	*NKAIN3*	A	AT	0.104	-0.831	0.143	1.59E-08
Pathway	One carbon pool by folate [PATH:ko00670]	8	62978029	rs62508547	*NKAIN3*	G	A	0.104	-0.818	0.141	1.80E-08
Pathway	DNA replication proteins [BR:ko03032]	8	13525311	rs10503463	*DLC1*	C	G	0.070	0.892	0.157	3.53E-08
Pathway	Membrane trafficking [BR:ko04131]	8	128228863	rs10087719	*POU5F1B*	A	G	0.186	-0.622	0.110	3.78E-08
Pathway	ABC transporters [PATH:ko02010]	17	2132324	rs143499	*SMG6*	C	T	0.238	-0.525	0.093	4.77E-08
Pathway	Prokaryotic defense system [BR:ko02048]	2	170700579	rs6433151	*UBR3*	C	T	0.426	-0.508	0.090	4.83E-08

Lead variants with significant associations (*p* < 5×10^−8^) are shown. Columns include the reference allele (REF), alternative allele (ALT), alternative allele frequency (ALT Frq), standard error (SE), and *p*-value (P).

**Figure 2:**
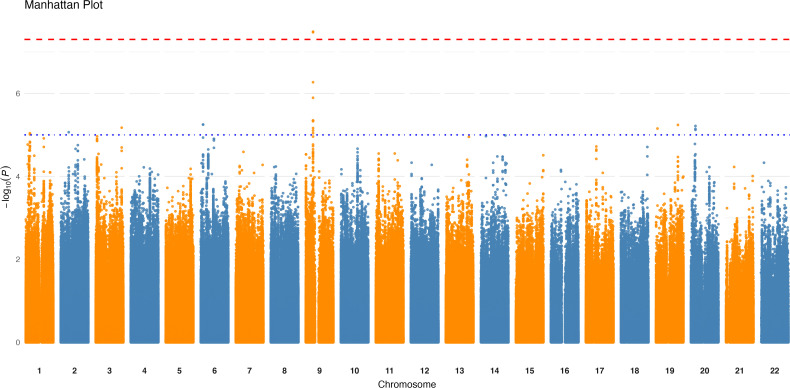
Genome-wide association results for the presence of unknown *Clostridiales* species. Manhattan plot displaying significant loci (*p* < 5×10⁻^8^) associated with the presence of the unknown *Clostridiales* species [meta_mOTU_v2_5805]. The x-axis represents genomic positions across chromosomes, and the y-axis shows the -log10 (*p*-value) of the associations. The red horizontal line indicates the genome-wide significance threshold.

The logistic regression GWAS conducted on the 87 binary taxa and bacterial pathways identified one intronic variant, rs4880022-C, located in the *PAX5* gene, associated with the presence of the species unknown *Clostridiales* [meta_mOTU_v2_5805] at a p-value of 3.25×10^−8^ (**Table 1**, **Figure 2**). This association suggests that individuals carrying rs4880022-C have a higher likelihood of harboring this specific *Clostridiales* species (OR = 4.9, SE = 0.29). Additionally, several suggestive associations (*p* ≤ 1.0×10^−5^) were identified (**Supplementary Table 3**). For example, a variant in *unknown Clostridiales* [meta_mOTU_v2_6852] on chromosome 19 (19:12646825:C:CA) showed a suggestive association with an OR of 0.236 (SE = 0.27, *p* = 9.79×10^−8^), while another variant in *Clostridium innocuum* [ref_mOTU_v2_0643] on chromosome 3 (3:16735689:G:T) was associated with an OR of 5.27 (SE = 0.32, *p* = 2.44×10^−7^). These suggestive associations may represent potential genetic influences on the presence of other low-abundance taxa, specially from the *Clostridiales* family, and warrant further investigation. However, after applying the stringent correction for multiple testing (*p* ≤ 9.32×10^−11^), the association between rs4880022 and unknown *Clostridiales* [meta_mOTU_v2_5805] did not remain statistically significant.

### 3.3 Lack of reproducibility of results from previous host–microbiome GWAS

We compared our results with the latest four studies on host–microbiome GWAS in Japanese (Ishida et al., 2020), European (Lopera-Maya et al., 2022; Qin et al., 2022), and multi-ancestry populations (Kurilshikov et al., 2021) (**Table 2**). In our study, we analyzed 227 taxa and pathways from 296 individuals and found 11 independent significant loci. However, as observed in previous comparable studies, there was a lack of reproducibility in significant GWAS associations across different studies (**Supplementary Table 4**), with a direction of effect consistent in only 46% of the associations, indicating that the observed associations may be due to random chance rather than true genetic effects. When considering the association analysis conducted in Japanese ethnicity by Ishida et al (Ishida et al., 2020), there was a lack of replicated significant associations between studies, with only four out of 38 associations being nominally significant in our results (**Supplementary Table 4**). Additionally, the direction of effect for the significant associations between studies was inconsistent when compared to our summary statistics, with only 53% showing the same direction of effect. Overall, this inconsistency was demonstrated by a weak positive linear Pearson correlation and a *p*-value indicating weak correlation of 0.334 (*p* = 0.020) when comparing with beta values from all significant variants from previous studies. Moreover, a Pearson correlation of 0.374 (*p* = 0.021) was observed when comparing against Ishida et al. (2020) effect sizes from their suggested associations (**Supplementary Figure 5**).

**Table 2:** Comparison of host–microbiome GWAS results across studies.

Reference	Sample size	Cohort	Population	Number of taxa	Number of variants	Genotyping approach	Significance threshold	Number of taxa with significant associations	Model for GWAS	Software for GWAS	Covariates for GWAS
Our study	296	single	Japanese (Tokyo University Hospital)	227	12985047	Whole-genome sequencing	5.0.E-08	11	Linear regression model	PLINK2	sex, age, sequencing batch, clinical group, 10PCs
Qin et al. (2022), *Nat. Genet*.	5959	single	Finnish (FINRISK02)	2801	7967866	Genotyping array + imputation	5.0.E-08	471	Linear mixed model	BOLT_LMMv2.3.2	age, sex, genotyping batch, 10PCs
Lopera-Maya et al. (2022), *Nat. Genet*.	7738	single	Dutch (Dutch Microbiome Project)	207	5584686	Genotyping array + imputation	5.0.E-08	37	Linear mixed model	SAIGE v.0.38	age, sex, GRM among participants
Kurilshikov et al. (2021), *Nat. Genet*.	18340	multiple	24 cohorts	385	NA	Genotyping array + imputation	5.0.E-08	27	Linear regression model	In-house eQTL	age, sex, 10PCs
Ishida et al. (2020), *Com. Bio.*	1068	single	Japanese (MYCODE)	21	558583	Genotyping array	1.00E-5 (suggested)	20	Linear regression model	Plink1.9	138 demographic variables, sex, age, 2PCs

Summary of findings from our study compared with four recent host–microbiome GWAS conducted in Japanese, European, and multi-ancestry populations. The table includes the number of significant loci, taxa analyzed, and reproducibility metrics.

### 3.4 Discrepancy in relative abundance correlation across two Japanese population cohort studies

**Figure 3:**
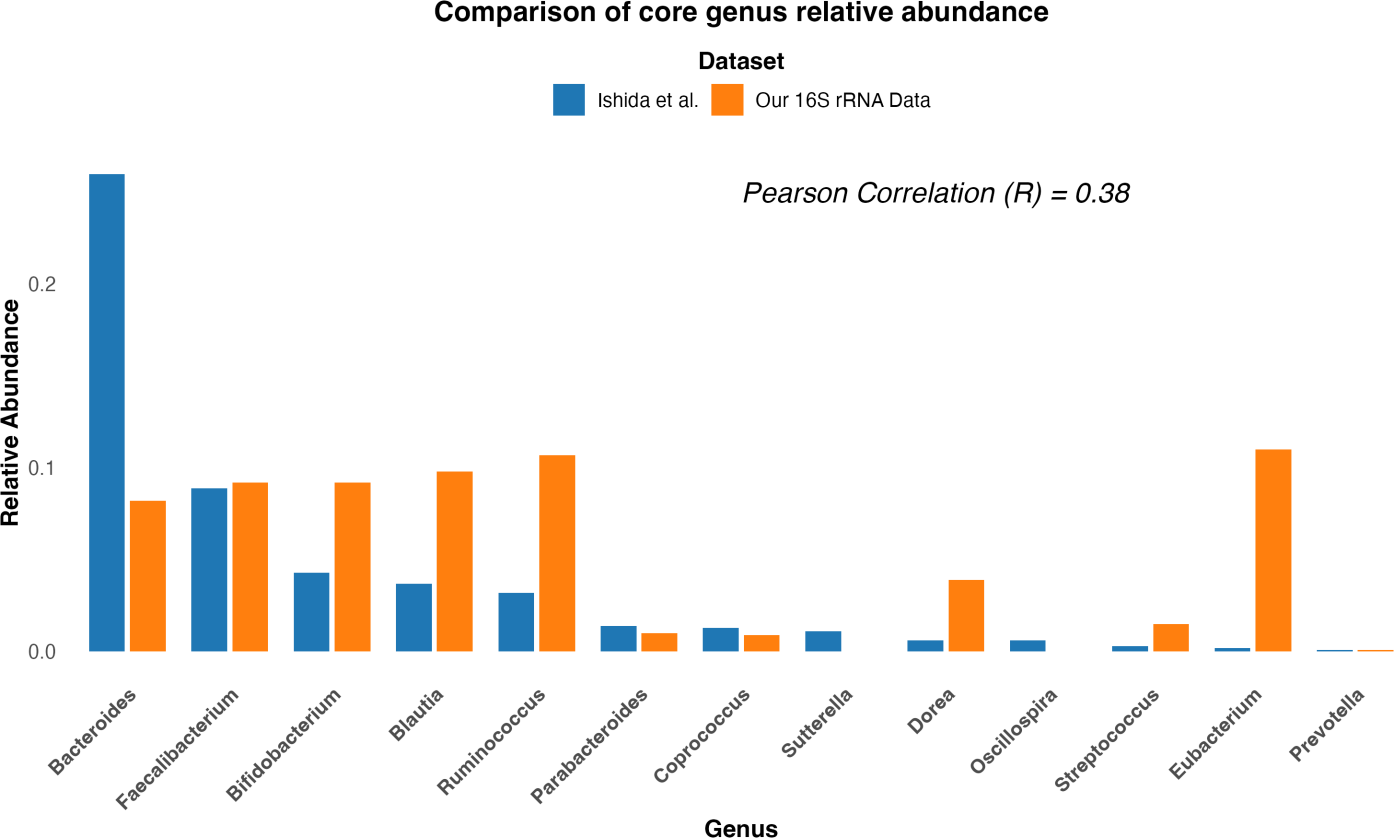
Comparison of core genus relative abundance composition between two Japanese cohort studies. Bar plot comparing the core genus relative abundance composition between the study by Ishida et al. (2020) (blue) and our study (orange). The x-axis represents bacterial genera, and the y-axis shows the relative abundance. The comparison highlights differences in the relative abundance of key genera between the two studies.

To further investigate the lack of reproducibility between studies, particularly those conducted on the same ethnicity (Japanese), we compared the raw core genus relative abundance composition from the study by Ishida et al. with the same genera’s relative abundance obtained from our 16S rRNA sequencing (Materials and methods). As shown in **Figure 3**, the comparison revealed a low correlation (R = 0.38, *p* = 0.19) and a Bray–Curtis dissimilarity value of 0.46, implying a moderate dissimilarity between the two cohorts. This suggests that while the two cohorts share some common genera, they differ in their relative abundance composition.

**Figure 4:**
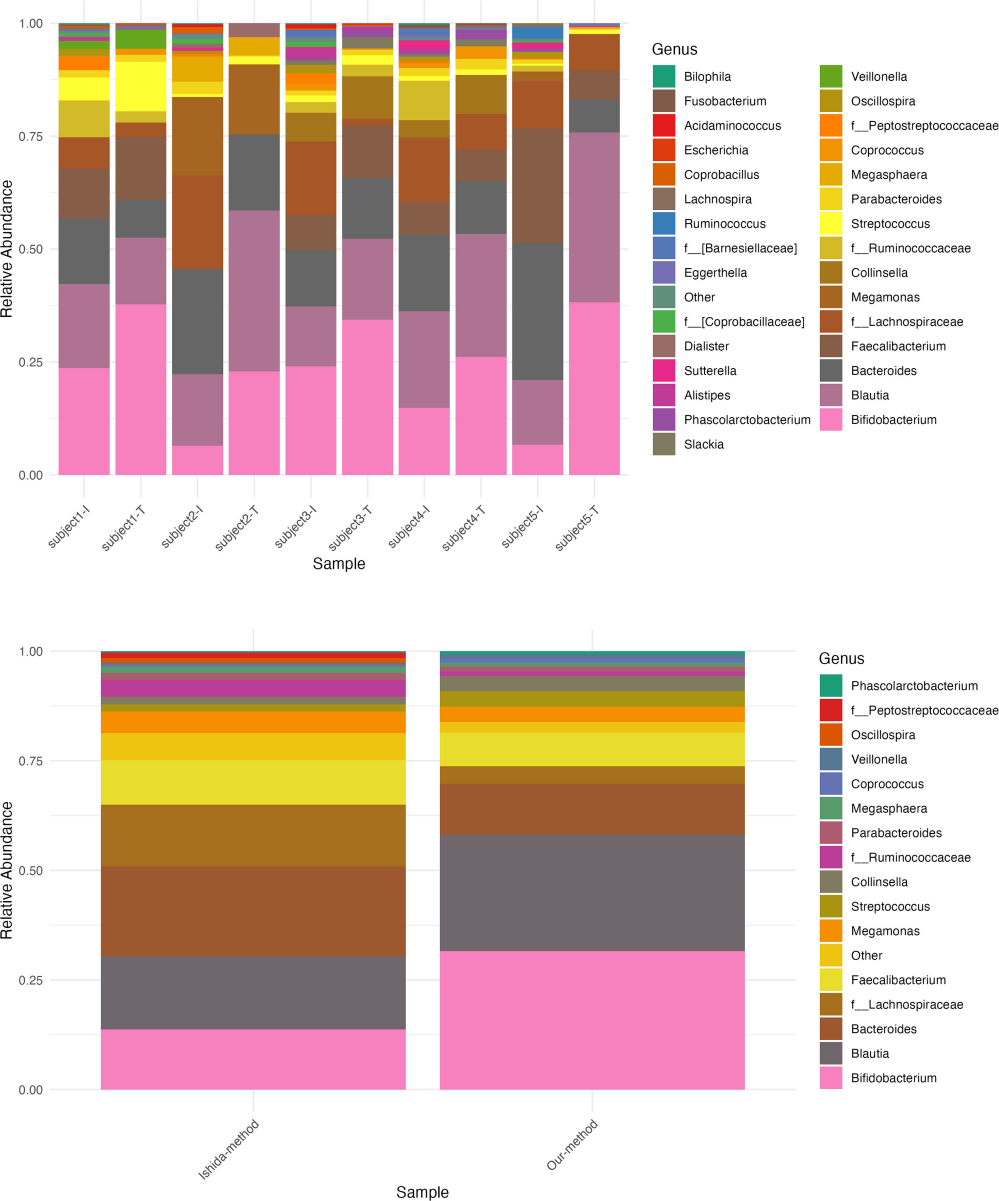
Relative abundance composition of bacterial genera using two different methods. Comparison of bacterial genus-level relative abundance in five samples processed using the Ishida et al. method and our method. Top panel: Stacked bar chart showing the relative abundance of bacterial genera in each sample. Sample IDs are shown on the x-axis, with “-I” indicating results from the Ishida et al. method and “-T” indicating results from our method. The y-axis represents the relative abundance, with each color corresponding to a different bacterial genus. Bottom panel: Stacked bar chart showing the average relative abundance of bacterial genera across the five samples for each method. The x-axis represents the method used, and the y-axis shows the average relative abundance.

In an attempt to understand the possible causes of these differences in relative abundances, we replicated the experimental workflow used in both studies to generate the 16S rRNA sequencing relative abundance data (**Table 3**). We applied the same conditions and steps from both methods to five healthy Japanese samples (Materials and methods) and generated the relative abundance composition for each sample at the phylum and genus levels. First, we compared the replicates’ relative abundance at the genus level and found an average correlation between replicates of 97.4% when using the Ishida et al. method and 99.4% when performing our method (**Supplementary Figure 6**). Then, when comparing between methods, our results showed an average correlation of 95.5% (*p* = 2.2 × 10⁻^4^) at the phylum level. **Figure 4** illustrates a more straightforward comparison of relative abundance data, by extracting only the core genera from the method used by Ishida et al. and compared it with the relative abundance output from the method used in our study. Similar to our initial comparison of raw genus relative abundance composition depicted in **Figure 3**, our analysis showed a higher relative abundance of *Bacteroides* and a decreased relative abundance of *Bifidobacterium* in the output from the Ishida et al. method. With an overall average correlation of core genus between the two methods of 77% (*p* = 4.12 ×10^−6^).

**Table 3:** Comparison of experimental workflows for 16S rRNA sequencing.

-	Ishida et al.	Our study
Number of samples	1098	306
Individual characteristics	Healthy Japanese	100 Healthy, 100 Obese, 100 IGT Japanese
Sample collection kit	Techno Suruga Laboratory Co., Ltd., Shizuoka, Japan	NA
Preservation method	GuSCN solution	Stored −80 C
DNA extraction from feces	Bead-beating method	Enzymatic cell lysis
16S rRNA region sequenced	V3–V4 region	V1–V2 region
PCR	TaKaRa Ex Taq HS Kit. Barcoded PCR	PCR with barcoded primers
PCR amplicon purification	QIAquick PCR Purification Kit (Qiagen, Valencia, CA, United States)	AMPure XP magnetic purification beads (Beckman Coulter, Inc.)
Sequencing	Illumina MiSeq	Illumina MiSeq
QC quality score	Quality score of less than 25 was trimmed	Quality score of less than 25 was trimmed
QC chimeric sequences	Reference-based chimaera checking in USEARCH (ver. 5.2.32) and the Genomes OnLine Database (GOLD)	Reads having BLAST match lengths <90% with the representative sequence in the 16S databases were considered as chimeras
16S database used	Greengenes reference database	RDP v. 10.27, CORE (http://microbiome.osu.edu/), and NCBI FTP site (ftp://ftp.ncbi.nih.gov/genbank/, December 2011)
Genus level OTU	Analysed by QIIME v 1.8.0. OTUs assigned by open-reference OTU picking with a 97% pairwise identity	3,000 reads/sample chosen. All read sorted and grouped into OTUs using UCLUST (http://www.drive5.com/) with a identity threshold of 97%

Detailed comparison of the experimental workflows used in the Ishida et al. study and our study for generating genus-level relative abundance data.

### 3.5 Phenome-wide analysis of high-impact variants shows novel host functional genetic variant associated with gut microbiome composition

To overcome our limited sample size and find further associations between common host functional genetic variants and our taxonomic compositional data, we conducted a PheWAS across all taxonomic levels. We analyzed 1,412 high-impact variants, which likely cause a disruption in gene function, including nonsense mutations, frameshift mutations, and splice site mutations, with a minor MAF ≥ 0.05 (Methods, **Supplementary Table 5**). This approach allowed us to assess the impact of common, functional genetic variants on the relative abundance of gut microbial taxa.

**Figure 5:**
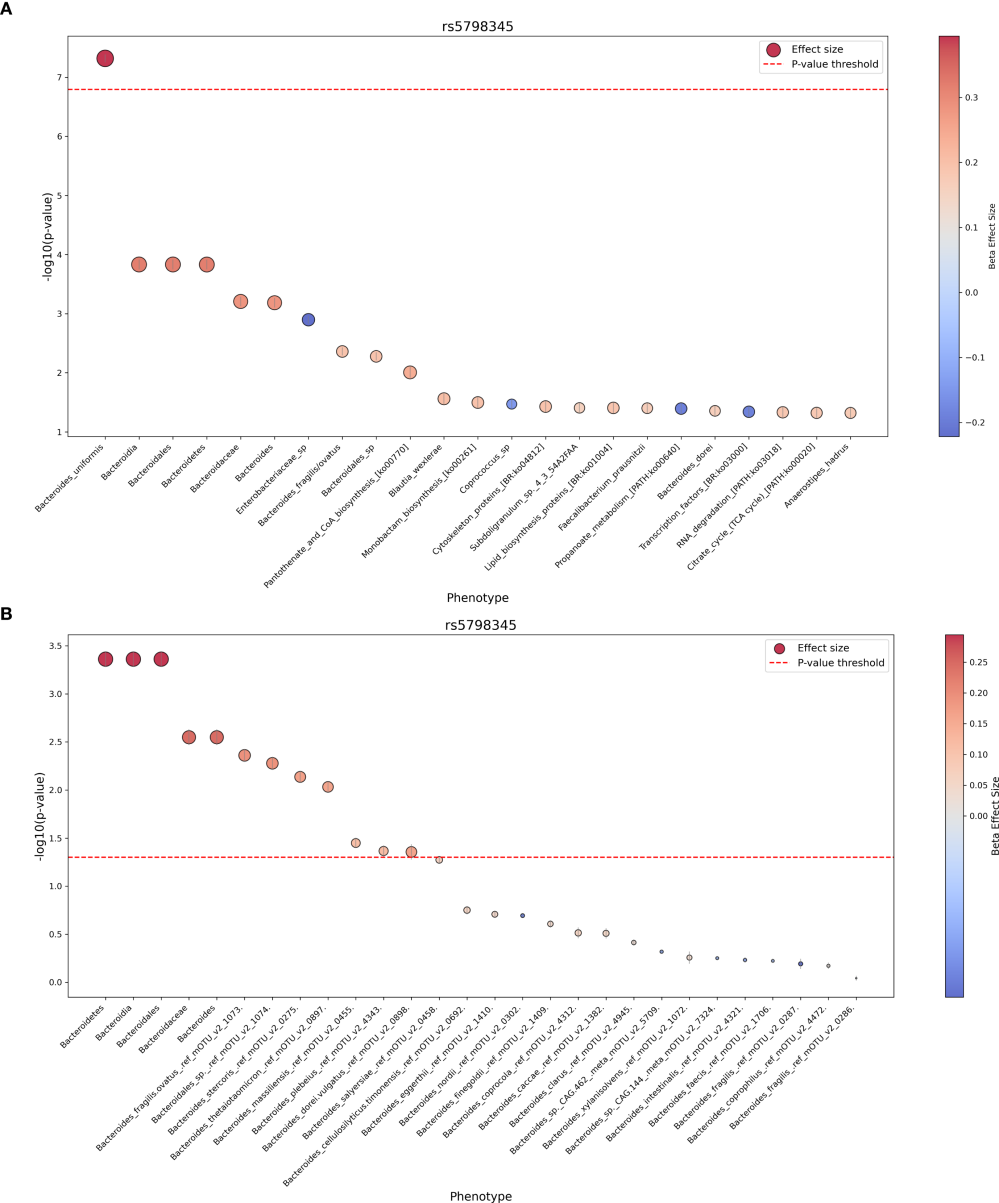
Phenome-wide association results for the *OR6C1* frameshift variant rs5798345-CA. Results from the PheWAS analysis of variant rs5798345-CA, showing associations with gut microbiome taxa and pathways. **(A)** Nominally significant associations with taxa and pathways. Node size represents the effect size (beta coefficient), and node color indicates the direction of the effect (positive or negative). **(B)** Results excluding *Bacteroides uniformis* and including additional Bacteroides species.

Our PheWAS identified one significant frameshift variant located in the *OR6C1* gene (rs5798345-CA, c.24dup, p.Glu9ArgfsTer10) associated with the relative abundance of *Bacteroides uniformis* (beta = 0.394, *p* ≤ 4.78 × 10⁻^8^; **Table 4**). The positive beta value indicates that individuals carrying the frameshift variant have a higher relative abundance of *B. uniformis* in their gut microbiome. The same frameshift variant demonstrated nominal significance in other *Bacteroides* species (*Bacteroides fragilis, Bacteroides dorei, Bacteroidales_sp*) and their corresponding taxonomic levels, all showing a consistent positive direction of effect (**Figure 5A**). Furthermore, we excluded *Bacteroides uniformis* and included other available *Bacteroides* species from our cohort that had been filtered out due to low prevalence and abundance. We then re-ran the PheWAS analysis using only rs5798345 to determine whether the higher taxonomic levels of *B. uniformis* retained their nominal significance, as well as to identify any additional *Bacteroides* species with nominal significance and consistent direction of effects. Notably, the higher taxonomic levels retained their nominal significance, and we identified four additional nominally significant independent species (*B. stercoris*, *B. thetaiotaomicron*, *B. massiliensis*, and *B. plebeius*) with concordant directions of effect (**Figure 5B**; **Supplementary Table 6**). These findings suggest that the *OR6C1* variant may have a broader impact on the abundance of taxa within the *Bacteroides* genus.

**Table 4:** Phenome-wide association results for high-impact variants.

Class	Associated taxa	SNP	Gene	ALT Frq	Beta	SE	P	Type of variant	HGVS coding	HGVS protein
Phylum	*Actinobacteria*	rs12139100	*PLA2G2C*	0.088	0.589	0.147	7.64.E-05	Nonsense	c.97C>T	p.(Arg33Ter)
**Species**	***Bacteroides_uniformis_[ref_mOTU_v2_0899]***	**rs5798345**	***OR6C1***	**0.360**	**0.394**	**0.070**	**4.78.E-08**	**Frameshift**	**c.24dup**	**p.(Glu9ArgfsTer10)**
Species	*Coprococcus:[ref_mOTU_v2_4313]*	rs201931080	*PARPBP*	0.050	0.727	0.152	2.81.E-06	Frameshift	c.377_378del	p.(Thr126SerfsTer4)
Species	*Dorea_longicatena_[ref_mOTU_v2_4203]*	rs6760610	*CCDC148*	0.412	0.306	0.070	1.55.E-05	Splice Acceptor	c.1252-15016C>T	
Species	*Ruminococcus_torques_[ref_mOTU_v2_4718]*	rs34358	*ANKDD1B*	0.357	−0.336	0.078	2.35.E-05	Nonsense	c.1439G>A	p.(Trp480Ter)
Species	*Ruminococcus_bicirculans_[ref_mOTU_v2_2358]*	rs35706572	*WNK1*	0.180	0.346	0.083	3.88.E-05	Frameshift	c.2175dup	p.(Ile726HisfsTer45)
Species	*unknown_Eubacterium_[meta_mOTU_v2_6657]*	rs2781377	*ESR2,SYNE2*	0.136	0.449	0.109	4.98.E-05	Nonsense	c.12002G>A	p.(Trp4001Ter)
Species	*unknown_Clostridium_[meta_mOTU_v2_6792]*	rs9610445	*APOL4*	0.075	0.492	0.123	8.18.E-05	Splice Donor	c.35 + 2T>G	
Species	*Faecalibacterium_prausnitzii_[ref_mOTU_v2_4875]*	rs1138349	*PCGF2*	0.270	−0.312	0.079	9.80.E-05	Nonsense	c.435C>T	p.(Asp145=)
Species	*unknown_Clostridiales_[meta_mOTU_v2_7531]*	rs6925614	*DACT2*	0.317	0.255	0.064	9.81.E-05	Missense	c.1052A>T	p.(Glu351Val)
Pathway	Homologous recombination [PATH:ko03440]	rs1861050	*CC2D2A*	0.139	−0.511	0.124	5.02.E-05	Nonsense	c.262C>T	p.(Arg88Ter)
Pathway	Lysine biosynthesis [PATH:ko00300]	rs12139100	*PLA2G2C*	0.088	0.585	0.142	5.20.E-05	Nonsense	c.97C>T	p.(Arg33Ter)
Pathway	Ribosome [PATH:ko03010]	rs3841128	*GRIA1*	0.111	−0.545	0.134	6.54.E-05	Frameshift	c.31dup	p.(Leu11ProfsTer13)
Pathway	Transcription factors [BR:ko03000]	rs1138349	*PCGF2*	0.270	0.368	0.093	9.28.E-05	Nonsense	c.435C>T	p.(Asp145=)
Pathway	Glyoxylate and dicarboxylate metabolism [PATH:ko00630]	rs1010425	*SIGLEC10*	0.129	−0.490	0.124	9.69.E-05	Missense	c.144G>T	p.(Gln48His)
Pathway	RNA polymerase [PATH:ko03020]	rs201764113	*KRTAP4-8*	0.437	0.328	0.083	9.70.E-05	Frameshift	c.1dup	p.(Met1AsnfsTer12)

Summary of significant and nominal associations between 1,412 high-impact variants and gut microbiome taxa or pathways. Columns include the alternative allele frequency (ALT Frq), standard error (SE), and *p*-value (P). Statistically significant results are shown in bold.

### 3.6 Gene-based analysis of high impact annotated variants

To enhance the identification of loci, specifically genes, that may be linked to gut microbiome composition in our cohort, we employed the strategy of mapping our high-impact-annotated variants to their corresponding genes. Subsequently, we conducted a gene-based analysis using MAGMA software (Materials and methods). However, we were unable to find any robust functional associations (*p* < 2.29×10^−6^; **Supplementary Table 7**).

### 3.7 rs671 stratified causal mediation analysis between fecal carbohydrates, plasma cytokines, and clinical markers

According to previous studies, causal mediation analyses demonstrated that some inflammatory cytokines may mediate the effects of fecal carbohydrates on insulin resistance (Takeuchi et al., 2023). Here, we investigated whether host genetics—particularly rs671—could further shape these relationships in our cohort. This SNP, which profoundly affects alcohol metabolism, has been linked to cardiovascular diseases, cancer (Chang et al., 2017; Zhang et al., 2023; Koyanagi et al., 2024), and susceptibility to type 2 diabetes in males (Spracklen et al., 2020).

Our GWAS results indicate that the rs671 variant is nominally associated with the bacterial chemotaxis pathway [PATH:ko02030], exhibiting a positive beta value (**Supplementary Table 8**). Additionally, rs671 shows nominal associations with the relative abundance of *Clostridium innocuum* and *Streptococcus salivarius*, as well as their higher taxonomic levels. For *S. salivarius*, associations extend to the genus *Streptococcus*, family *Streptococcaceae*, order *Lactobacillales*, and class *Bacilli*. Both species are part of the phylum *Firmicutes*. The consistent positive beta values across these taxa suggest a potential relationship between the rs671 minor allele and increased abundance of these bacteria, as well as enhanced representation of bacterial chemotaxis functions within the gut microbiota, though the statistical significance of these associations were lost after multiple testing correction.

To assess the impact of rs671 on the mediation of fecal carbohydrates’ effects on insulin resistance, we calculated Z-score differences for the ACME, ADE, and TE across 29 cytokine–IR relationships for 1,000 SNPs (**Supplementary Table 9**; Materials and methods). Our results revealed one pair (ACME), six pairs (ADE), and one pair (TE) that surpassed a Z-score difference of 2, suggesting that this EAS-specific variant may influence how carbohydrates affect IR. These findings underscore the potential importance of rs671 in modulating gut microbial pathways and cytokine-mediated IR processes.

## 4 Discussion

The interplay between host genetic variation and gut microbiome composition has predominantly been investigated in European populations (Davenport et al., 2015; Scepanovic et al., 2019; Rühlemann et al., 2021; Lopera-Maya et al., 2022; Qin et al., 2022). However, the Japanese population represents a significant area of opportunity for research regarding the influence of host genetics on microbiota shaping. Recently, associations between changes in microbiome composition, such as dysbiosis, and the risk and development of various diseases, including metabolic, neurological, and autoimmune conditions, have been noted (Liu et al., 2019; Varesi et al., 2022; Amin et al., 2023). Despite this, only one study has specifically explored the interactions between host genetics and microbiome composition in the Japanese population, primarily due to other studies focusing on the interplay between microbiome composition and disease (Liu et al., 2019; Varesi et al., 2022; Amin et al., 2023), while neglecting host genetic variability.

Here, we conducted a comprehensive suite of analyses in a sample of 296 Japanese individuals, utilizing WGS and obtaining 12,985,047 quality-controlled variants. This encompassed the first mGWAS investigating the relationship between host genetic variation and the relative abundance of bacterial species and their related pathways from shotgun metagenomic sequencing in the Japanese population. We performed quantitative GWAS using linear regression for taxonomic levels that passed the Shapiro–Wilk test for normality and binary trait GWAS using logistic regression on taxa and bacterial pathways that did not exhibit normal distributions after transformation. From these analyses, we identified a total of 11 significant loci. Interestingly, we found an association between the intronic SNP rs4880022-C in the *PAX5* gene and the presence of the species unknown_Clostridiales [meta_mOTU_v2_5805]. *PAX5* encodes a transcription factor crucial for B-cell development and function (Cobaleda et al., 2007). Variations in immune-related genes like *PAX5* can influence host immune responses and potentially affect the colonization and abundance of specific gut microbes (Vicente-Dueñas et al., 2020). The *Clostridiales* order includes many bacterial species important for gut homeostasis and modulating immune responses (Zheng et al., 2020; Li et al., 2024). While the association did not reach genome-wide significance after correction, it highlights the importance of considering non-normally distributed taxa in genetic studies of the microbiome. Further research is needed to validate this association and explore the underlying mechanisms linking host genetics to microbiome prevalence.

When comparing our findings with previous GWAS (Ishida et al., 2020; Kurilshikov et al., 2021; Lopera-Maya et al., 2022; Qin et al., 2022), we were unable to replicate any of their associated variants, nor did we observe any variants in close proximity to their lead variants (**Supplementary Table 4**). This lack of replication is not entirely surprising given the known challenges in replicating gut microbiota results. Low replication for gut microbiota results is a known issue in the field. Even though we would anticipate consistent results between cohorts from the same ethnic population, such as our study and the work conducted by Ishida et al (Ishida et al., 2020), reproducibility can be elusive due to various factors, including diet, environmental influences, sample processing, and the classification pipeline used for bacterial taxa (Hosomi et al., 2017; Kawada et al., 2019; Leeming et al., 2019; Scepanovic et al., 2019; Qin et al., 2022; Vilchez-Vargas et al., 2022; Mori et al., 2023).

Our findings, when compared with the previous Japanese study by Ishida et al. (2020), revealed a low correlation (R = 0.38, Bray–Curtis dissimilarity = 0.46) between the raw core genus relative abundance composition from both studies. A comparative analysis of methodologies employed in both studies revealed significant differences, particularly in fecal preservation, DNA extraction, and post-sequencing analysis (**Table 3**). These methodological variations likely explain the observed lack of correlation at the genus level. To validate these findings, we replicated the methods used in both studies to process fecal samples from five healthy subjects for microbial analysis using 16S rRNA sequencing. The consistency of our experimental results with the low correlation between relative abundance outputs (**Figure 4**) emphasizes the influence of methodological variations on study outcomes. The impact of factors such as sample storage conditions and DNA extraction methods on gut microbiota composition has been well-documented (Hosomi et al., 2017; Kawada et al., 2019; Mori et al., 2023). For instance, the use of guanidine thiocyanate solution (GuSCN) for fecal sample storage may not be ideal if proper protocols are not followed (Hosomi et al., 2017). Similarly, the choice between mechanical and enzymatic lysis for bacterial DNA extraction can significantly impact results, particularly for the phylum Bacteroidetes and genus *Bacteroides* (Kawada et al., 2019). These methodological variations not only affect taxonomic profiles but can also influence downstream analyses, such as identifying host genetic associations with microbiome composition. Therefore, adopting standardized protocols for sample collection, preservation, DNA extraction, and sequencing is crucial. Researchers should consider the potential impacts of methodological choices and aim for consistency, especially in large-scale studies and meta-analyses. Standardizing methodologies across studies will enhance reproducibility and facilitate a more accurate understanding of the gut microbiome’s role in human health and disease.

Furthermore, by conducting PheWAS, we aimed to identify common host functional genetic variants associated with gut microbiome composition. Our analysis revealed a novel and potentially interesting association between a loss-of-function frameshift variant in the *OR6C1* gene and the relative abundance of *Bacteroides uniformis* in the gut microbiome (**Table 4**; **Figure 5a**). Specifically, individuals carrying the rs5798345-CA variant demonstrated an increased abundance of *B. uniformis* (beta = 0.394, p ≤ 4.78 × 10⁻^8^). *B. uniformis* is a prominent member of the human gut microbiota and plays a crucial role in the digestion of complex carbohydrates and modulating host immune responses (Ishikawa et al., 2013; Tufail and Schmitz, 2024). Notably, *B. uniformis* has been studied for its potential probiotic properties, including the ability to relieve symptoms of ulcerative colitis in experimental models (Zhang et al., 2024). While preliminary, the consistent positive associations observed across multiple *Bacteroides* species suggest a hypothesis that the *OR6C1* frameshift variant could have a broader influence on gut microbiome composition than initially anticipated. The *OR6C1* gene encodes an olfactory receptor belonging to the G protein-coupled receptor (GPCR) superfamily. While olfactory receptors are primarily associated with odor detection in the olfactory epithelium, emerging evidence suggests that certain olfactory receptors are expressed in other tissues (Kang and Koo, 2012; Nakanishi et al., 2023), potentially influencing physiological processes beyond olfaction. However, the specific role of OR6C1 outside the olfactory system remains largely unexplored and requires further investigation. Given that alterations in *B. uniformis* abundance have been associated with various health conditions (Yan et al., 2023), we hypothesize that the observed association between the *OR6C1* variant and *B. uniformis* abundance could potentially contribute to understanding individual differences in disease susceptibility, though additional studies are needed to validate this relationship.

To expand our search for loci associated with gut microbiome composition, we performed a gene-based analysis of the high-impact-annotated variants. However, we couldn’t find any further associations when employing this method (**Supplementary Table 7**).

For our causal mediation analysis on fecal carbohydrates, we observed that differences in the rs671 genotype likely modify the effect size of the causal relationship between glucosamine/rhamnose and host insulin resistance markers independently of cytokine mediation. Interestingly, the direction of Z-score difference of ADE (2.54) and ACME (−1.65) were opposite in the glucosamine-adiponectin-HDL combination, suggesting that rs671 genotype difference is intricately involved in these triangular relationships. Additionally, there was only one combination where the difference in ACME Z-scores exceeded 2, which was the galactose-adiponectin-HOMA-IR combination. Previous studies have reported that adiponectin is involved in biological pathways associated with HOMA-IR (Yamauchi et al., 2007; Borges et al., 2017). In a previous MR study, a potential negative association between serum adiponectin level and risk of type 2 diabetes was revealed (Nielsen et al., 2021). This finding indicates that the difference in the rs671 genotype likely influences adiponectin-mediated *in silico* relationships between fecal galactose and HOMA-IR. Given that rs671 is one of the susceptible loci for type 2 diabetes in males (Spracklen et al., 2020), this finding might help us understand the pathophysiology of type 2 diabetes.

Our exploratory GWAS analysis provides preliminary evidence of a potential association between rs671 and alterations in gut microbiota composition, particularly with *Streptococcus salivarius* and its higher taxonomic levels (**Supplementary Table 8**). *S. salivarius* is a gram-positive, facultative anaerobic bacterium that colonizes the human oral cavity and upper respiratory tract shortly after birth and is also a member of the gut microbiota (Kaci et al., 2014). Current literature indicates that *S. salivarius* produces bacteriocins that inhibit the growth of pathogenic bacteria, suggesting a protective role in the microbial ecosystem. Additionally, it exhibits anti-inflammatory properties that may influence immune responses (Kaci et al., 2014). Based on previous research, we hypothesize that changes in bacterial composition could alter the fermentation of dietary carbohydrates, affecting metabolite production and potentially influencing insulin signaling pathways (Kaci et al., 2014; MacDonald et al., 2021). Furthermore, the genus *Streptococcus* includes species that contribute to carbohydrate metabolism and produce metabolites that may impact host metabolic pathways (Thomas et al., 2011; Procházková et al., 2024). It is important to note that these observations are preliminary and did not reach genome-wide significance after multiple testing correction. To validate these nominal associations and determine whether a true biological relationship exists between rs671, *S. salivarius*, and type 2 diabetes, future studies with larger sample sizes and greater statistical power are essential.

Despite the valuable insights gained from our study, several limitations should be acknowledged to contextualize the findings appropriately. Firstly, the sample size of our cohort was relatively modest (n = 296 after quality control), which may limit the statistical power to detect genetic associations with small effect sizes. Secondly, the cross-sectional design of our study limits the ability to infer causality between host genetic variation and gut microbiome composition. While we identified associations between specific genetic variants and microbial taxa, we cannot determine the directionality of these relationships or assess temporal changes in the microbiome. Longitudinal studies are needed to establish causal links and to understand how host genetics and microbiome composition interact over time.

Future research should address these limitations through larger cohort studies and meta-analyses to enhance statistical power and findings robustness. Additionally, standardization of methodologies across microbiome studies is crucial, as variations in sample collection, DNA extraction, sequencing platforms, and bioinformatics pipelines impede reproducibility. The Microbiome Quality Control Project emphasizes this need for standardization (Sinha et al., 2017). Integration of multi-omics approaches with environmental data will provide deeper insights into host–microbiome interactions.

Overall, our comprehensive analysis has revealed significant genetic variants and functional links illuminating the complex interplay between lead variants, microbiome composition, and disease traits. By complementing GWAS with high-impact variant analyses, we addressed sample size limitations while enhancing discovery of functionally consequential variants (**Supplementary Figure 7**). Our comparative analysis findings underscore the importance of methodological consistency in microbial studies, contributing to our understanding of mechanisms driving complex diseases.

## Data Availability

Whole-genome sequencing data used in the study are available from the corresponding author upon reasonable request.
