# Supplementary material for: Interplay between host genetics and gut microbiome composition in the Japanese population
Source: Front Microbiomes. 2025 Oct 14;4:1635907. doi: 10.3389/frmbi.2025.1635907 (PMC12993688; doi:10.3389/frmbi.2025.1635907)
Supplement: Supplementary file 1 [file DataSheet1.pdf]

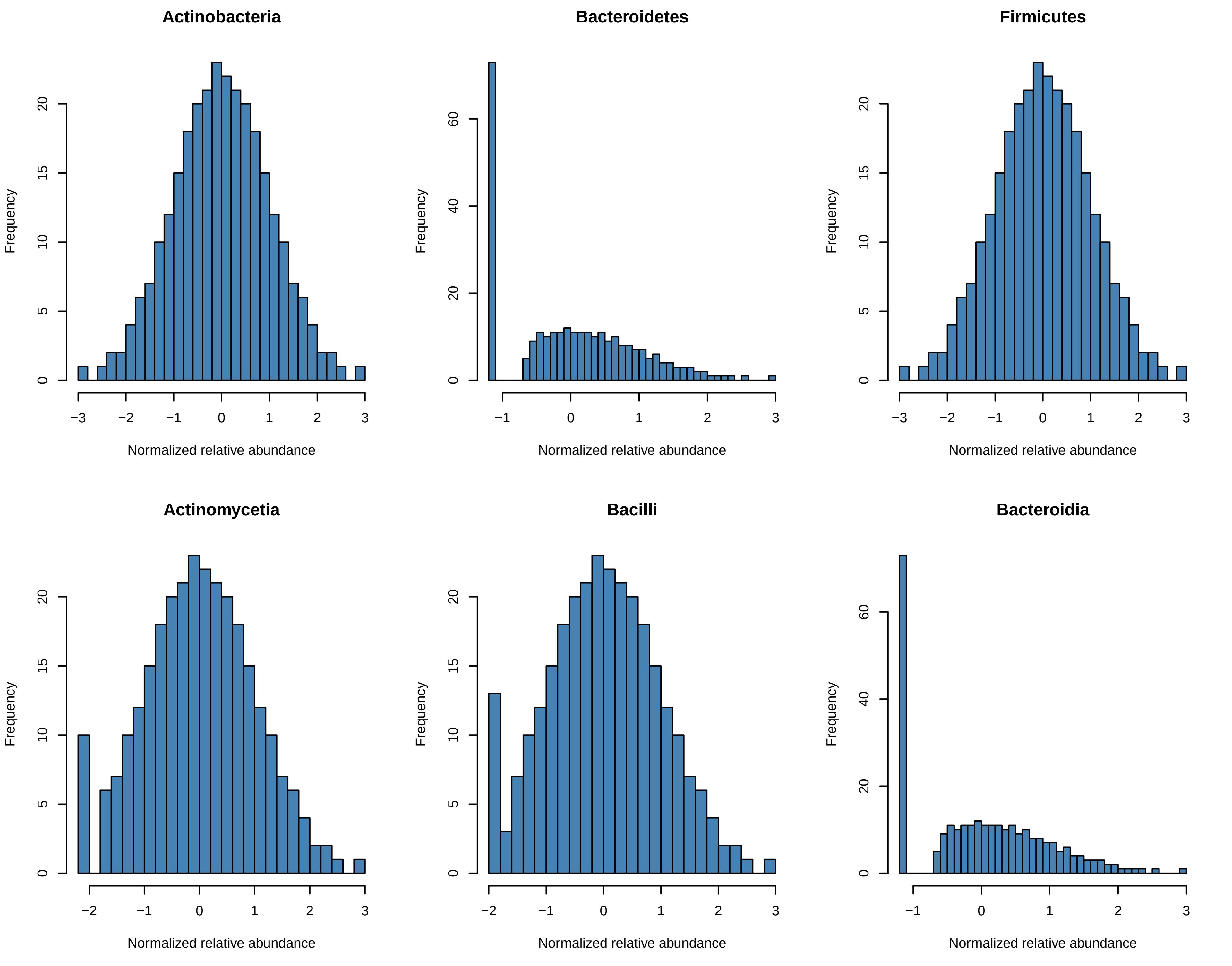
Actinobacteria
Bacteroidetes
Firmicutes
Actinomycetia
Bacilli
Bacteroidia
Frequency
Normalized relative abundance

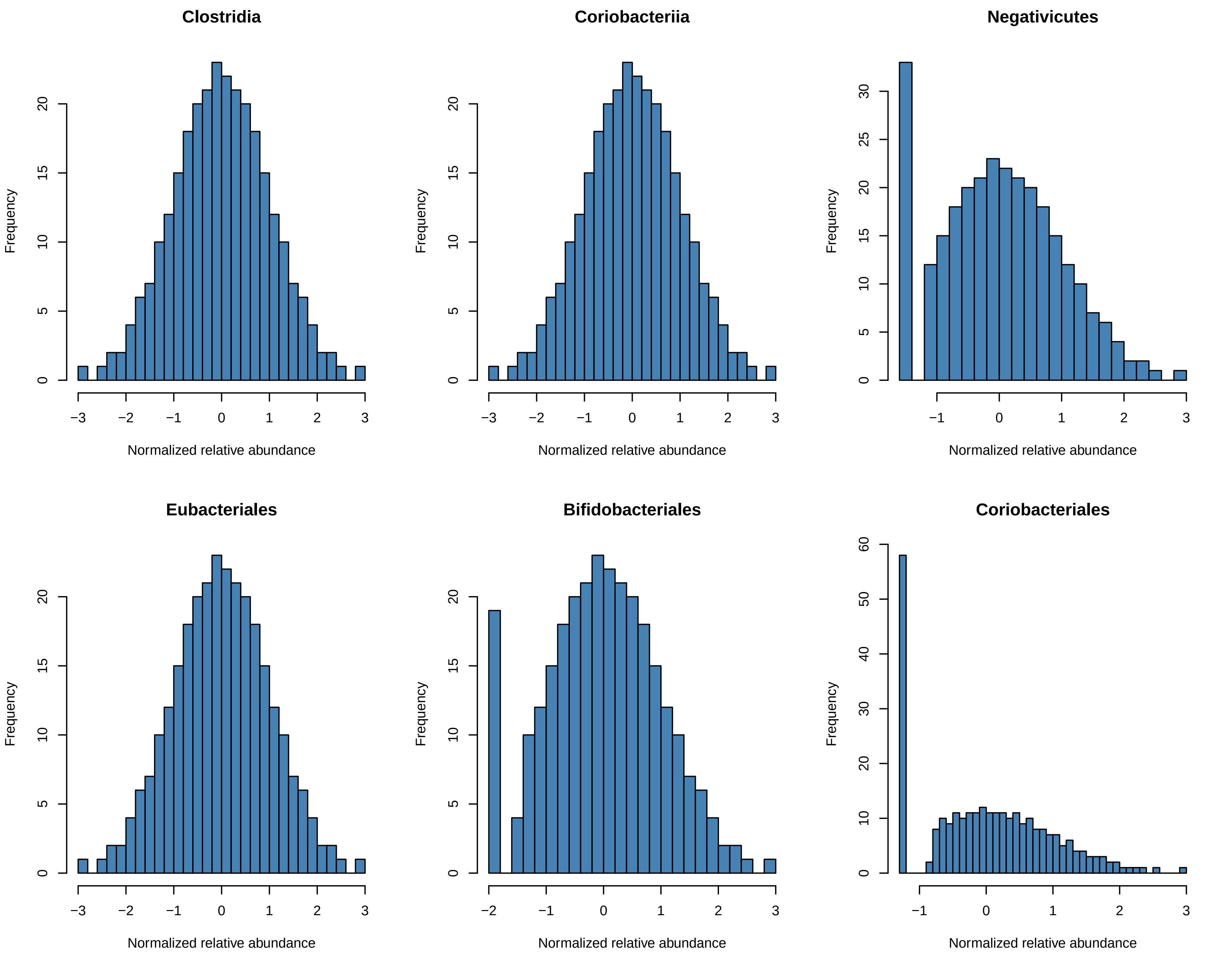
Clostridia
Coriobacteriia
Negativicutes
Eubacteriales
Bifidobacteriales
Coriobacteriales
Frequency
Normalized relative abundance

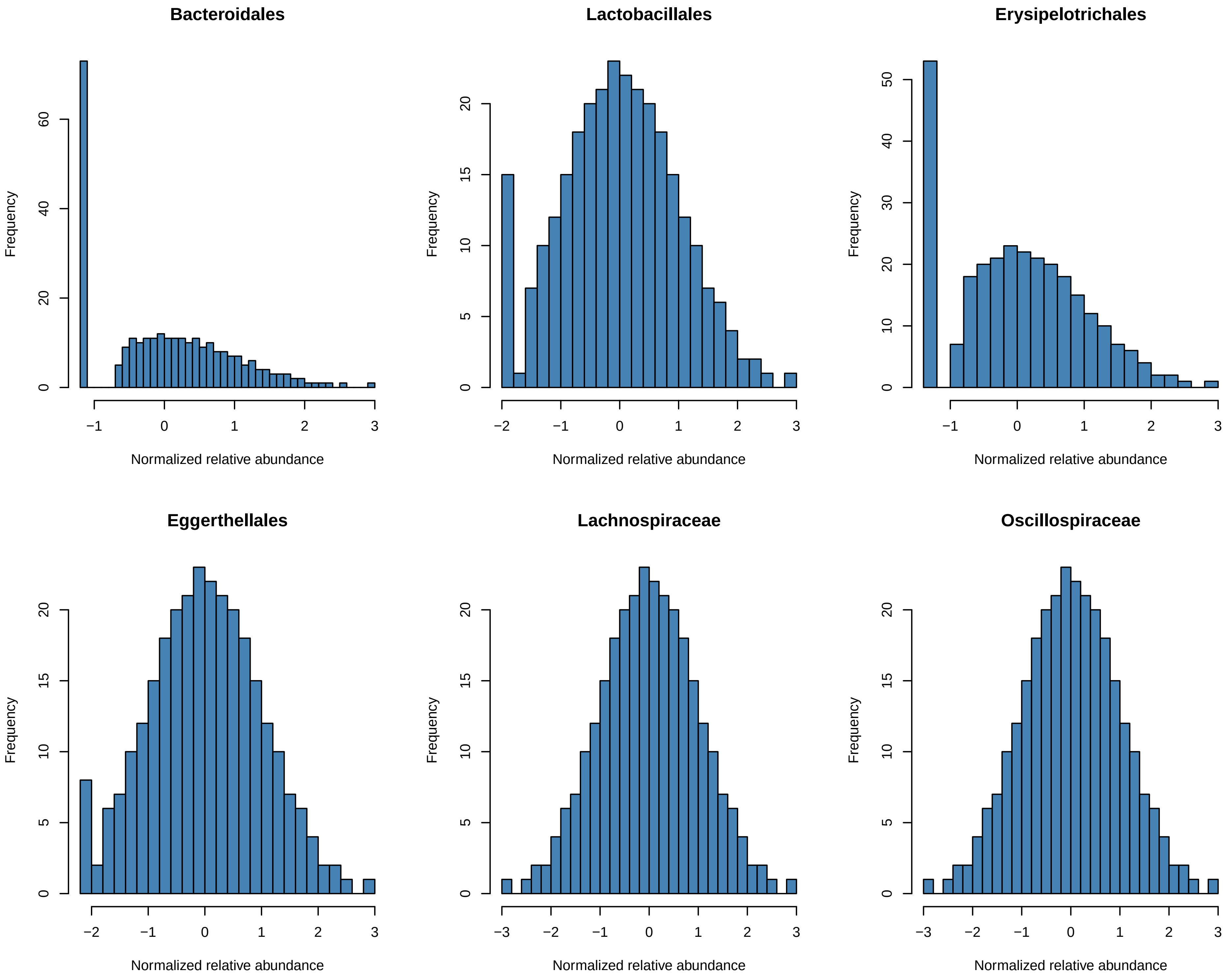
Bacteroidales
Lactobacillales
Erysipelotrichales
Eggerthellales
Lachnospiraceae
Oscillospiraceae
Frequency
Normalized relative abundance
0
20
40
60
5
10
15
20
30
50
−3
−2
−1
1
2
3

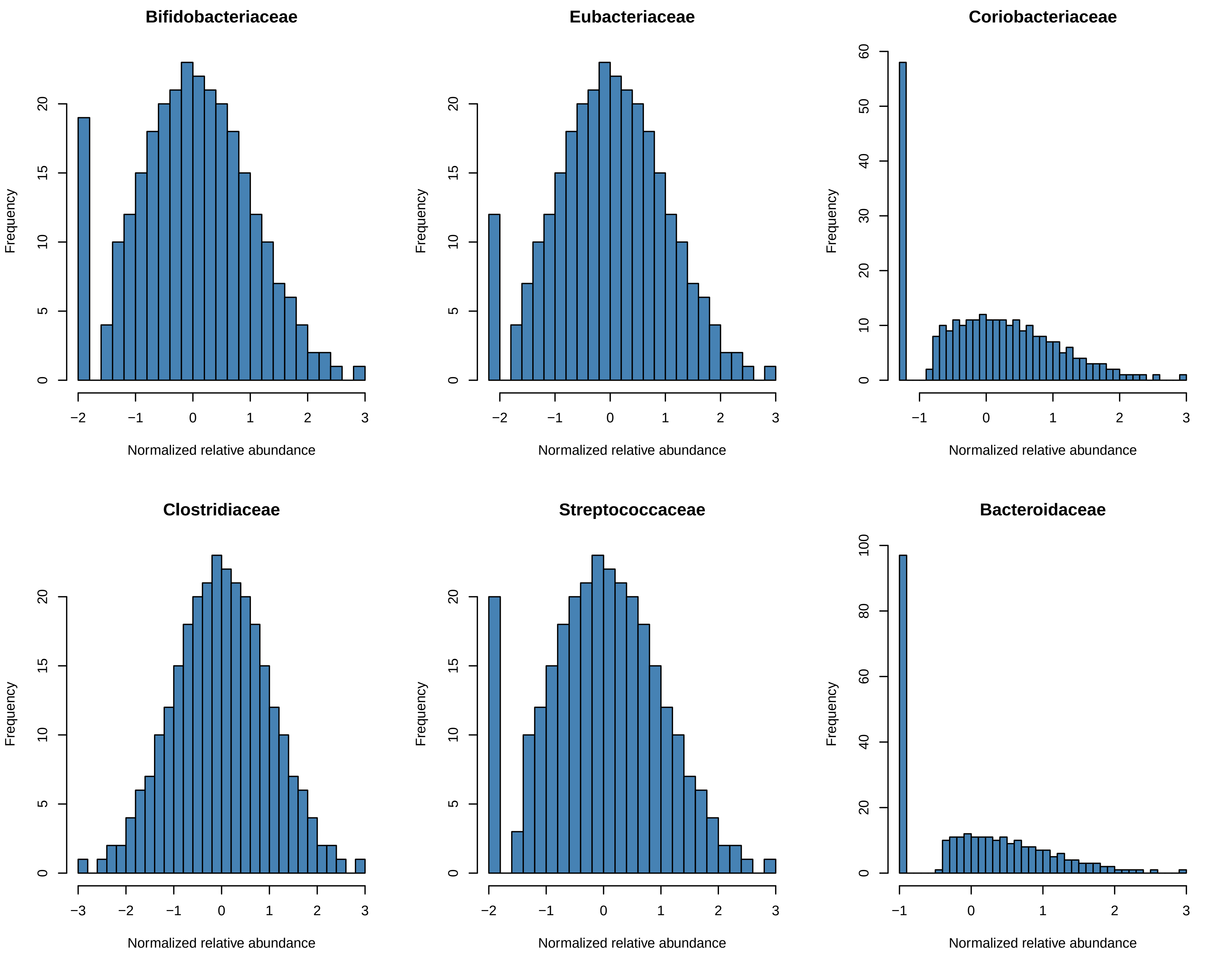

Bifidobacteriaceae
Eubacteriaceae
Coriobacteriaceae
Clostridiaceae
Streptococcaceae
Bacteroidaceae
Frequency
Normalized relative abundance

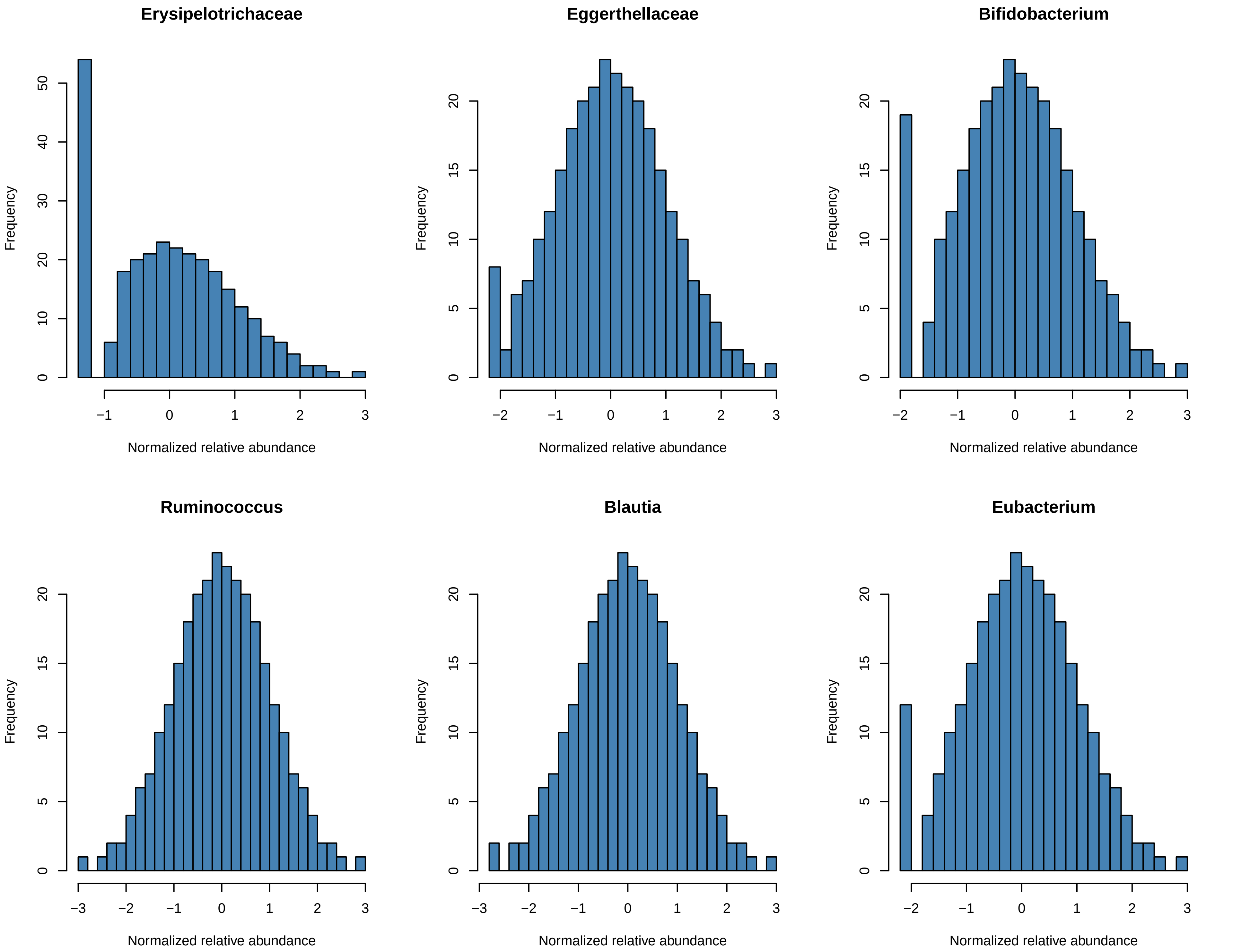
Erysipelotrichaceae
Eggerthellaceae
Bifidobacterium
Ruminococcus
Blautia
Eubacterium
Frequency
Normalized relative abundance
0
10
20
30
40
50
5
15
−3
−2
−1
1
2
3

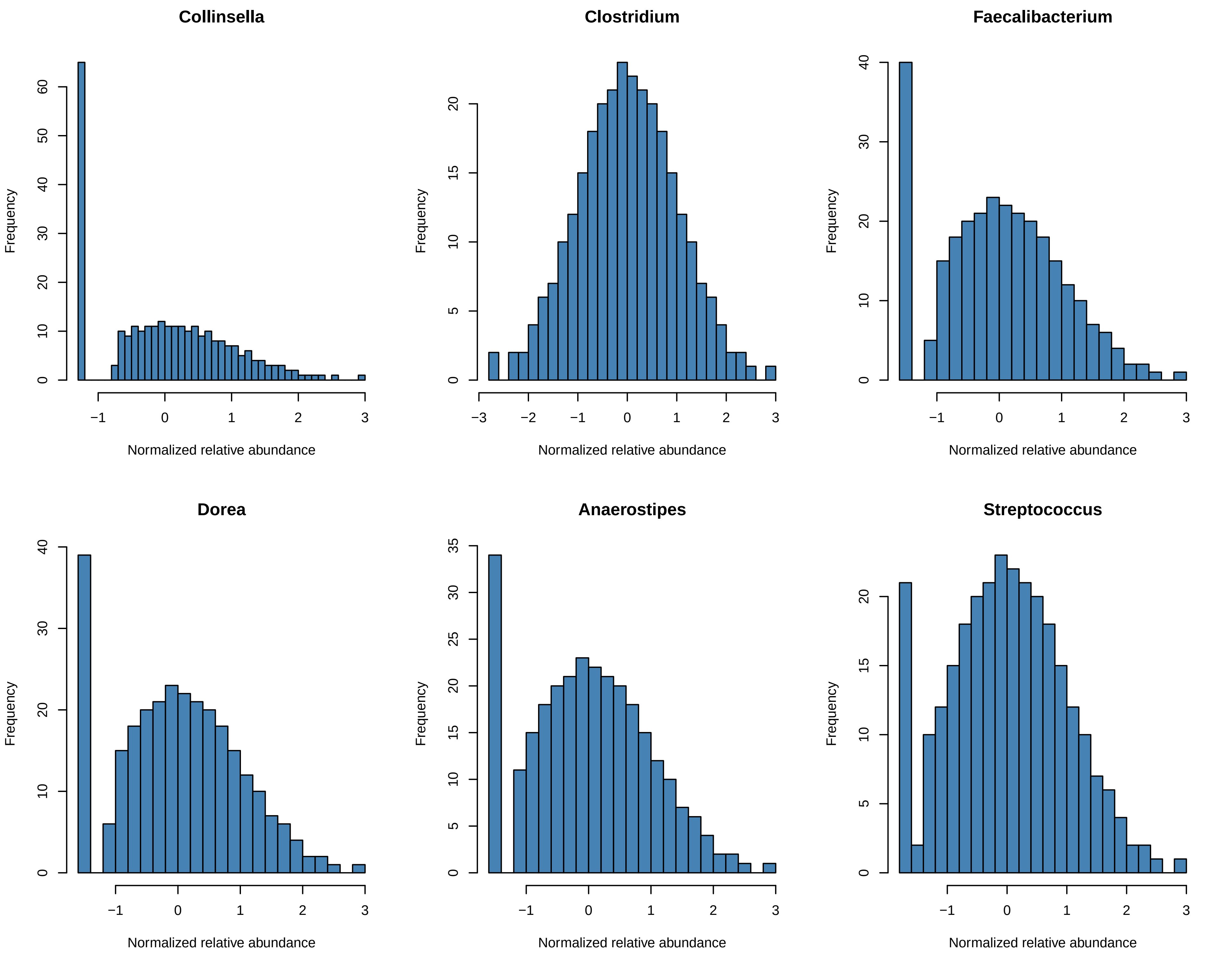

Collinsella
Clostridium
Faecalibacterium
Dorea
Anaerostipes
Streptococcus
Frequency
Normalized relative abundance

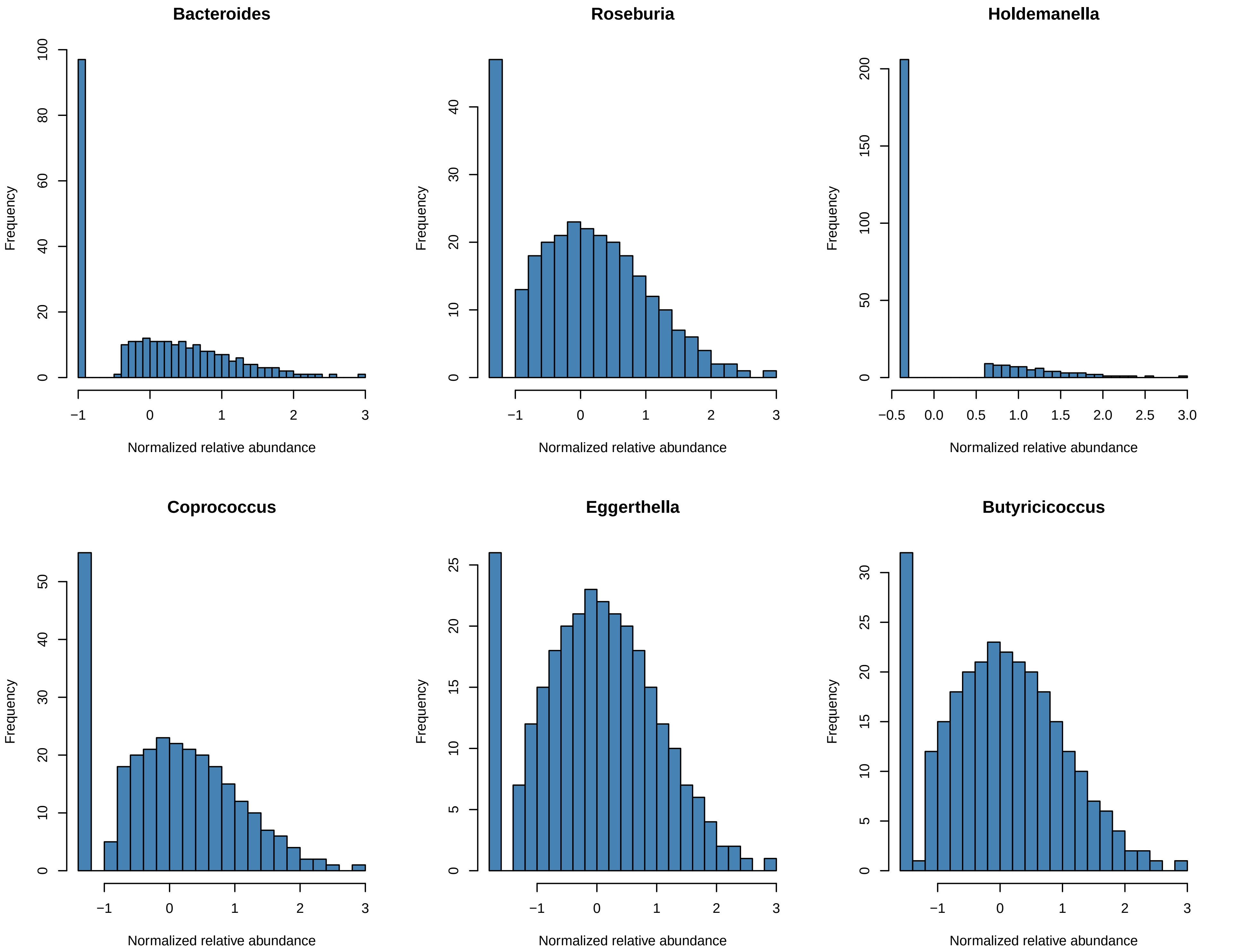
Bacteroides
Frequency
Normalized relative abundance
Roseburia
Frequency
Normalized relative abundance
Holdemanella
Frequency
Normalized relative abundance
Coprococcus
Frequency
Normalized relative abundance
Eggerthella
Frequency
Normalized relative abundance
Butyricicoccus
Frequency
Normalized relative abundance

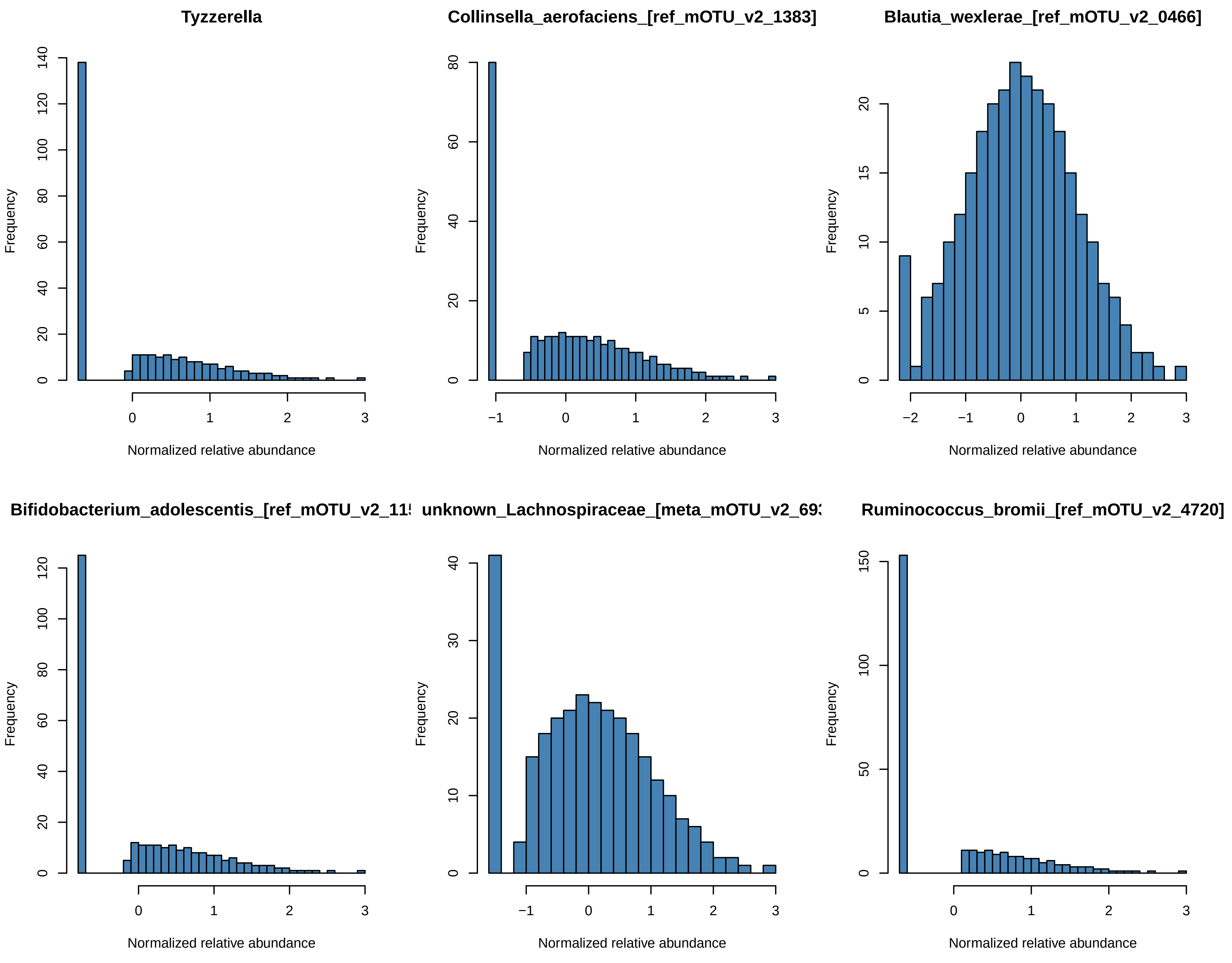

Tyzzerella
Frequency
0
20
40
60
80
100
120
140
0
1
2
3
Normalized relative abundance
Collinsella_aerofaciens_[ref_mOTU_v2_1383]
Frequency
0
20
40
60
80
−1
0
1
2
3
Normalized relative abundance
Blautia_wexlerae_[ref_mOTU_v2_0466]
Frequency
0
5
10
15
20
−2
−1
0
1
2
3
Normalized relative abundance
Bifidobacterium_adolescentis_[ref_mOTU_v2_11
Frequency
0
20
40
60
80
100
120
0
1
2
3
Normalized relative abundance
unknown_Lachnospiraceae_[meta_mOTU_v2_69
Frequency
0
10
20
30
40
−1
0
1
2
3
Normalized relative abundance
Ruminococcus_bromii_[ref_mOTU_v2_4720]
Frequency
0
50
100
150
0
1
2
3
Normalized relative abundance

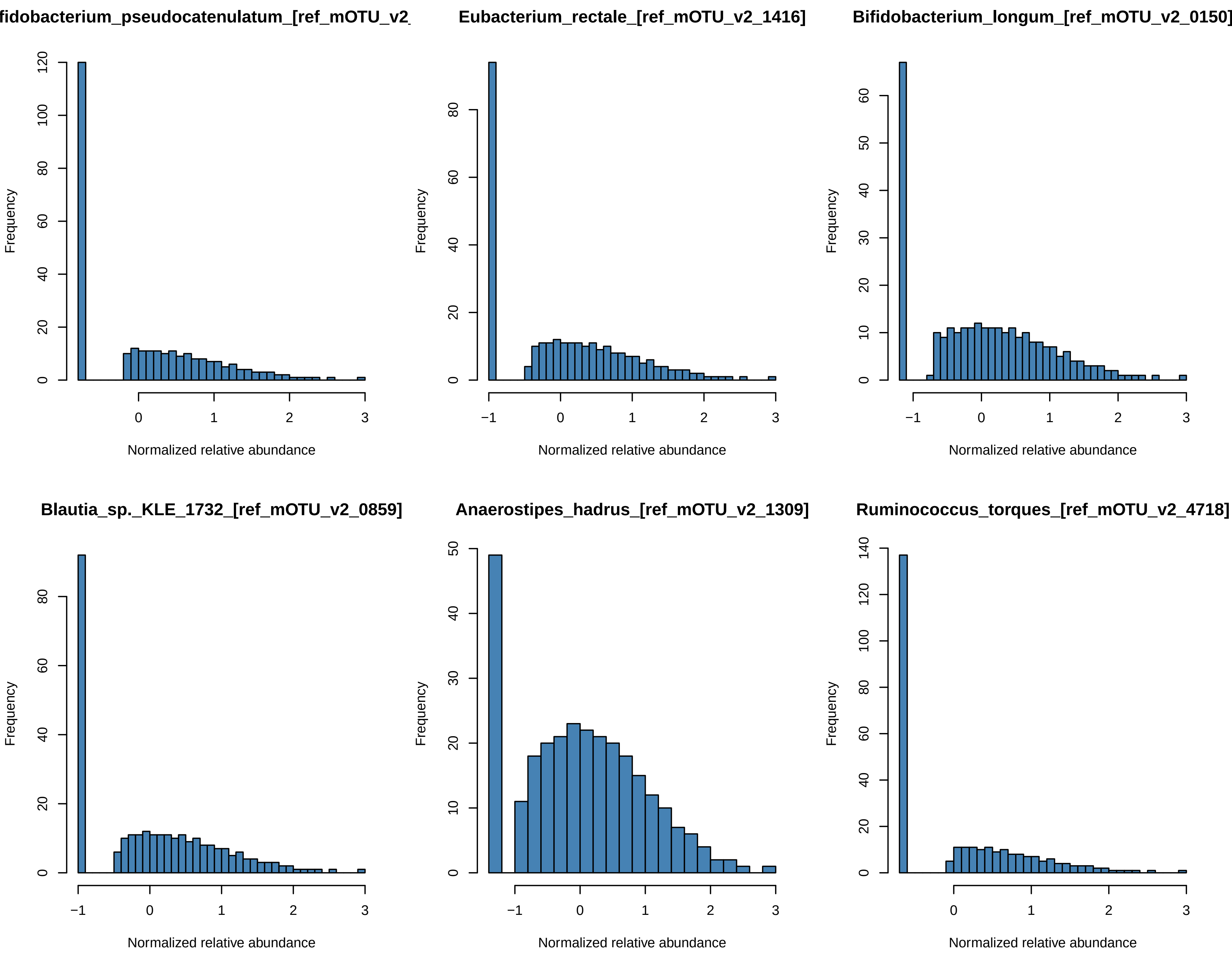

fidobacterium_pseudocatenulatum_[ref_mOTU_v2_
Eubacterium_rectale_[ref_mOTU_v2_1416]
Bifidobacterium_longum_[ref_mOTU_v2_0150]
Blautia_sp._KLE_1732_[ref_mOTU_v2_0859]
Anaerostipes_hadrus_[ref_mOTU_v2_1309]
Ruminococcus_torques_[ref_mOTU_v2_4718]
Frequency
Normalized relative abundance

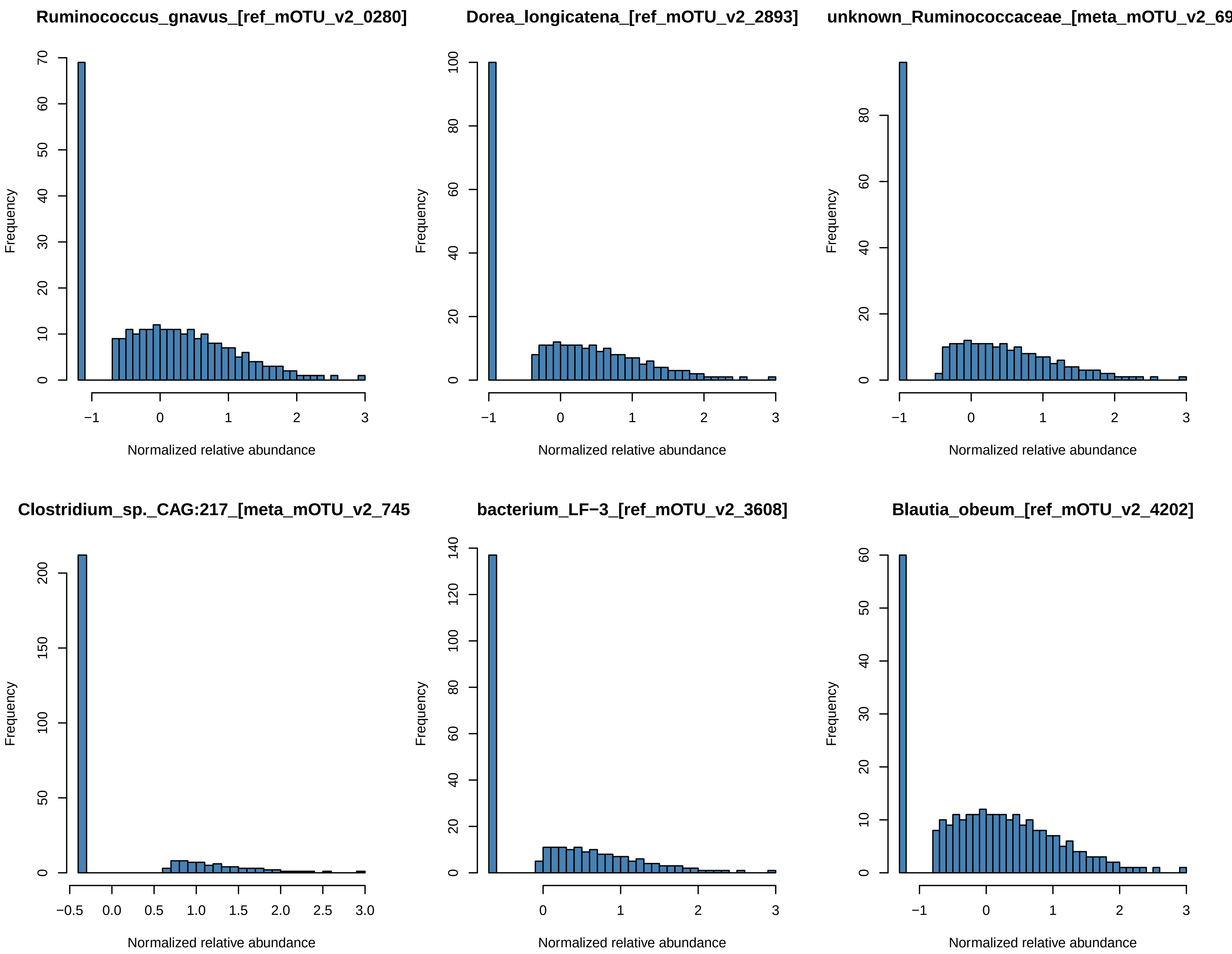
Ruminococcus_gnavus_[ref_mOTU_v2_0280]
Dorea_longicatena_[ref_mOTU_v2_2893]
unknown_Ruminococcaceae_[meta_mOTU_v2_69
Clostridium_sp._CAG:217_[meta_mOTU_v2_745
bacterium_LF−3_[ref_mOTU_v2_3608]
Blautia_obeum_[ref_mOTU_v2_4202]
Frequency
Normalized relative abundance

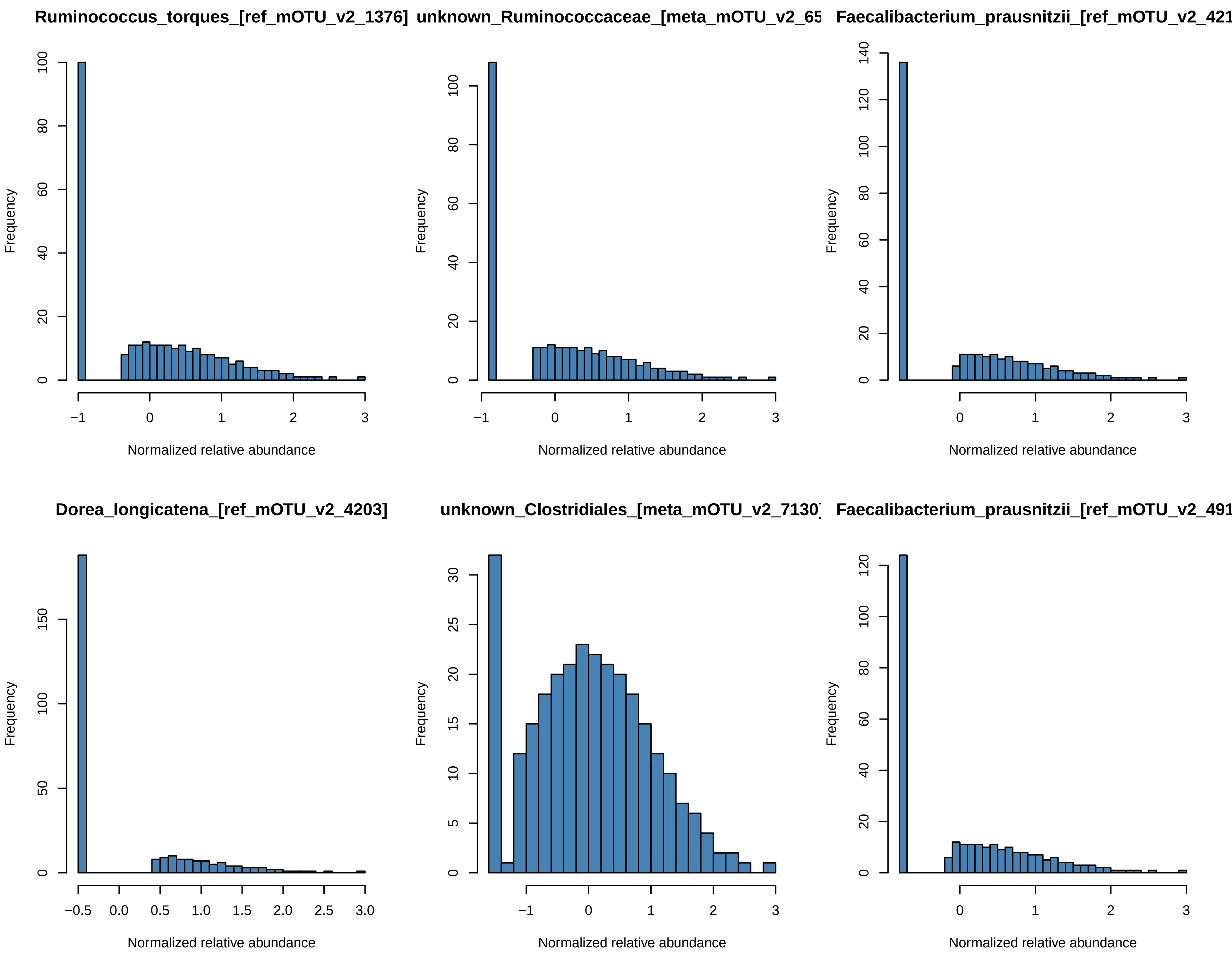
Ruminococcus_torques_[ref_mOTU_v2_1376]
unknown_Ruminococcaceae_[meta_mOTU_v2_65
Faecalibacterium_prausnitzii_[ref_mOTU_v2_421
Dorea_longicatena_[ref_mOTU_v2_4203]
unknown_Clostridiales_[meta_mOTU_v2_7130]
Faecalibacterium_prausnitzii_[ref_mOTU_v2_491
Frequency
Normalized relative abundance

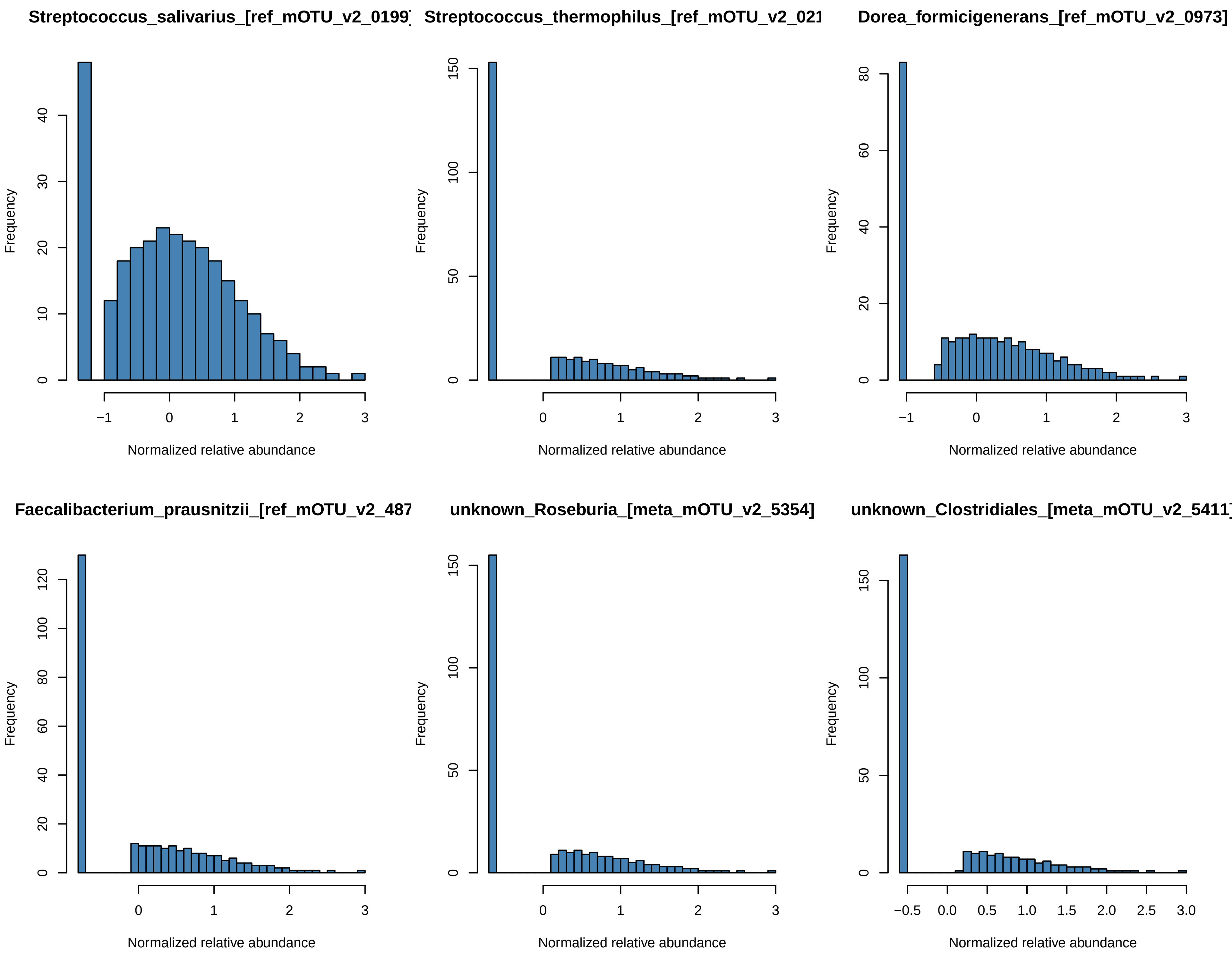

Streptococcus_salivarius_[ref_mOTU_v2_0199]
Streptococcus_thermophilus_[ref_mOTU_v2_021
Dorea_formicigenerans_[ref_mOTU_v2_0973]
Faecalibacterium_prausnitzii_[ref_mOTU_v2_487
unknown_Roseburia_[meta_mOTU_v2_5354]
unknown_Clostridiales_[meta_mOTU_v2_5411]
Frequency
Normalized relative abundance

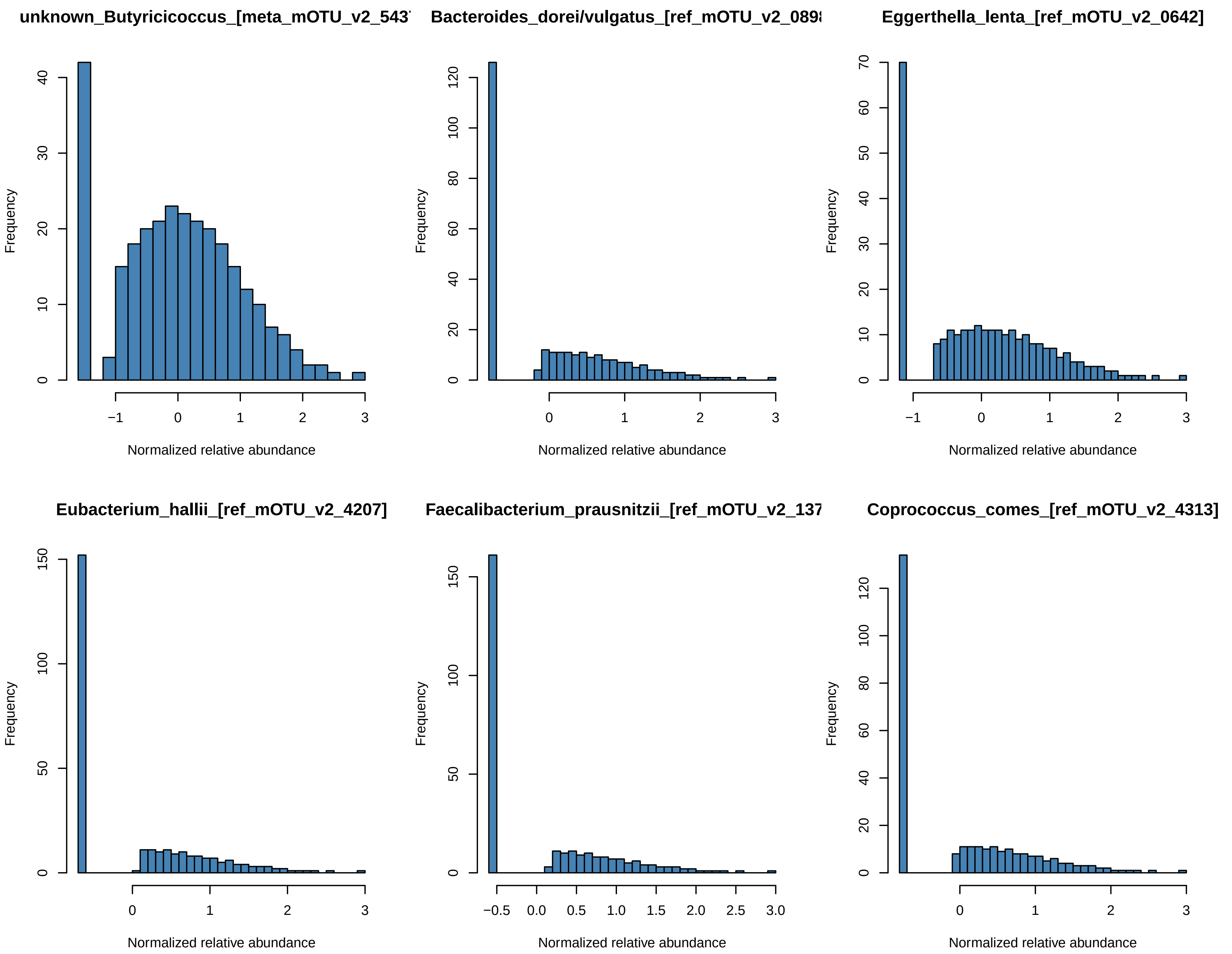

unknown_Butyricicoccus_[meta_mOTU_v2_543
Bacteroides_dorei/vulgatus_[ref_mOTU_v2_089
Eggerthella_lenta_[ref_mOTU_v2_0642]
Eubacterium_hallii_[ref_mOTU_v2_4207]
Faecalibacterium_prausnitzii_[ref_mOTU_v2_137
Coprococcus_comes_[ref_mOTU_v2_4313]
Frequency
Normalized relative abundance

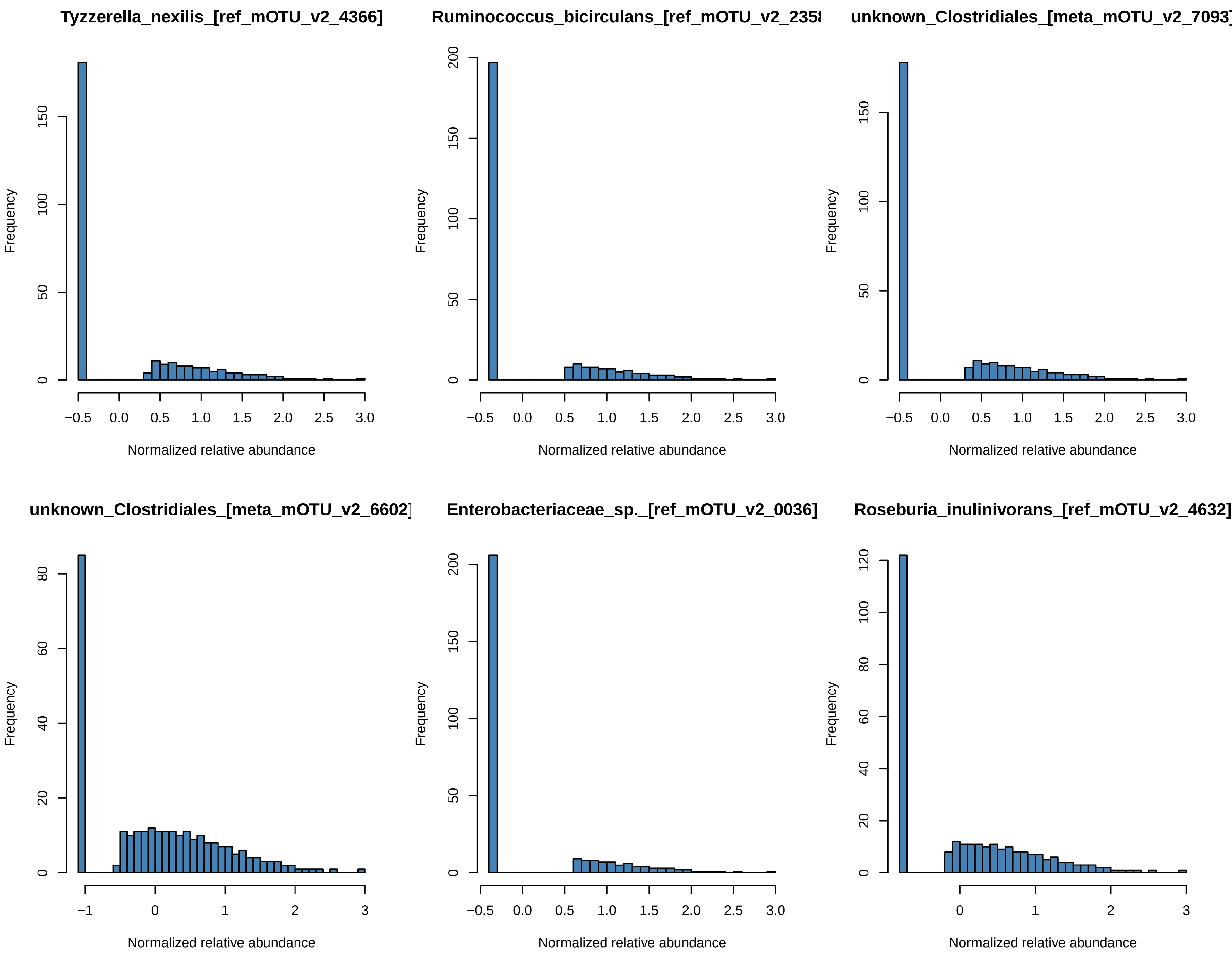
Tyzzerella_nexilis_[ref_mOTU_v2_4366]
Frequency
Normalized relative abundance
Ruminococcus_bicirculans_[ref_mOTU_v2_235
Frequency
Normalized relative abundance
unknown_Clostridiales_[meta_mOTU_v2_7093
Frequency
Normalized relative abundance
unknown_Clostridiales_[meta_mOTU_v2_6602]
Frequency
Normalized relative abundance
Enterobacteriaceae_sp._[ref_mOTU_v2_0036]
Frequency
Normalized relative abundance
Roseburia_inulinivorans_[ref_mOTU_v2_4632]
Frequency
Normalized relative abundance

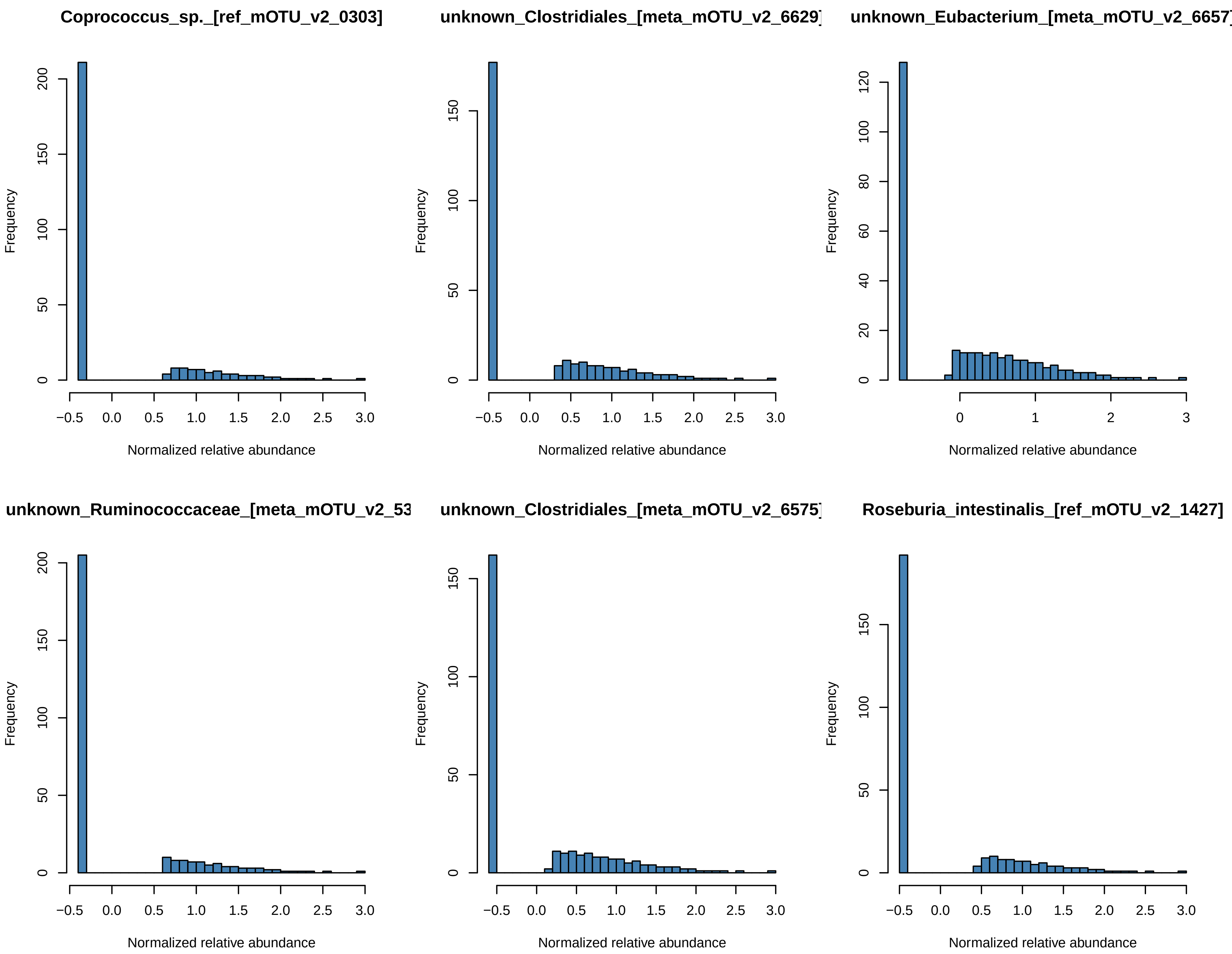

Coprococcus_sp._[ref_mOTU_v2_0303]
unknown_Clostridiales_[meta_mOTU_v2_6629]
unknown_Eubacterium_[meta_mOTU_v2_6657
unknown_Ruminococcaceae_[meta_mOTU_v2_53
unknown_Clostridiales_[meta_mOTU_v2_6575]
Roseburia_intestinalis_[ref_mOTU_v2_1427]
Frequency
Normalized relative abundance

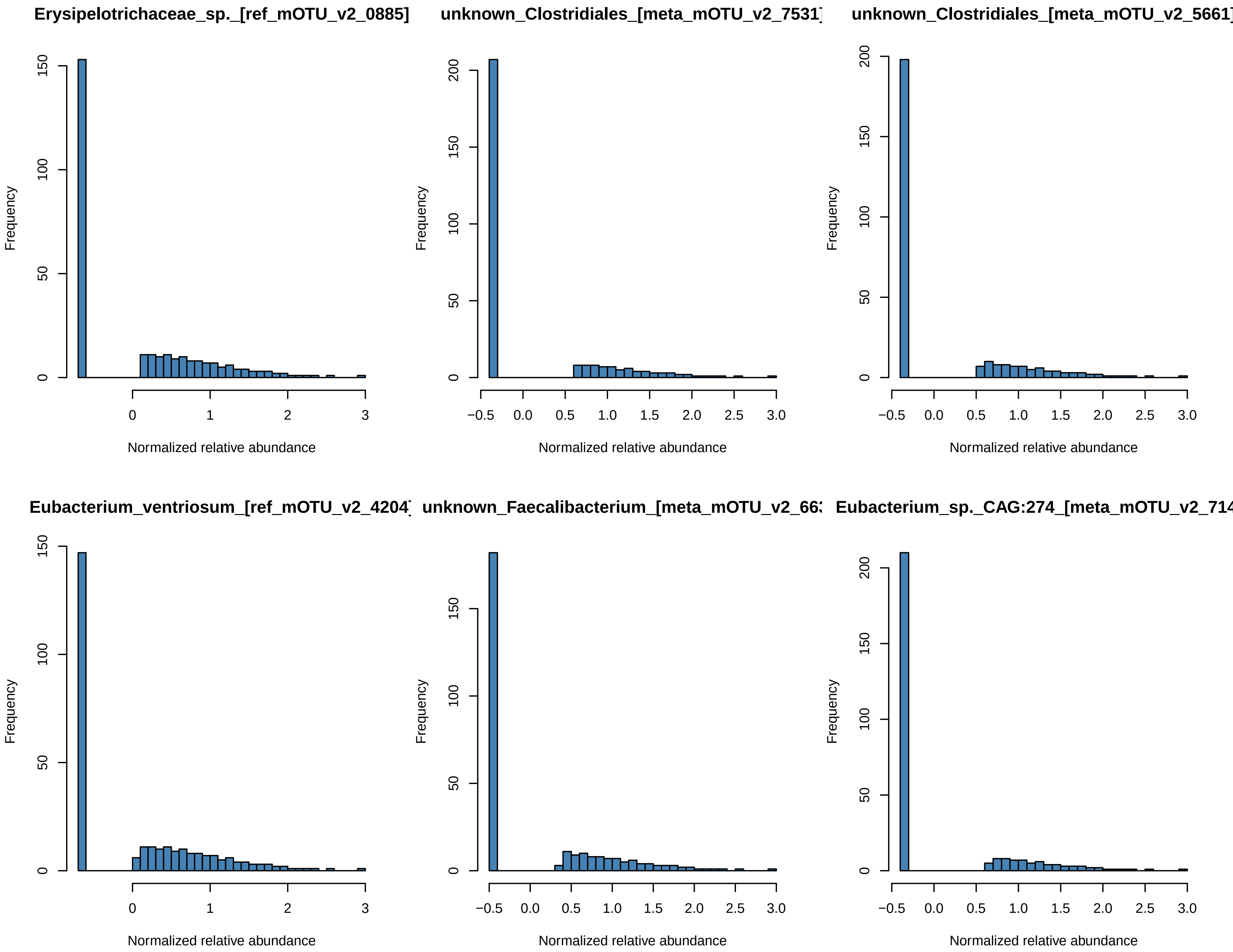

Erysipelotrichaceae_sp._[ref_mOTU_v2_0885]
unknown_Clostridiales_[meta_mOTU_v2_7531]
unknown_Clostridiales_[meta_mOTU_v2_5661]
Eubacterium_ventriosum_[ref_mOTU_v2_4204]
unknown_Faecalibacterium_[meta_mOTU_v2_66
Eubacterium_sp._CAG:274_[meta_mOTU_v2_714
Frequency
Normalized relative abundance

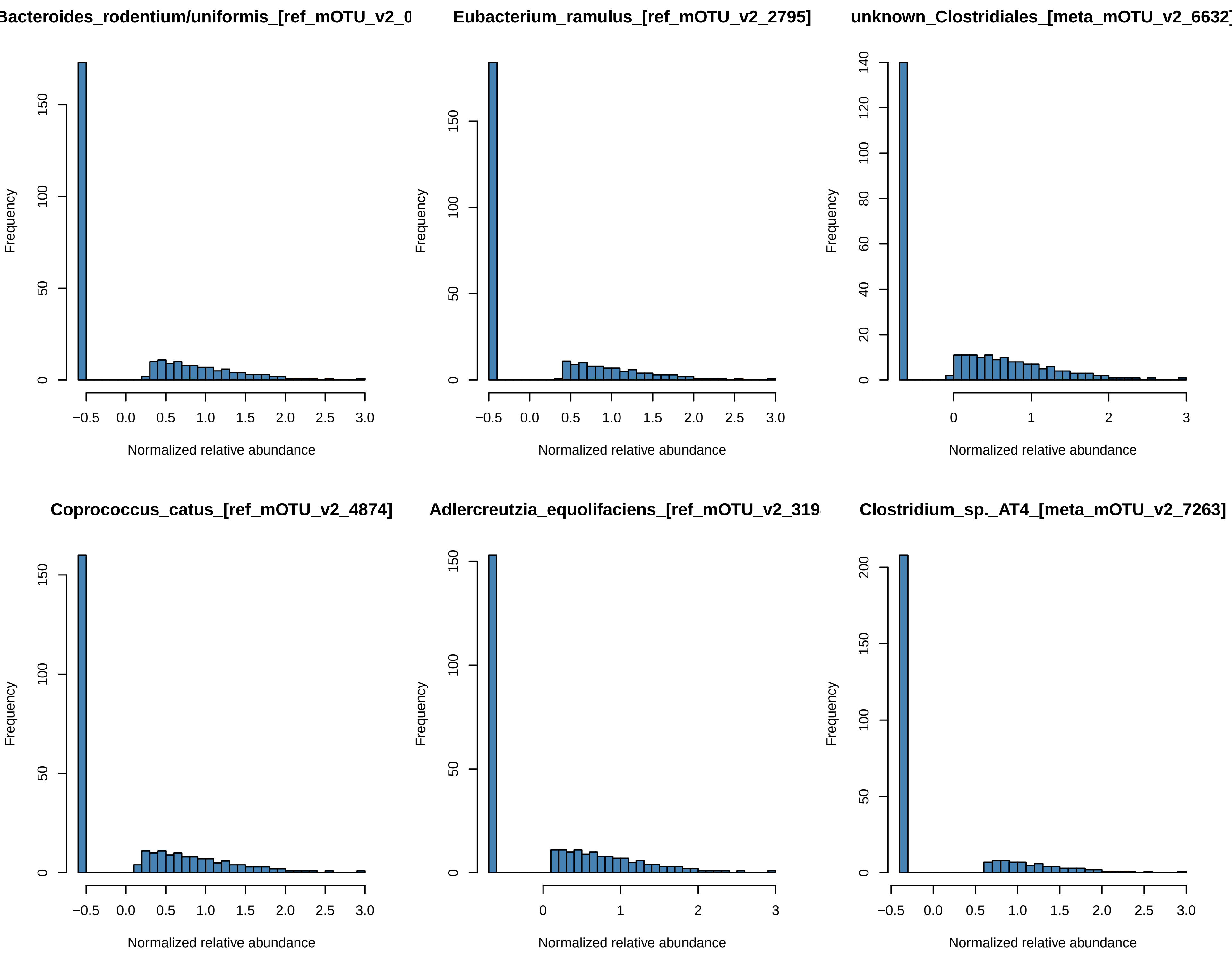

Bacteroides_rodentium/uniformis_[ref_mOTU_v2_0
Eubacterium_ramulus_[ref_mOTU_v2_2795]
unknown_Clostridiales_[meta_mOTU_v2_6632]
Coprococcus_catus_[ref_mOTU_v2_4874]
Adlercreutzia_equolifaciens_[ref_mOTU_v2_319
Clostridium_sp._AT4_[meta_mOTU_v2_7263]
Frequency
Normalized relative abundance
0
50
100
150
20
40
60
80
120
140
200
−0.5
0.0
0.5
1.0
1.5
2.0
2.5
3.0
1
2
3

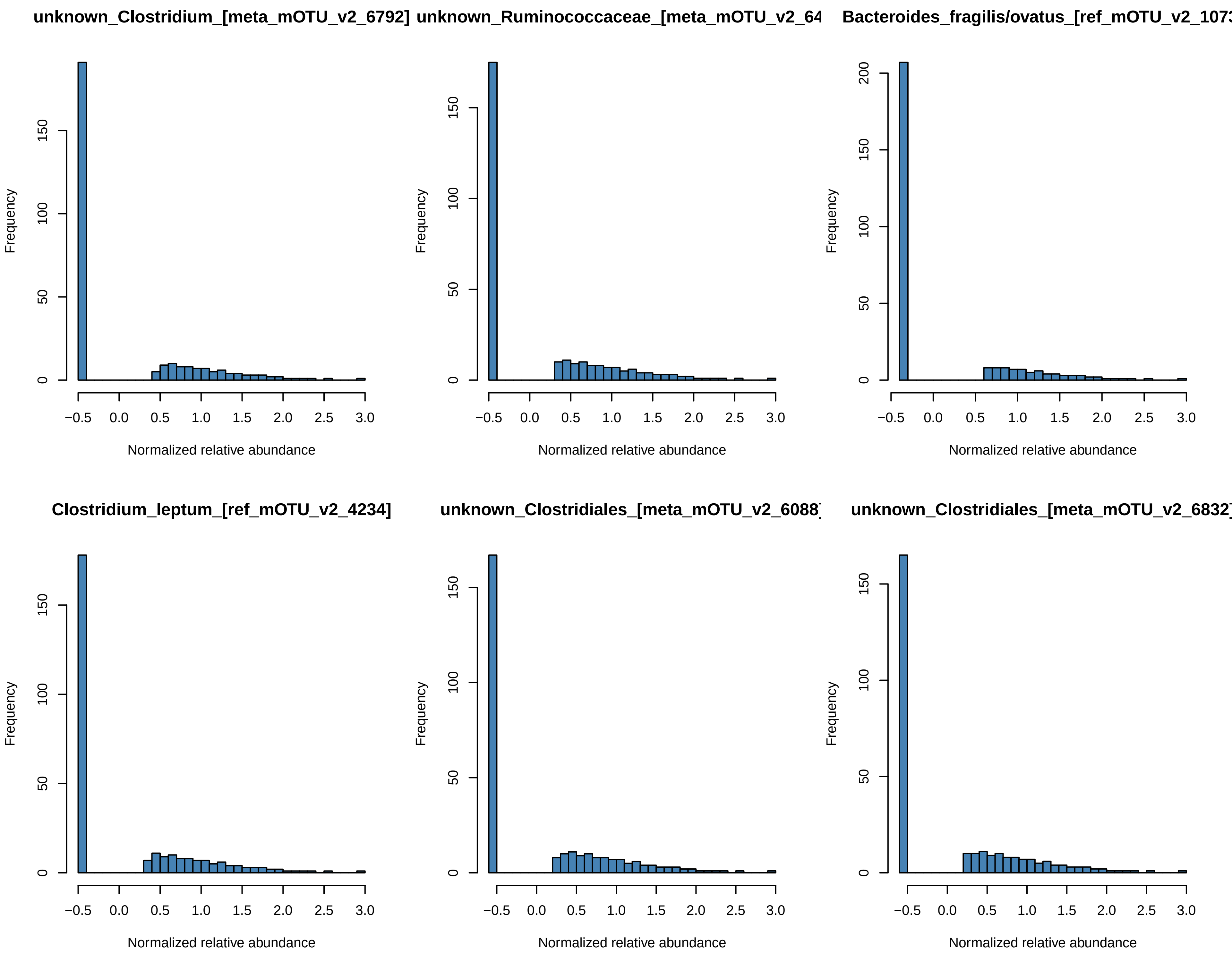
unknown_Clostridium_[meta_mOTU_v2_6792]
unknown_Ruminococcaceae_[meta_mOTU_v2_64
Bacteroides_fragilis/ovatus_[ref_mOTU_v2_107
Clostridium_leptum_[ref_mOTU_v2_4234]
unknown_Clostridiales_[meta_mOTU_v2_6088]
unknown_Clostridiales_[meta_mOTU_v2_6832]
Frequency
Normalized relative abundance
−0.5 0.0 0.5 1.0 1.5 2.0 2.5 3.0
0 50 100 150 200

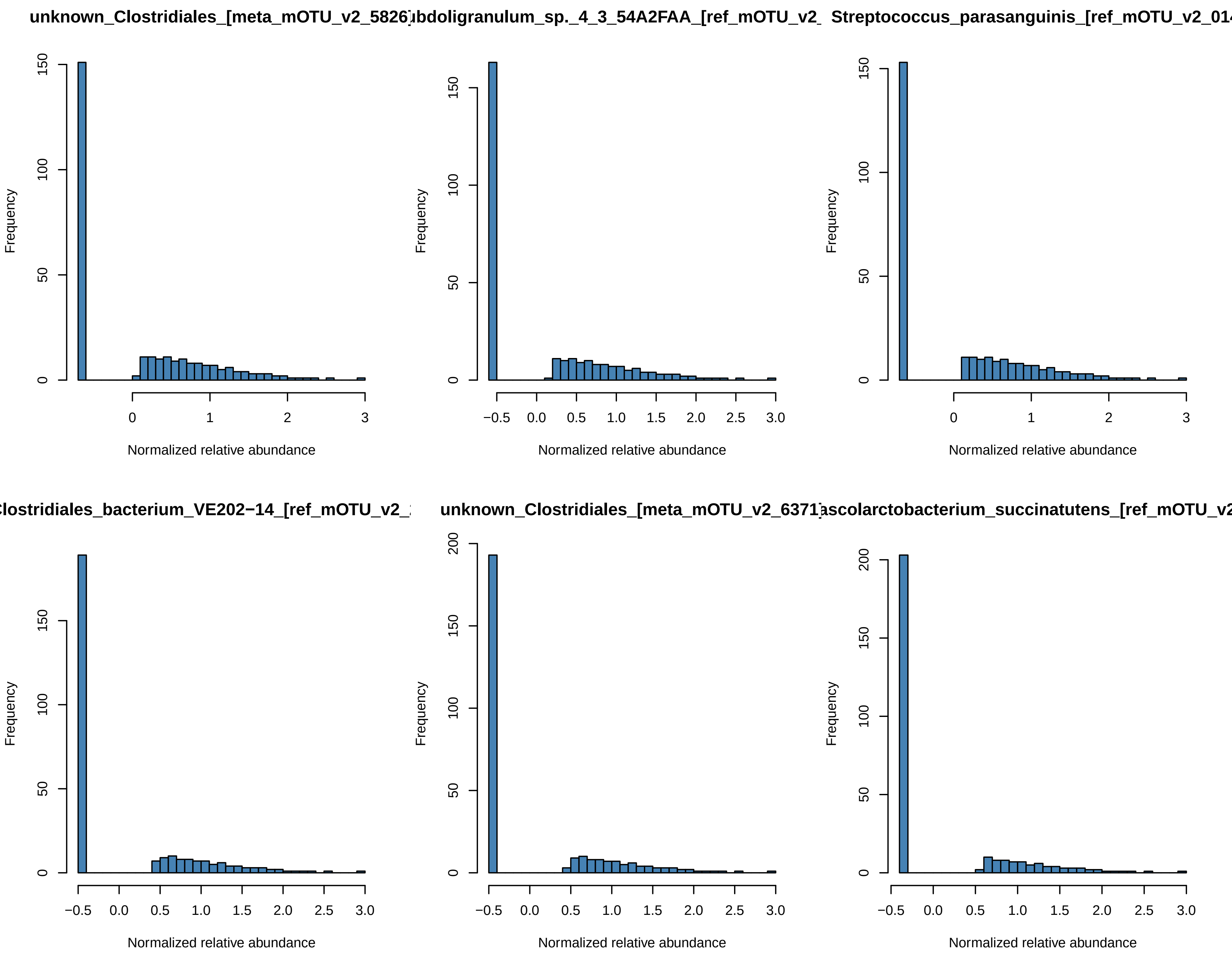
unknown_Clostridiales_[meta_mOTU_v2_5826]
ubdoligranulum_sp._4_3_54A2FAA_[ref_mOTU_v2_
Streptococcus_parasanguinis_[ref_mOTU_v2_01
Clostridiales_bacterium_VE202−14_[ref_mOTU_v2_
unknown_Clostridiales_[meta_mOTU_v2_6371]
ascolarctobacterium_succinatutens_[ref_mOTU_v2
Frequency
Normalized relative abundance

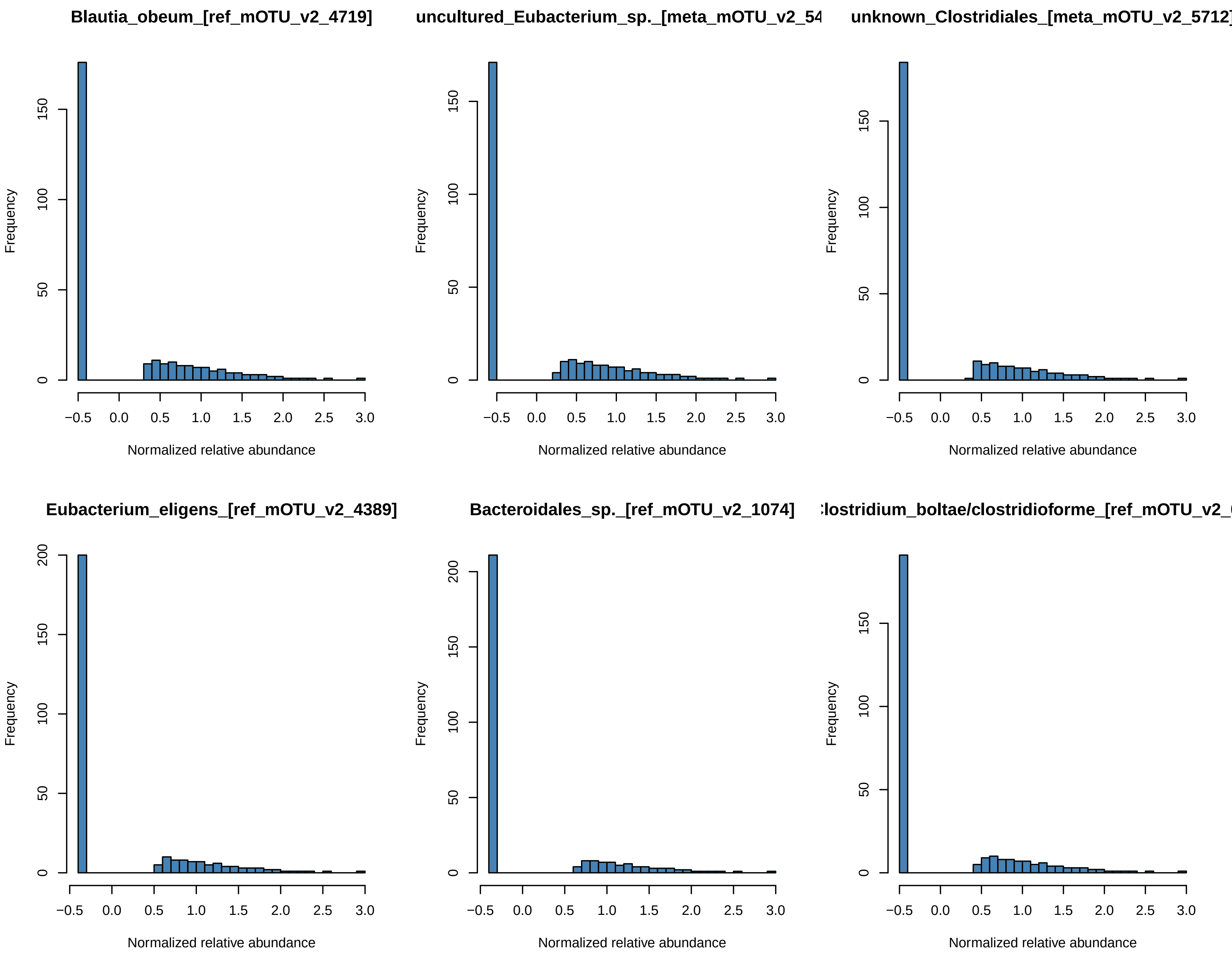
Blautia_obeum_[ref_mOTU_v2_4719]
uncultured_Eubacterium_sp._[meta_mOTU_v2_54
unknown_Clostridiales_[meta_mOTU_v2_5712]
Eubacterium_eligens_[ref_mOTU_v2_4389]
Bacteroidales_sp._[ref_mOTU_v2_1074]
lostridium_boltae/clostridioforme_[ref_mOTU_v2_
Frequency
Normalized relative abundance
0
50
100
150
200
−0.5
0.0
0.5
1.0
1.5
2.0
2.5
3.0

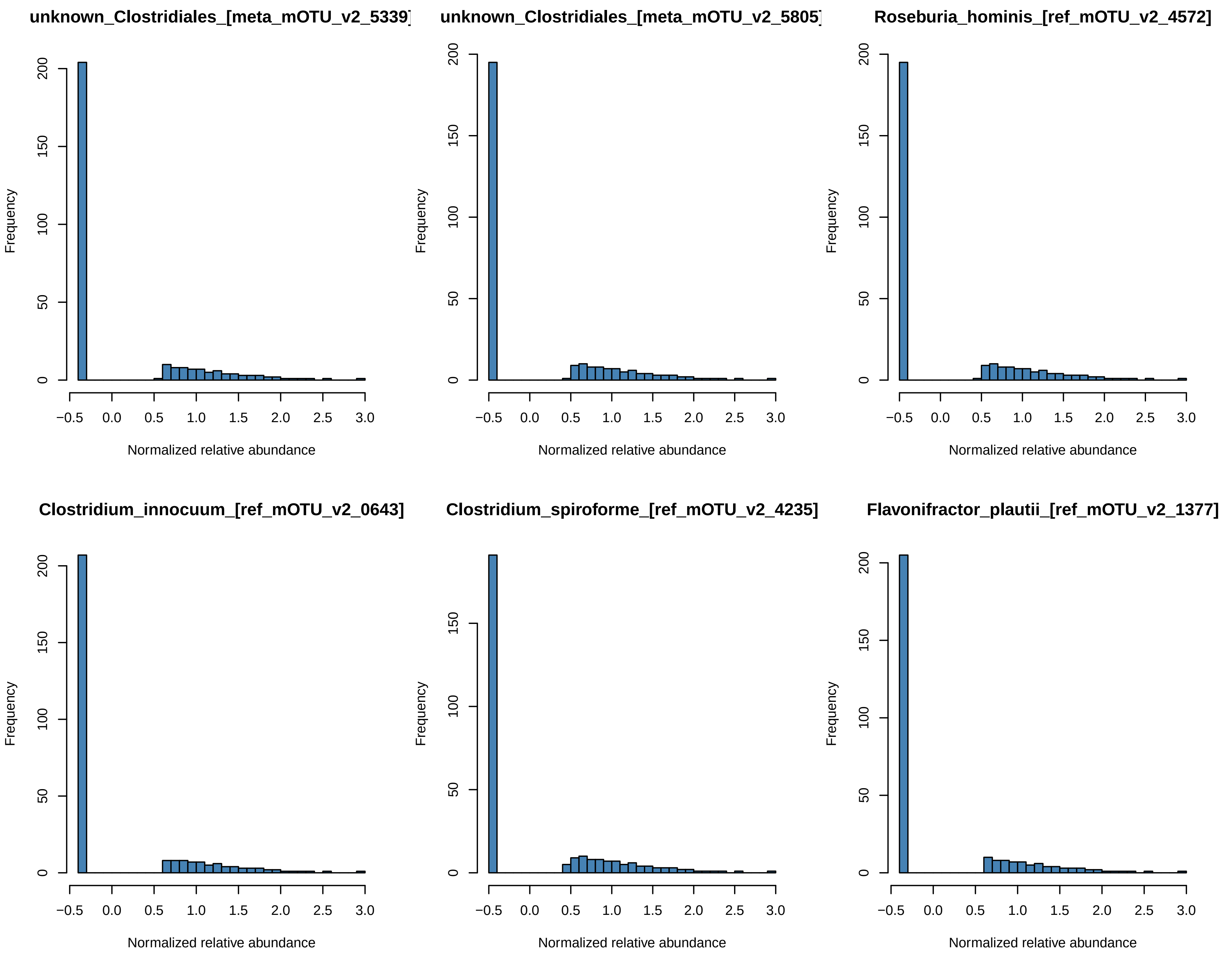
unknown_Clostridiales_[meta_mOTU_v2_5339]
unknown_Clostridiales_[meta_mOTU_v2_5805]
Roseburia_hominis_[ref_mOTU_v2_4572]
Clostridium_innocuum_[ref_mOTU_v2_0643]
Clostridium_spiroforme_[ref_mOTU_v2_4235]
Flavonifractor_plautii_[ref_mOTU_v2_1377]
Frequency
Normalized relative abundance
0
50
100
150
200
−0.5
0.0
0.5
1.0
1.5
2.0
2.5
3.0

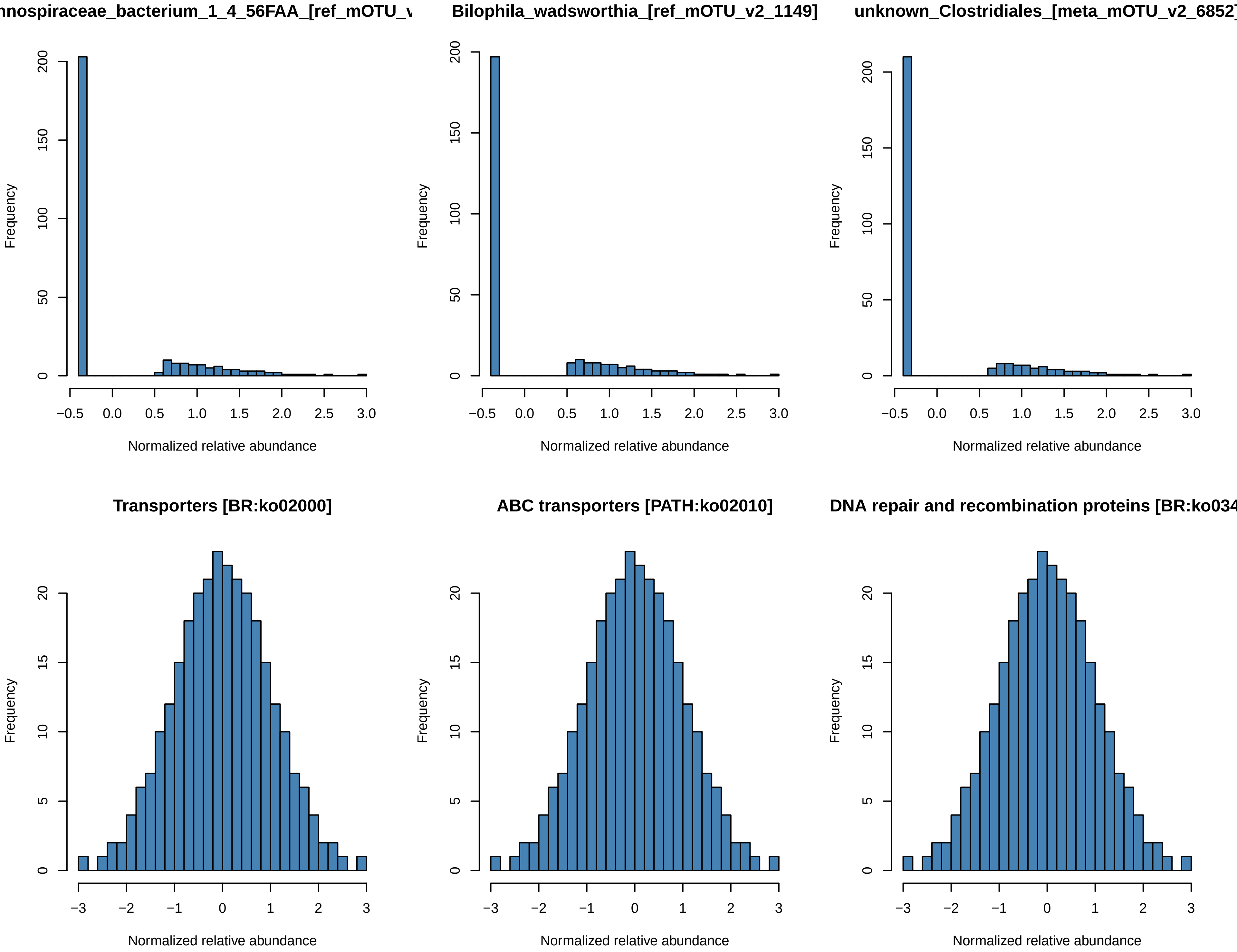

nnospiraceae_bacterium_1_4_56FAA_[ref_mOTU_v
Bilophila_wadsworthia_[ref_mOTU_v2_1149]
unknown_Clostridiales_[meta_mOTU_v2_6852]
Frequency
Normalized relative abundance
Transporters [BR:ko02000]
ABC transporters [PATH:ko02010]
DNA repair and recombination proteins [BR:ko034

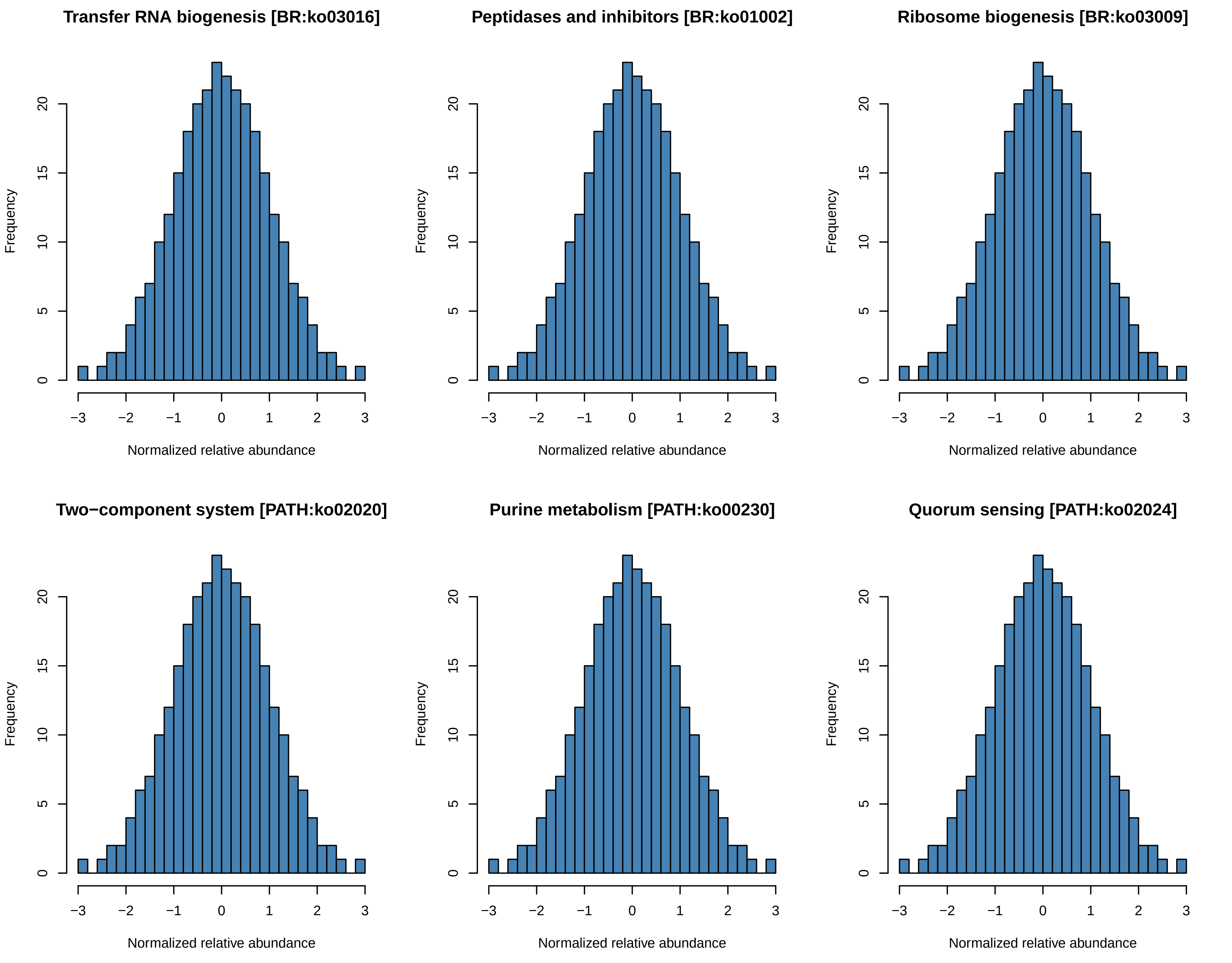
Transfer RNA biogenesis [BR:ko03016]
Peptidases and inhibitors [BR:ko01002]
Ribosome biogenesis [BR:ko03009]
Two−component system [PATH:ko02020]
Purine metabolism [PATH:ko00230]
Quorum sensing [PATH:ko02024]
Frequency
0
5
10
15
20
−3
−2
−1
0
1
2
3
Normalized relative abundance

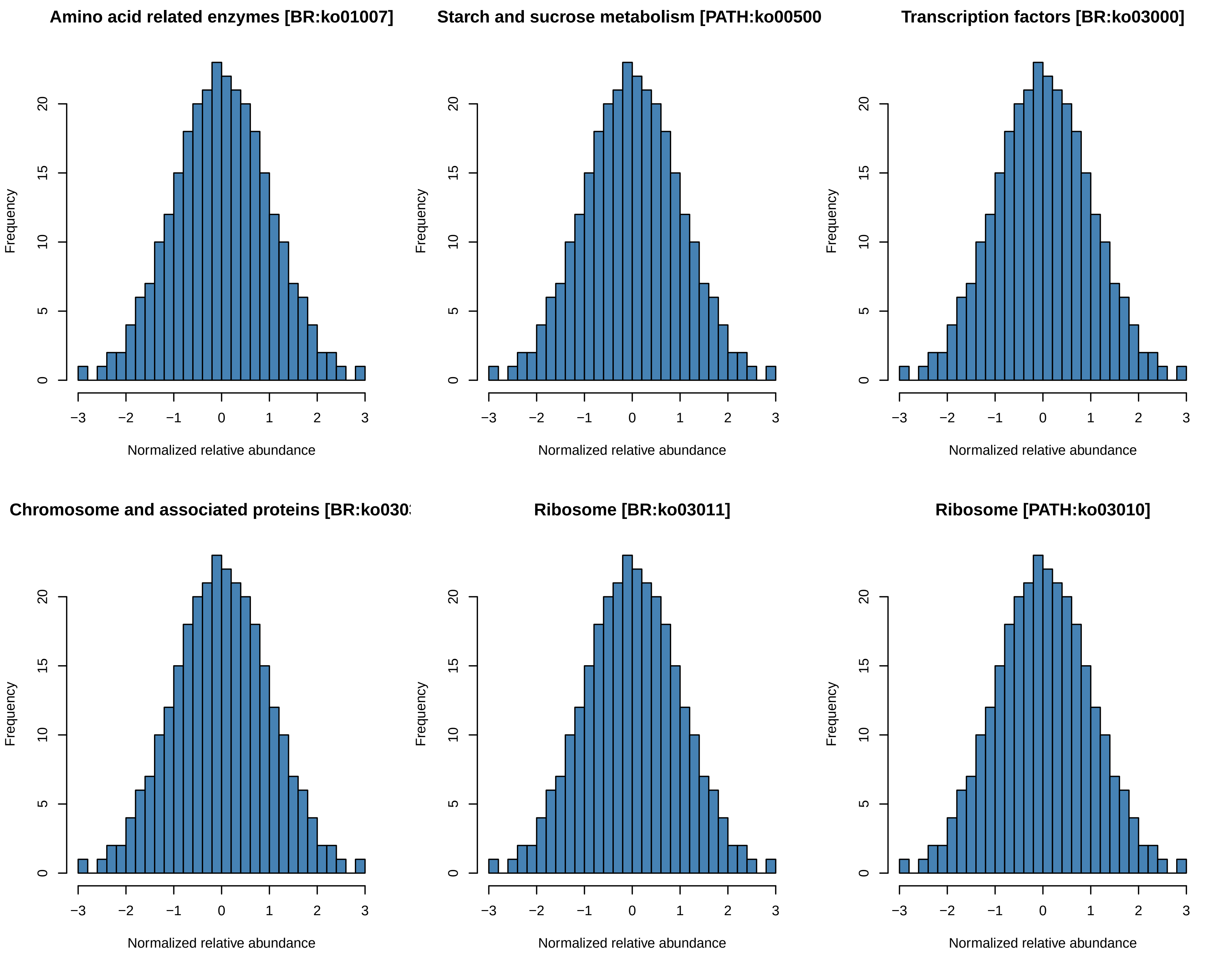
Amino acid related enzymes [BR:ko01007]
Starch and sucrose metabolism [PATH:ko00500
Transcription factors [BR:ko03000]
Chromosome and associated proteins [BR:ko030
Ribosome [BR:ko03011]
Ribosome [PATH:ko03010]
Frequency
0
5
10
15
20
−3
−2
−1
0
1
2
3
Normalized relative abundance

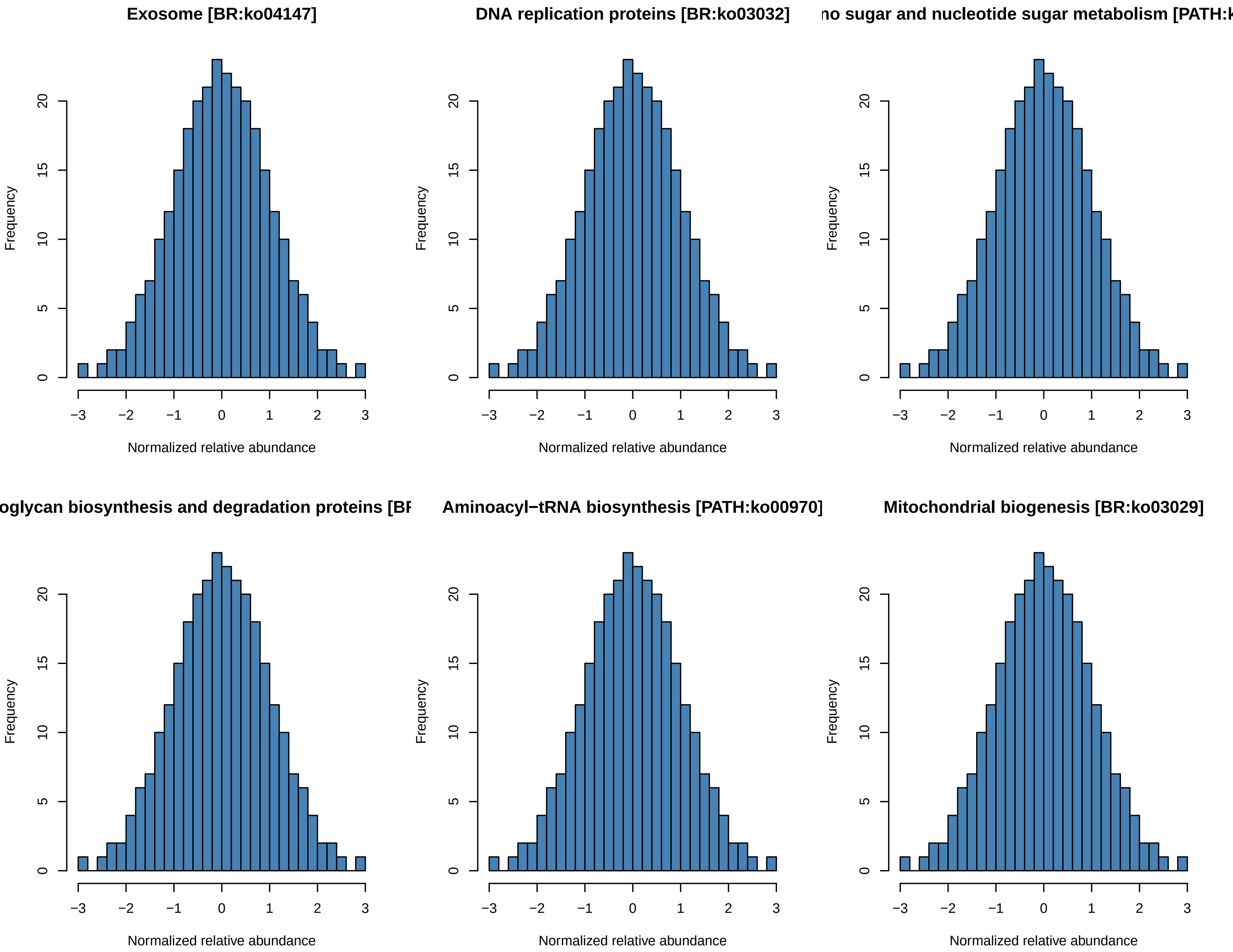

Exosome [BR:ko04147]
DNA replication proteins [BR:ko03032]
no sugar and nucleotide sugar metabolism [PATH:k
oglycan biosynthesis and degradation proteins [BF
Aminoacyl−tRNA biosynthesis [PATH:ko00970]
Mitochondrial biogenesis [BR:ko03029]
Frequency
Normalized relative abundance

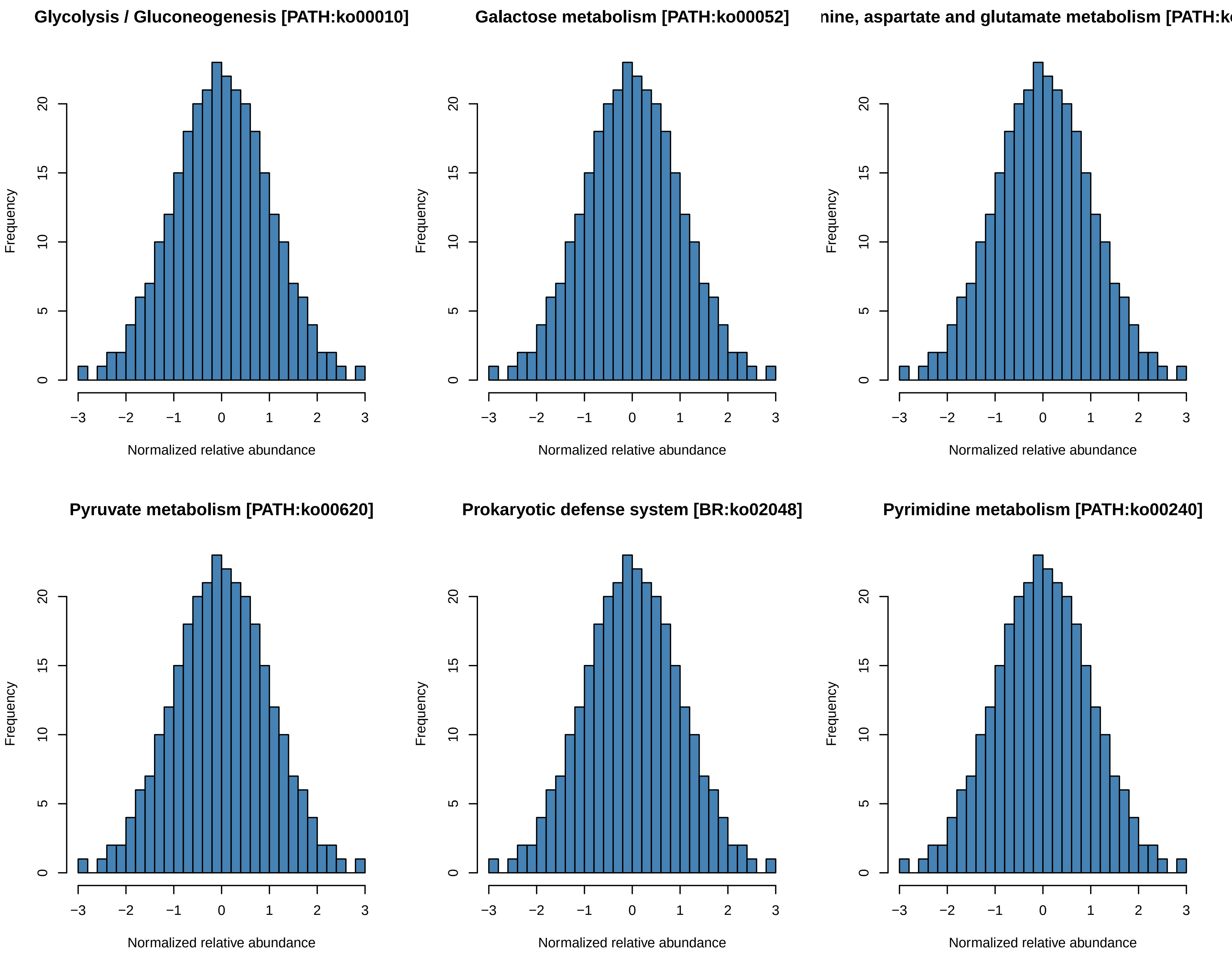
Glycolysis / Gluconeogenesis [PATH:ko00010]
Galactose metabolism [PATH:ko00052]
nine, aspartate and glutamate metabolism [PATH:k
Pyruvate metabolism [PATH:ko00620]
Prokaryotic defense system [BR:ko02048]
Pyrimidine metabolism [PATH:ko00240]
Frequency
Normalized relative abundance

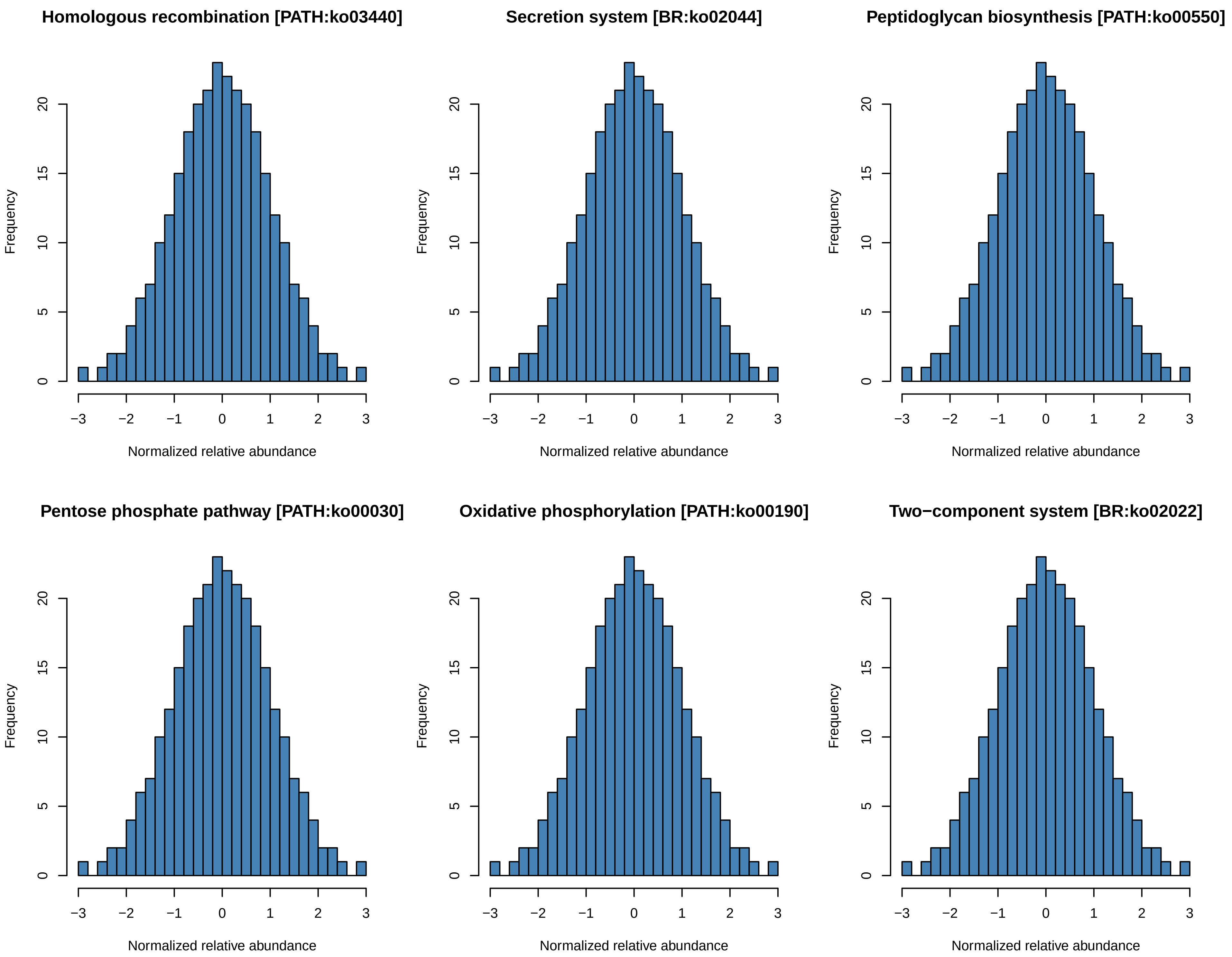

Homologous recombination [PATH:ko03440]
Secretion system [BR:ko02044]
Peptidoglycan biosynthesis [PATH:ko00550]
Pentose phosphate pathway [PATH:ko00030]
Oxidative phosphorylation [PATH:ko00190]
Two−component system [BR:ko02022]
Frequency
Normalized relative abundance
0
5
10
15
20
−3
−2
−1
0
1
2
3

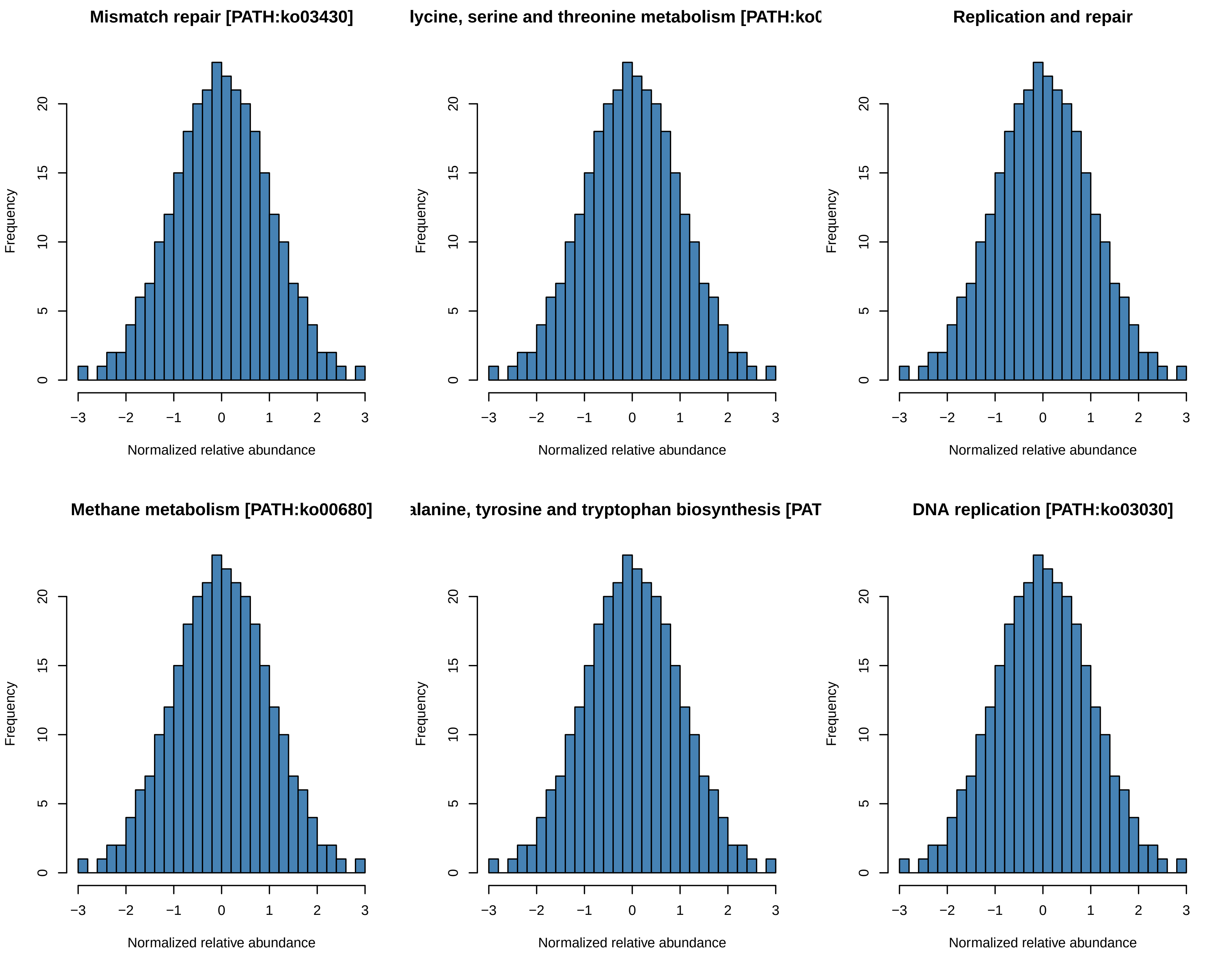

Mismatch repair [PATH:ko03430]
lycine, serine and threonine metabolism [PATH:ko0
Replication and repair
Methane metabolism [PATH:ko00680]
alanine, tyrosine and tryptophan biosynthesis [PAT
DNA replication [PATH:ko03030]
Frequency
0
5
10
15
20
−3
−2
−1
0
1
2
3
Normalized relative abundance

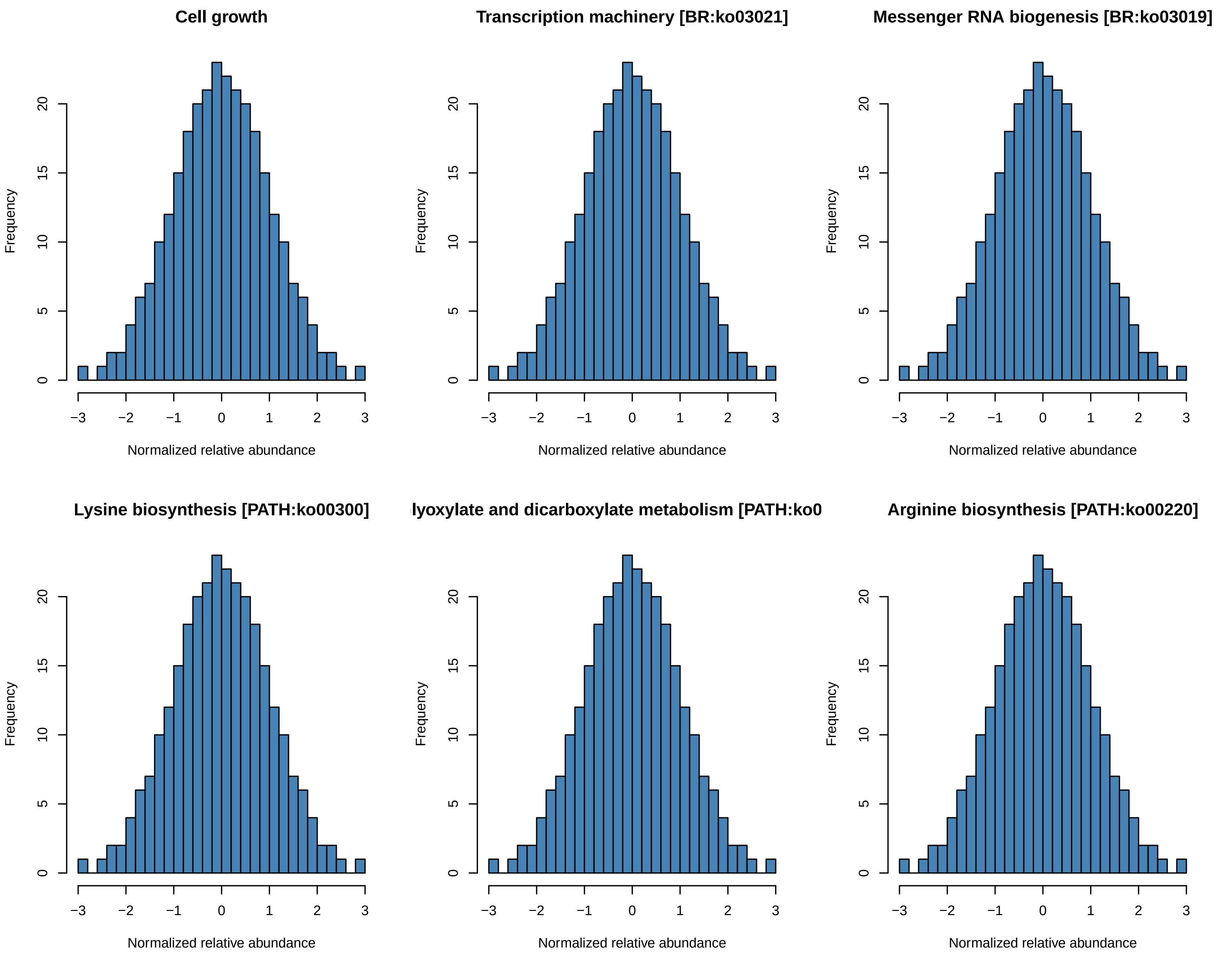
Cell growth
Transcription machinery [BR:ko03021]
Messenger RNA biogenesis [BR:ko03019]
Lysine biosynthesis [PATH:ko00300]
lyoxylate and dicarboxylate metabolism [PATH:ko0
Arginine biosynthesis [PATH:ko00220]
Frequency
Normalized relative abundance
0
5
10
15
20
−3
−2
−1
0
1
2
3

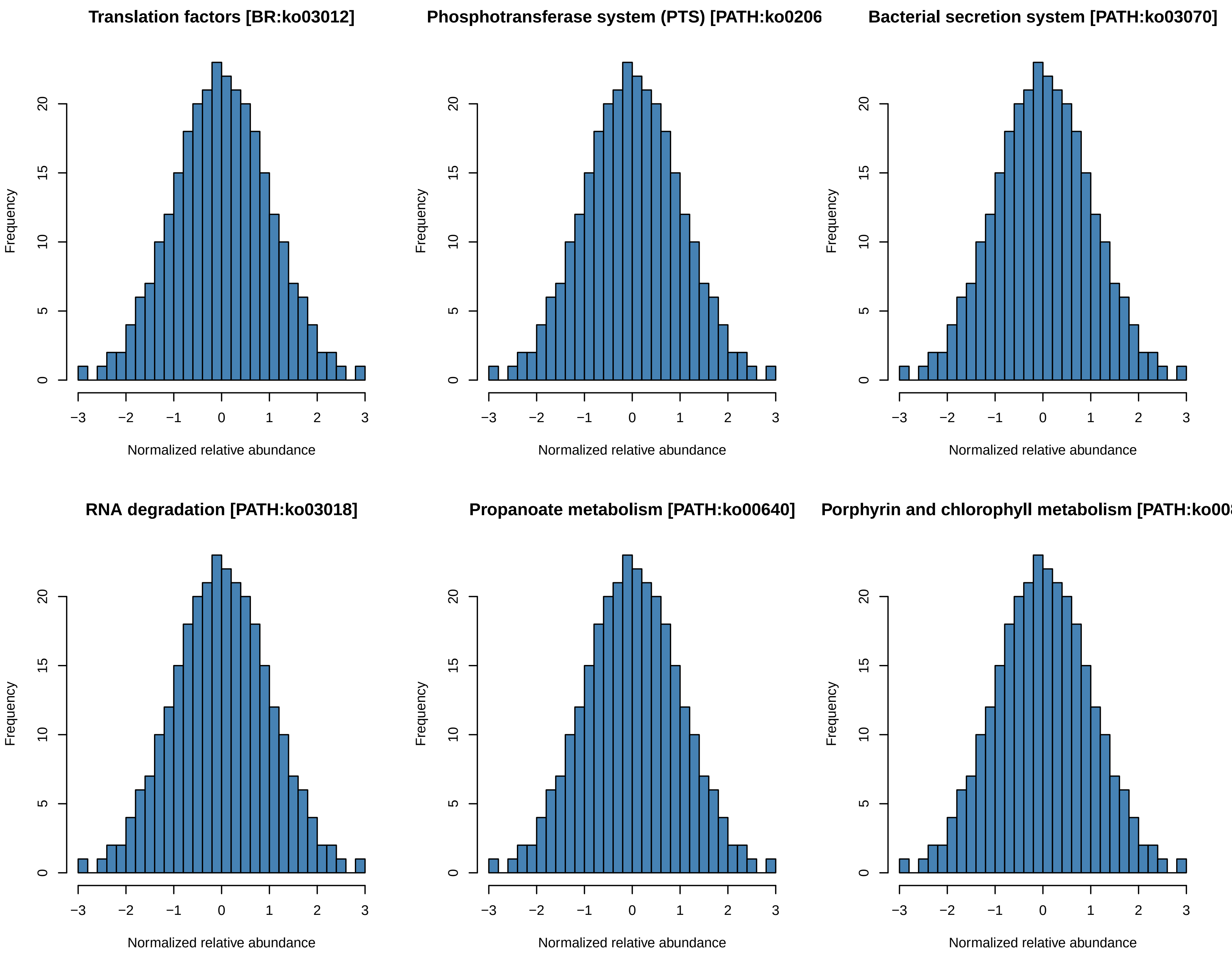

Translation factors [BR:ko03012]
Phosphotransferase system (PTS) [PATH:ko0206
Bacterial secretion system [PATH:ko03070]
RNA degradation [PATH:ko03018]
Propanoate metabolism [PATH:ko00640]
Porphyrin and chlorophyll metabolism [PATH:ko00
Frequency
Normalized relative abundance
0
5
10
15
20
−3
−2
−1
0
1
2
3

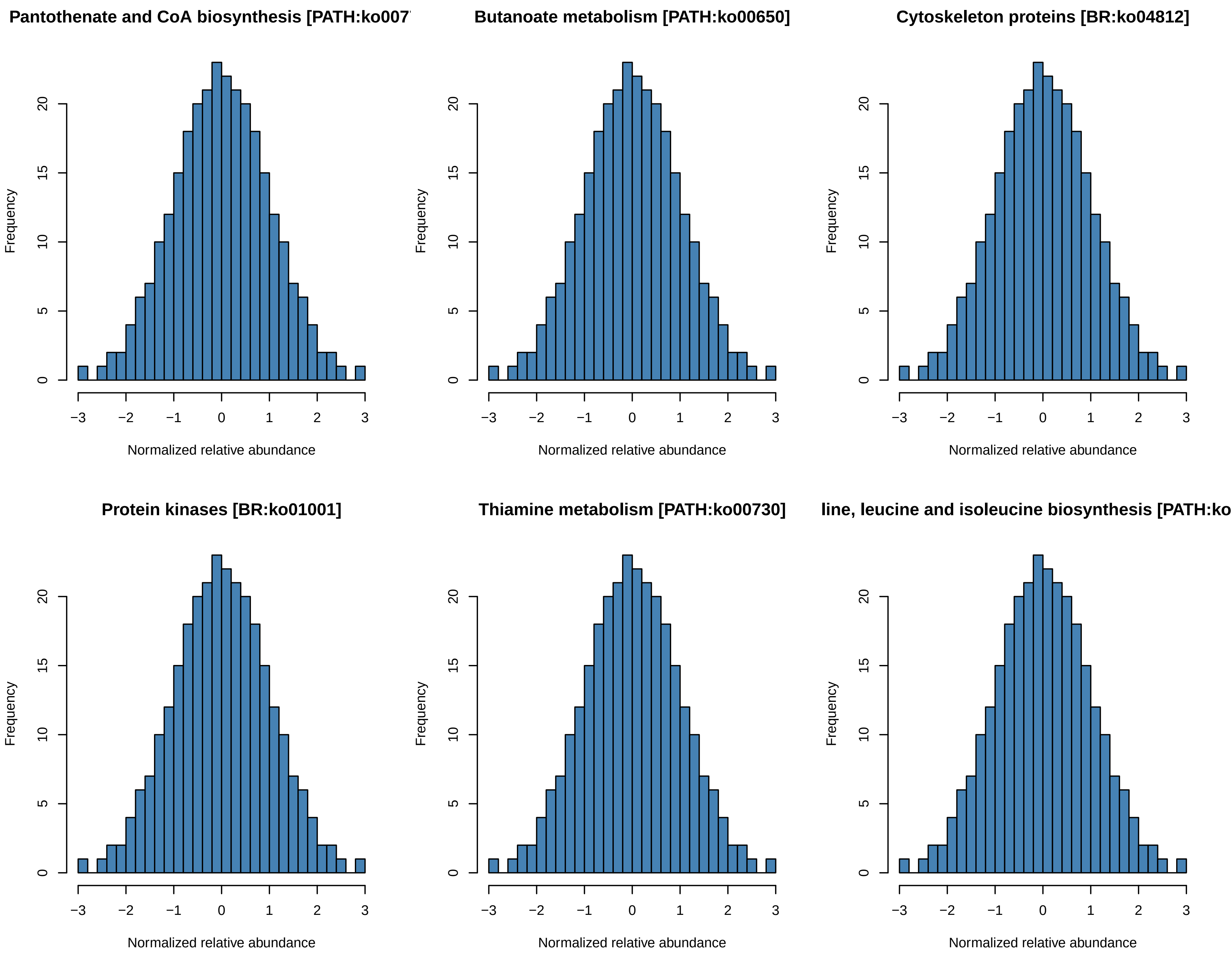

Pantothenate and CoA biosynthesis [PATH:ko007
Butanoate metabolism [PATH:ko00650]
Cytoskeleton proteins [BR:ko04812]
Protein kinases [BR:ko01001]
Thiamine metabolism [PATH:ko00730]
line, leucine and isoleucine biosynthesis [PATH:ko
Frequency
0
5
10
15
20
−3
−2
−1
0
1
2
3
Normalized relative abundance

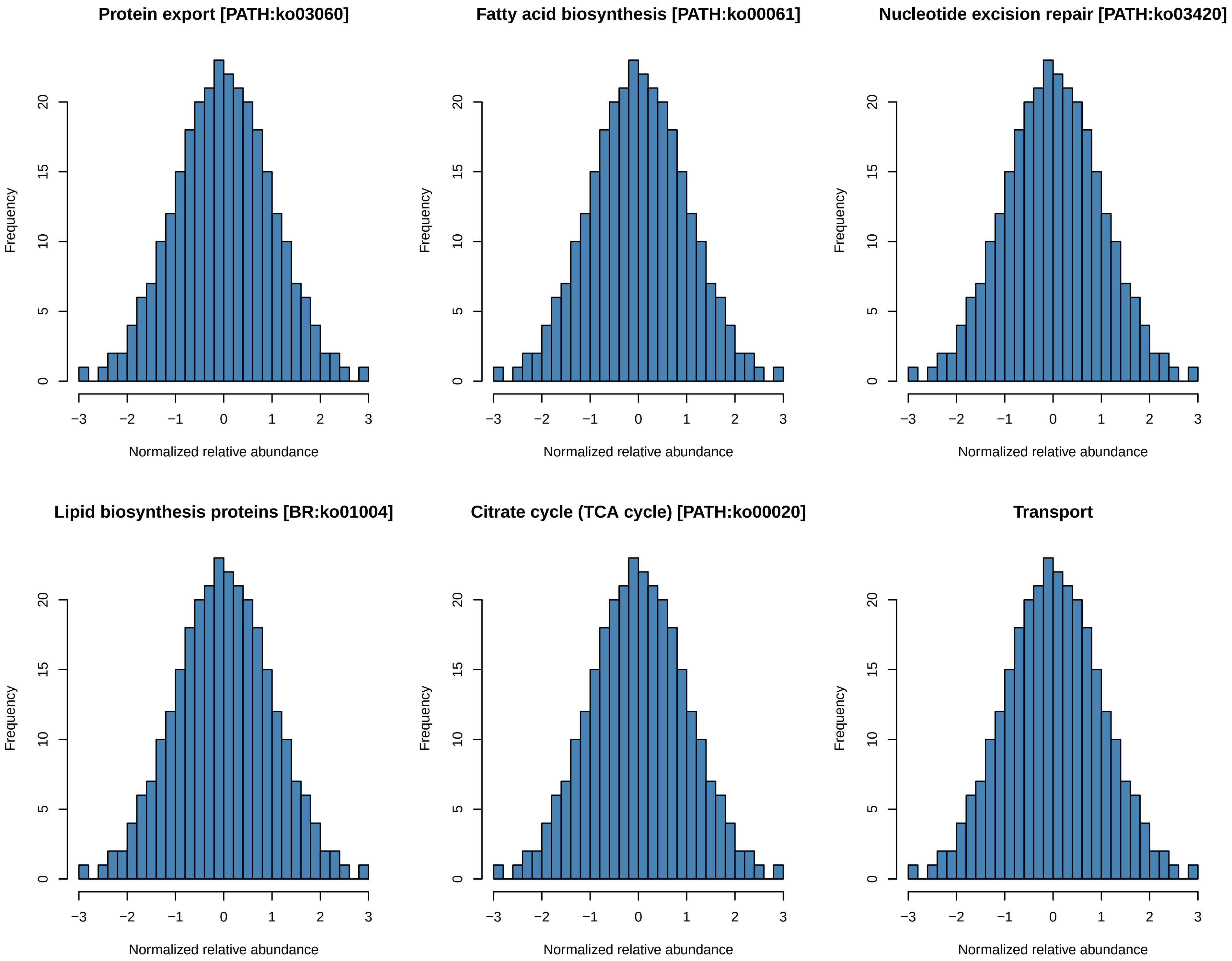
Protein export [PATH:ko03060]
Fatty acid biosynthesis [PATH:ko00061]
Nucleotide excision repair [PATH:ko03420]
Lipid biosynthesis proteins [BR:ko01004]
Citrate cycle (TCA cycle) [PATH:ko00020]
Transport
Frequency
0
5
10
15
20
−3
−2
−1
0
1
2
3
Normalized relative abundance

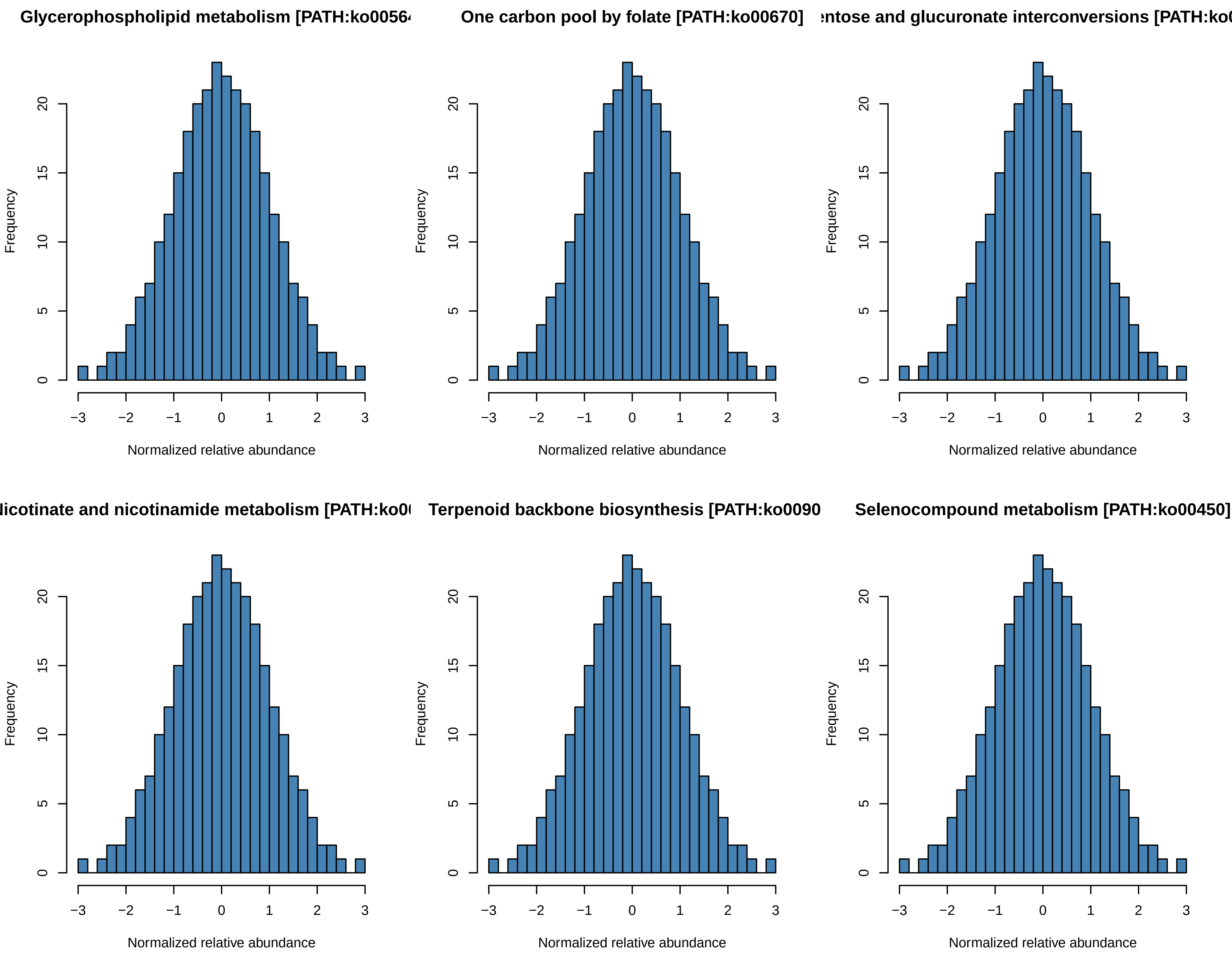
Glycerophospholipid metabolism [PATH:ko00564
One carbon pool by folate [PATH:ko00670]
entose and glucuronate interconversions [PATH:ko0
licotinate and nicotinamide metabolism [PATH:ko0(
Terpenoid backbone biosynthesis [PATH:ko0090
Selenocompound metabolism [PATH:ko00450]
Frequency
Normalized relative abundance
0
5
10
15
20
−3
−2
−1
0
1
2
3

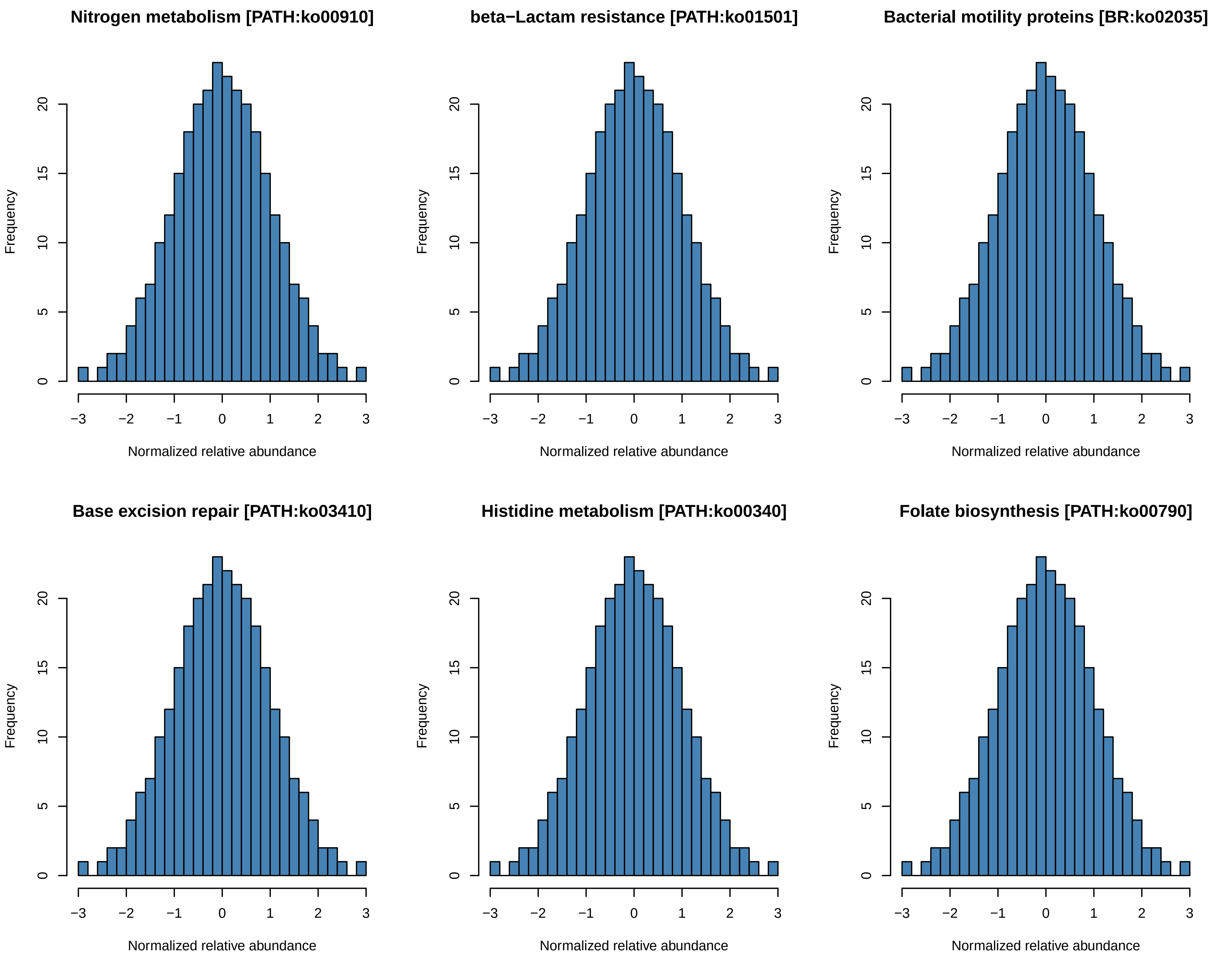
Nitrogen metabolism [PATH:ko00910]
beta−Lactam resistance [PATH:ko01501]
Bacterial motility proteins [BR:ko02035]
Base excision repair [PATH:ko03410]
Histidine metabolism [PATH:ko00340]
Folate biosynthesis [PATH:ko00790]
Frequency
0
5
10
15
20
−3
−2
−1
0
1
2
3
Normalized relative abundance

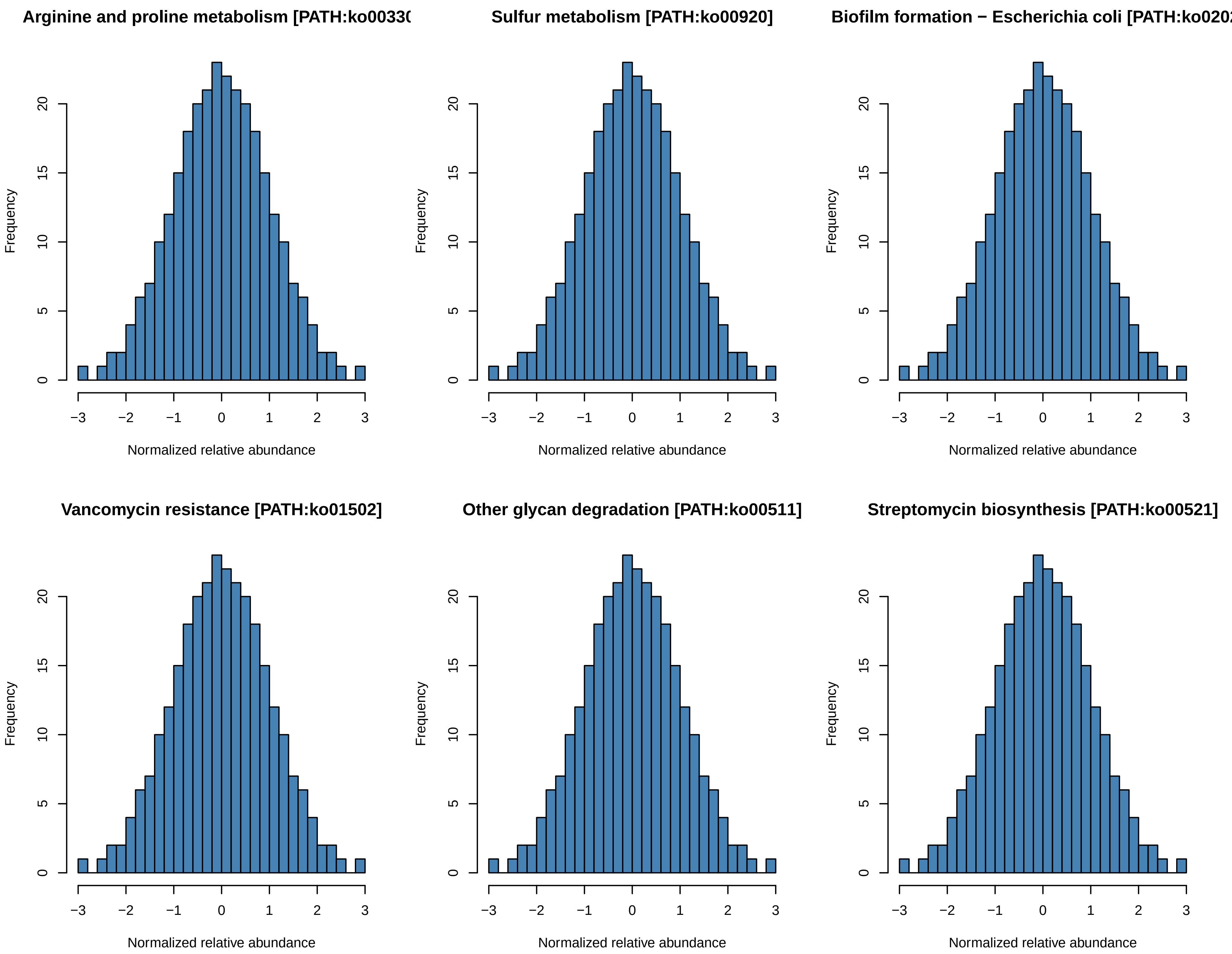
Arginine and proline metabolism [PATH:ko0033
Sulfur metabolism [PATH:ko00920]
Biofilm formation − Escherichia coli [PATH:ko020
Vancomycin resistance [PATH:ko01502]
Other glycan degradation [PATH:ko00511]
Streptomycin biosynthesis [PATH:ko00521]
Frequency
Normalized relative abundance
0
5
10
15
20
−3
−2
−1
0
1
2
3

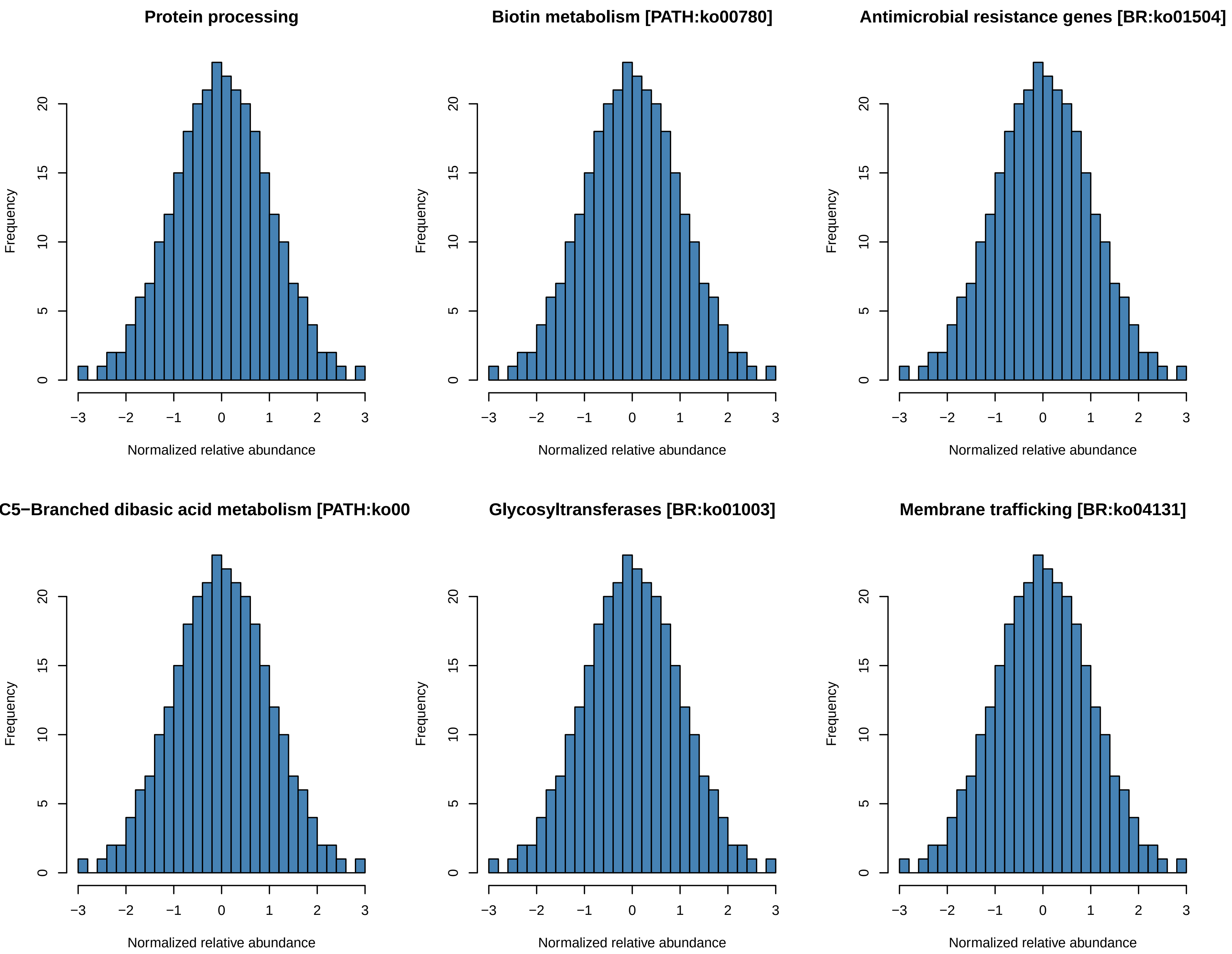
Protein processing
Biotin metabolism [PATH:ko00780]
Antimicrobial resistance genes [BR:ko01504]
C5−Branched dibasic acid metabolism [PATH:ko00
Glycosyltransferases [BR:ko01003]
Membrane trafficking [BR:ko04131]
Frequency
0
5
10
15
20
−3
−2
−1
0
1
2
3
Normalized relative abundance

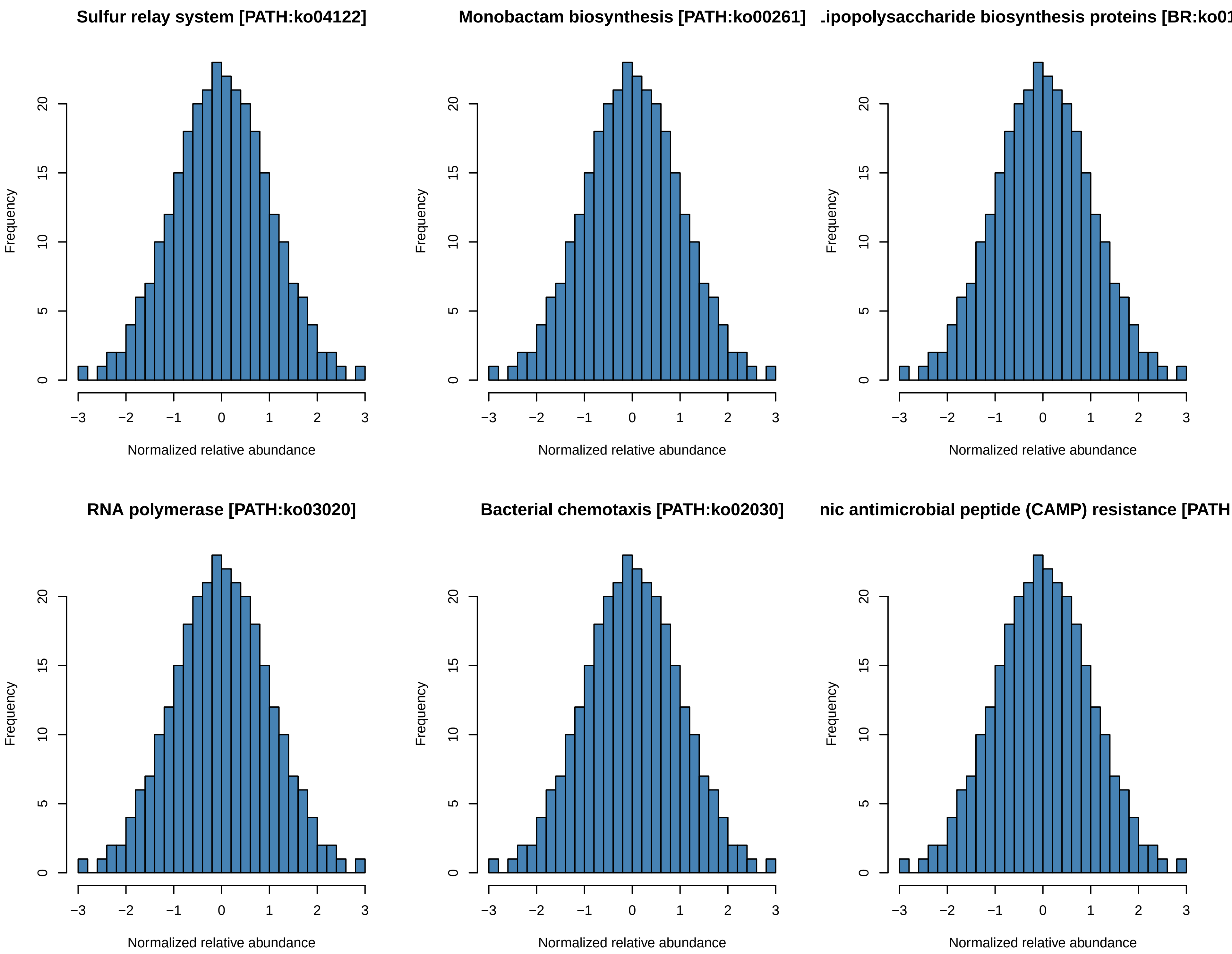
Sulfur relay system [PATH:ko04122]
Monobactam biosynthesis [PATH:ko00261]
.ipopolysaccharide biosynthesis proteins [BR:ko01
RNA polymerase [PATH:ko03020]
Bacterial chemotaxis [PATH:ko02030]
nic antimicrobial peptide (CAMP) resistance [PATH
Frequency
0
5
10
15
20
−3
−2
−1
0
1
2
3
Normalized relative abundance

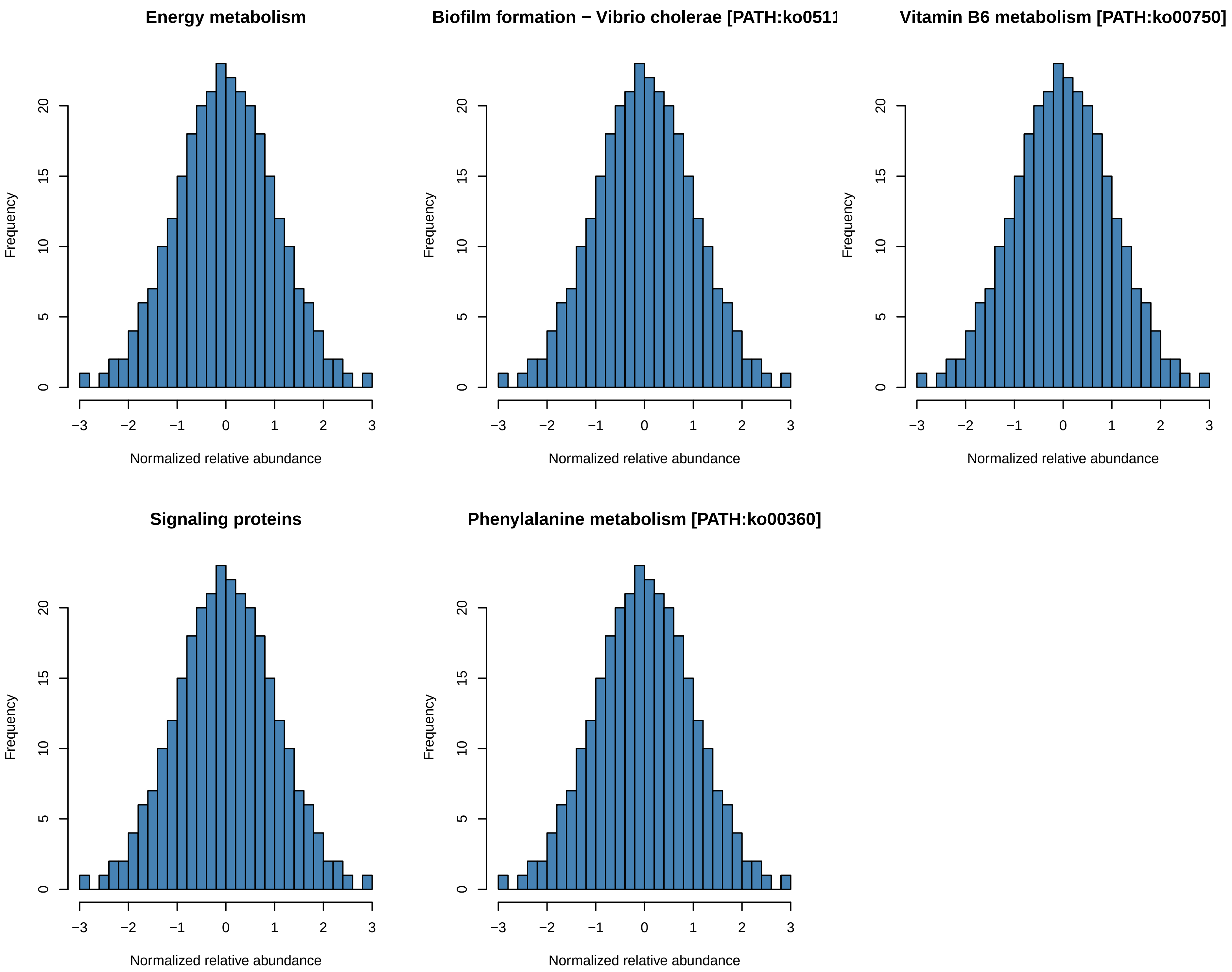

Energy metabolism
Biofilm formation − Vibrio cholerae [PATH:ko0511
Vitamin B6 metabolism [PATH:ko00750]
Signaling proteins
Phenylalanine metabolism [PATH:ko00360]
Frequency
0
5
10
15
20
−3
−2
−1
0
1
2
3
Normalized relative abundance
