# Supplementary material for: Interplay between host genetics and gut microbiome composition in the Japanese population
Source: Front Microbiomes. 2025 Oct 14;4:1635907. doi: 10.3389/frmbi.2025.1635907 (PMC12993688; doi:10.3389/frmbi.2025.1635907)
Supplement: Supplementary file 2 [file DataSheet2.pdf]

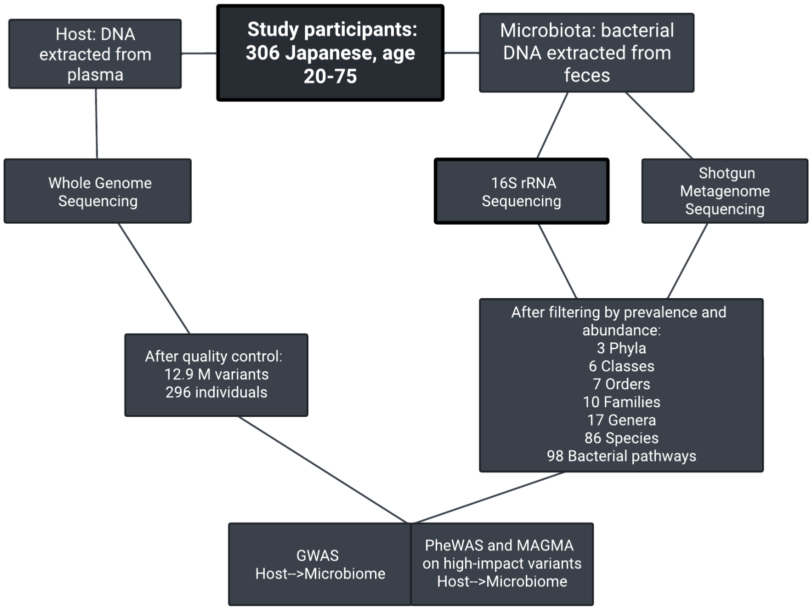

Supplementary Figure 1. Overview of the experimental workflow.

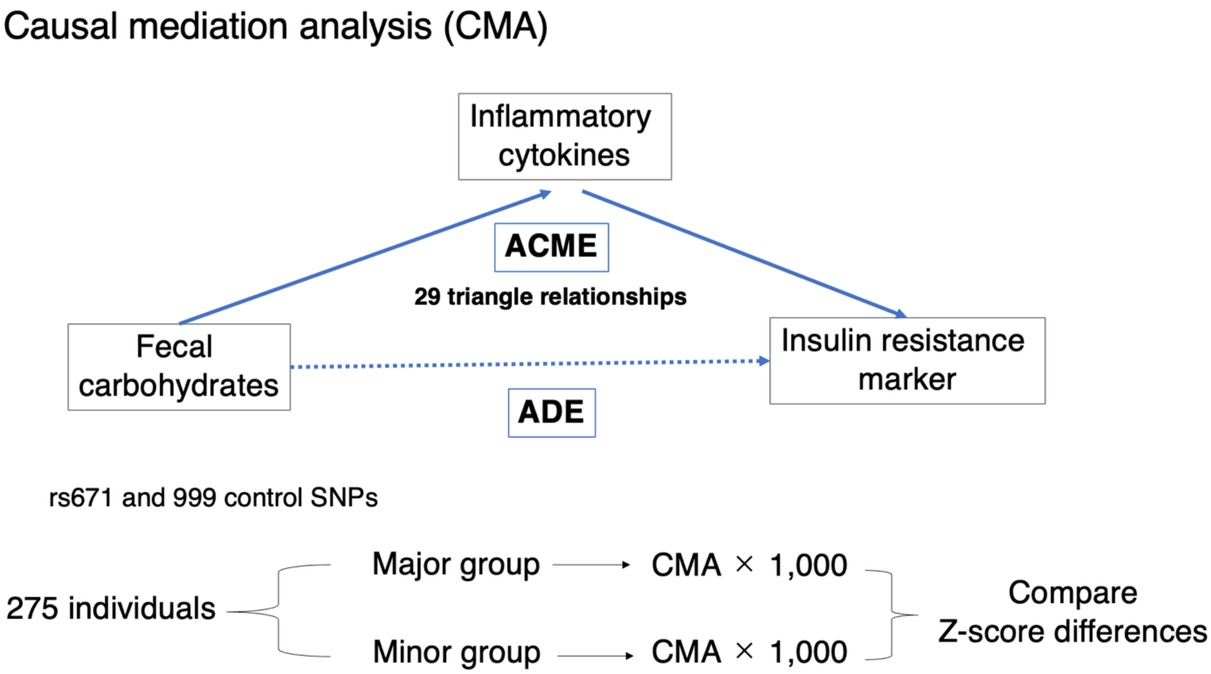

Supplementary Figure 2. Causal mediation analysis examining the relationships between fecal carbohydrates, inflammatory cytokines, and insulin resistance markers. It highlights 29 triangular relationships, focusing on the Average Causal Mediation Effect (ACME) and Average Direct Effect (ADE). The analysis involves 275 individuals categorized into major and minor genotype groups based on the rs671 variant and 999 control SNPs, with each group undergoing CMA analysis 1,000 times to compare Z-score differences.

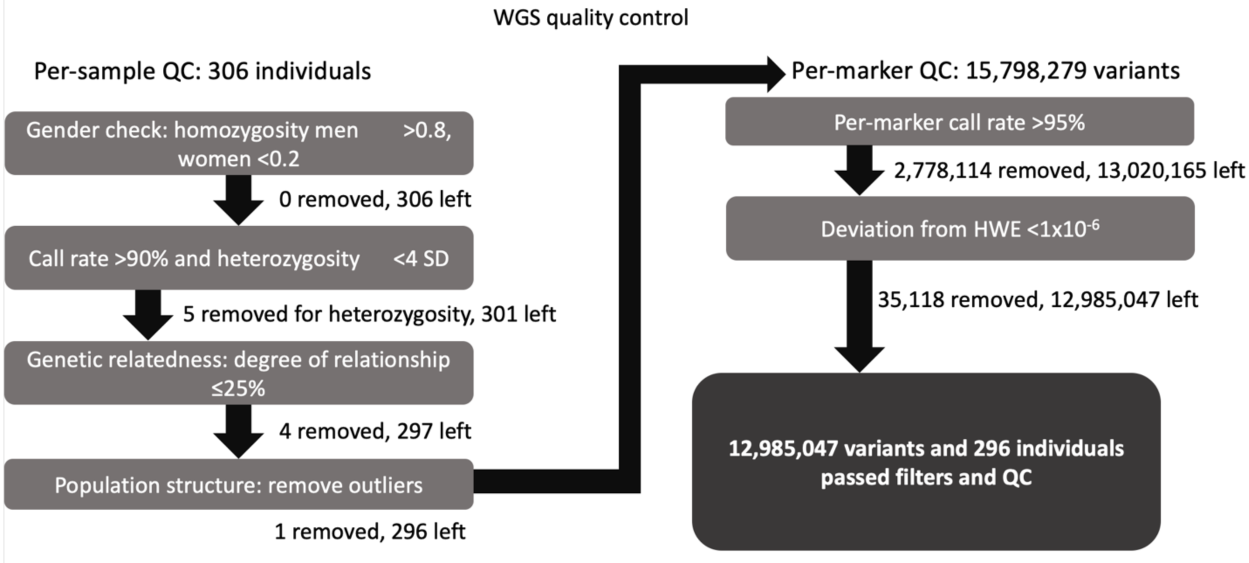

Supplementary Figure 3. Pipeline used for whole genome sequencing per-sample and per-marker quality control.

b

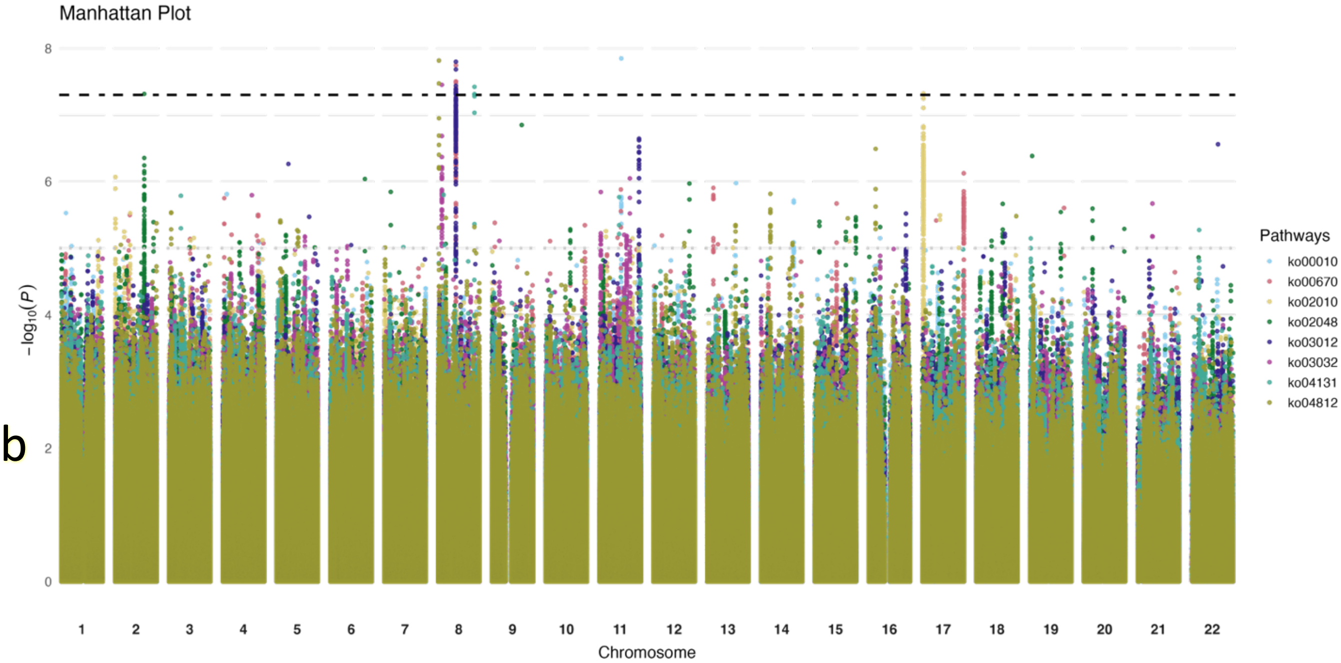

Supplementary Figure 4. Manhattan plot displaying the significant loci ($p < 5\times10^{-8}$) associated with bacterial pathways. Each color represents a distinct pathway.

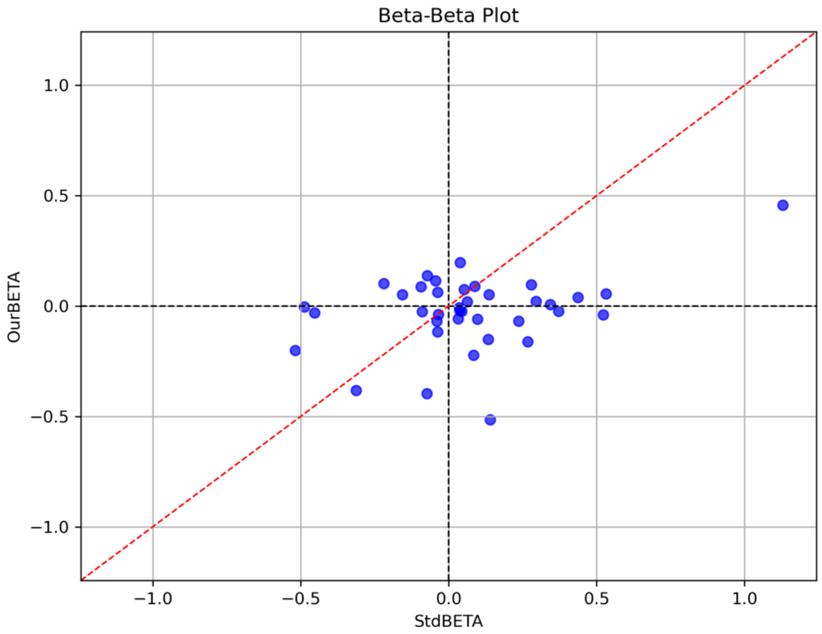

Supplementary Figure 5. Scatter plot comparing the beta coefficients between our GWAS, labeled as "OurBETA" (y-axis) and Ishida et al. [9] GWAS, labelled as "StdBETA" (x-axis). The diagonal dashed line represents the line of perfect agreement, where the beta coefficients from both analyses are equal.

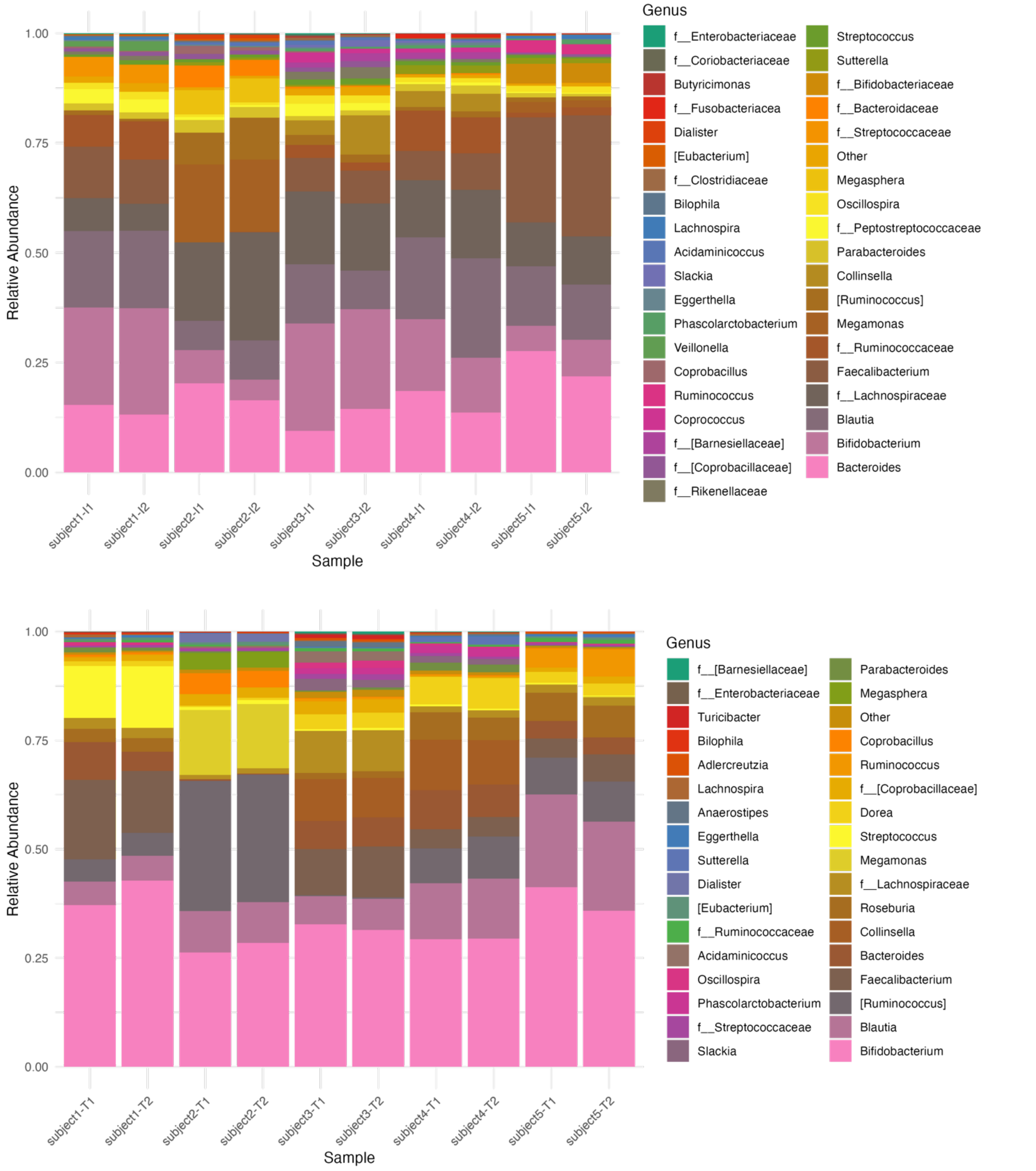

Supplementary Figure 6: Comparison of relative abundance composition between replicates in five samples using Ishida et al. (top figure) method and our method (bottom figure). "-I" indicates relative abundance by using Ishida et al. method, "-T" indicated the relative abundance of each sample by using our method. The y-axis shows the relative abundance of the bacteria, with each color in the stacked bar chart representing a different bacterial genus.

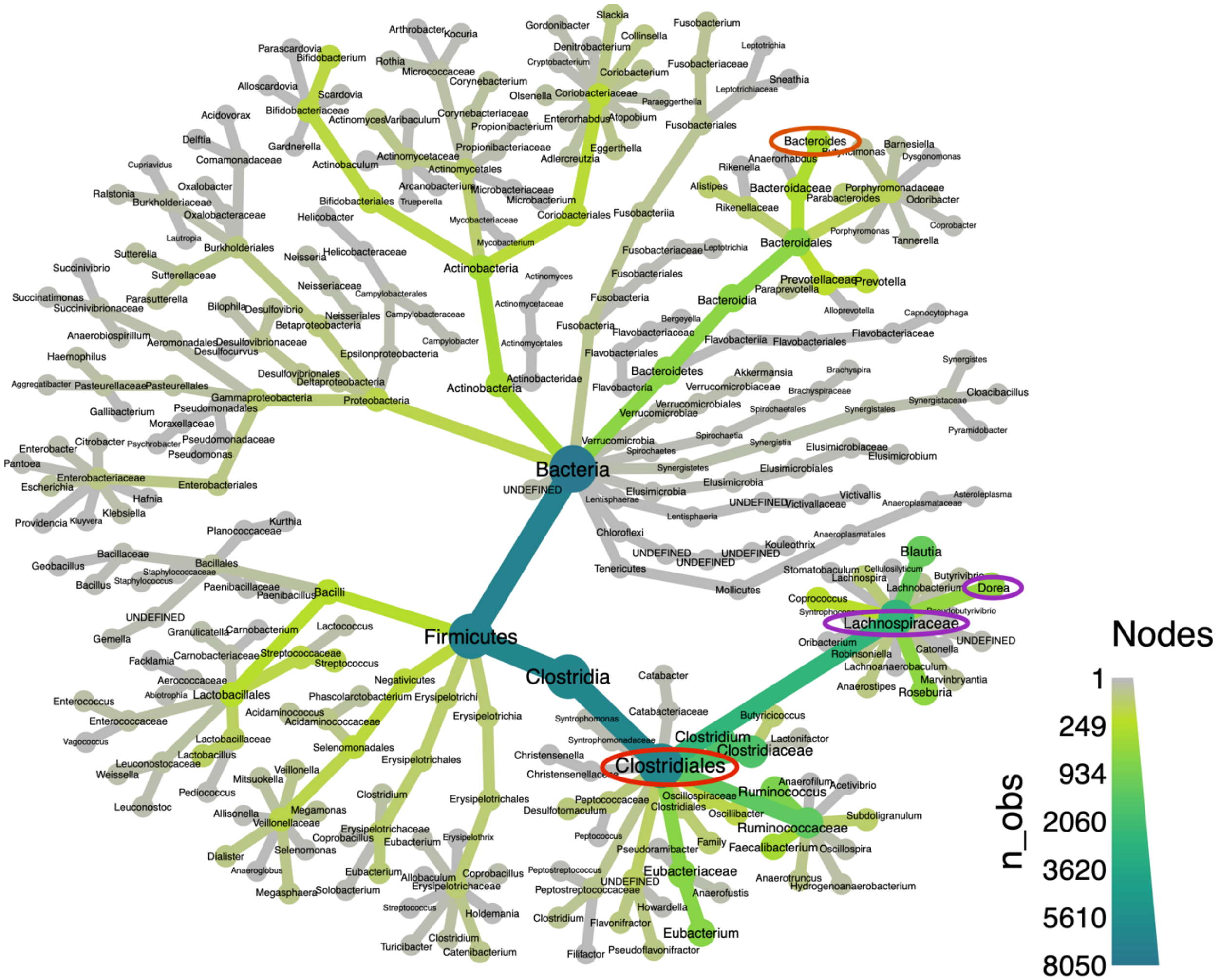

Supplementary Figure 7: Tree-based visualization of the taxonomic diversity. Tree was generated using all raw relative abundance data from all Genera in our cohort. Genera with significant associated variants are circled purple if associations were found through GWAS, red if found through binary GWAS, and orange if found through PheWAS. Taxonomic tree was created with Metacoder R package.
